# Electronically Ambivalent Hydrodefluorination of Aryl‐CF_3_ groups enabled by Electrochemical Deep‐Reduction on a Ni Cathode

**DOI:** 10.1002/anie.202218195

**Published:** 2023-02-10

**Authors:** John R. Box, Mickaël E. Avanthay, Darren L. Poole, Alastair J. J. Lennox

**Affiliations:** ^1^ School of Chemistry University of Bristol Cantock's Close Bristol BS8 1TS UK; ^2^ Discovery High-Throughput Chemistry Medicinal Chemistry GSK Medicines Research Centre Stevenage SG1 2NY UK

**Keywords:** Defluorination, Electrochemistry, Fluorine, Nickel, Reduction

## Abstract

The Ar‐CF_2_H moiety is featured in an increasing number of bioactive compounds due to its unique combination of properties. The hydrodefluorination of Ar‐CF_3_ compounds is a direct and efficient route toward this motif. As reported methods for this transformation have focused on specific substrate families, herein we describe a general—electronically ambivalent—procedure for the single‐step direct mono‐hydrodefluorination of a variety of feedstock and functionalized Ar‐CF_3_ compounds. Exploiting the inherent tunability of electrochemistry and the selectivity enabled by a Ni cathode, the deep reduction garners high selectivity for ArCF_*2*_H products, with good to excellent yields up to gram scale. The protocol has been extended to a single‐step *di‐*hydrodefluorination yielding benzyl fluorides. The late‐stage peripheral editing of a single CF_3_ feedstock to construct fluoromethyl (CF_2_H, CFH_2_) moieties will aid the rapid diversification of lead‐compounds and compound libraries.

We report a general procedure for the direct *mono‐* and *di*‐hydrodefluorination of ArCF_3_ compounds. Exploiting the tunability of electrochemistry and the selectivity enabled by a Ni cathode, the deep reduction garners high selectivity with good to excellent yields up to gram scale. The late‐stage peripheral editing of CF_3_ feedstocks to construct fluoromethyl moieties will aid the rapid diversification of lead‐compounds and compound libraries.

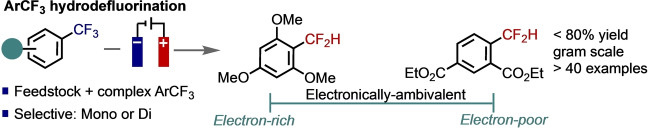

The difluoromethyl group has been the subject of much recent attention in drug and agrochemical design.[^1^, ^2^, ^3^, ^4^, ^5^, ^6^] In particular, difluoromethylarenes (ArCF_2_H) are incorporated as lipophilic[^7^, ^8^] hydrogen‐bond donors,[^9^, ^10^] a unique combination of properties that provides discovery chemists with a robust, non‐nucleophilic bioisostere for OH and SH groups.[^2^, ^10^, ^11^, ^12^] While not as prevalent as trifluoromethylarenes (ArCF_3_),[^3^, ^13^] the frequency of ArCF_2_H moieties reported in biologically‐relevant systems has exploded over the last decade.^[14]^ Intramolecular H‐bonding in a CF_2_H‐pyrazole containing fungicide provides structural rigidity and enhances potency,^[9]^ Figure 1A, as well as intermolecular H‐bonding^[11]^ in biological systems.^[15]^ The ability to modulate logP^[16]^ and p*K*_a_ ^[17]^ are also enabled by a C−F to C−H switch.

**Figure 1:**
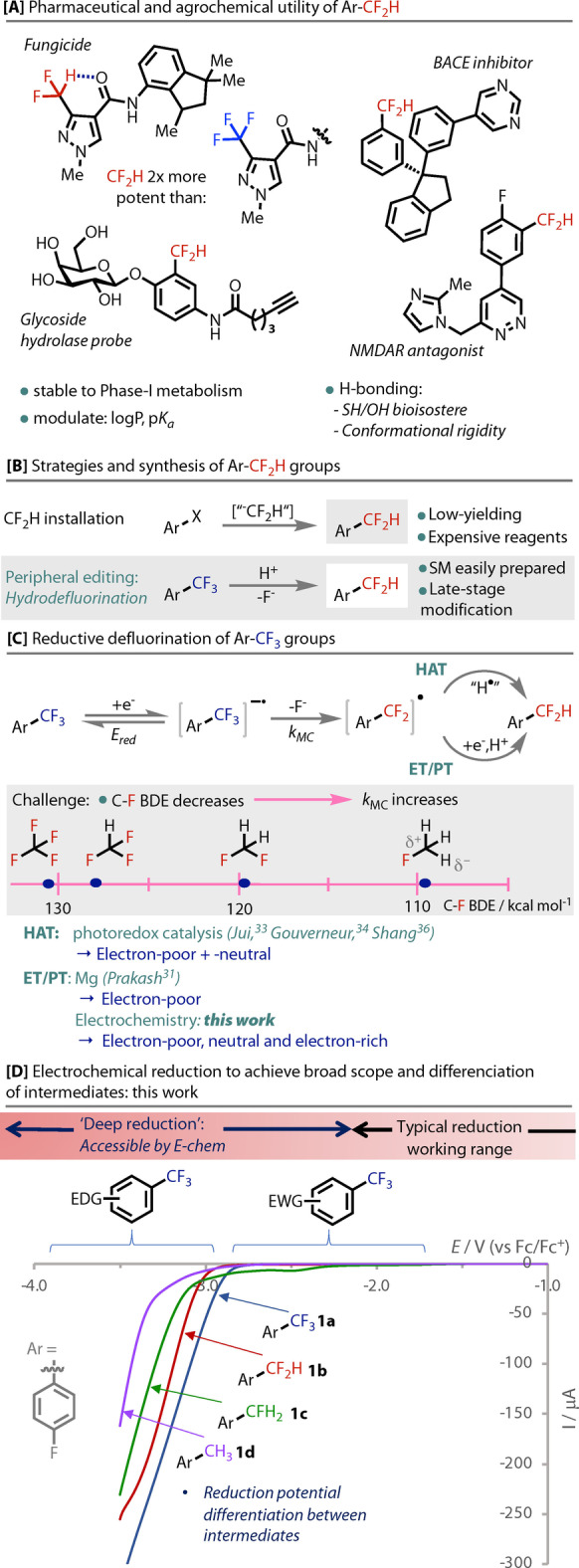
[**A**–**C]**: The utility of CF_2_H groups, their synthesis and challenges; [**D]**: Electrochemical defluorination strategy. CV of **1 a**–**d** (averaged forward and return currents). ‐Ni||+Pt, 5 mM [**1 a**–**d**], degassed DMF, 0.1 V/s, NBu_4_PF_6_, N_2_. See Supporting Information for CVs run in with different electrode materials and in MeCN.

The requirement for rapid syntheses amenable to late‐stage intermediates has initiated a shift toward molecular editing strategies.[^18^, ^19^] Hence, while the direct difluoromethylation of prefunctionalized substrates is known, albeit with limited scope, or expensive/impractical reagents,[^20^, ^21^] editing the more easily installed and traditionally inert CF_3_ group has synthetic and strategic advantages, Figure 1B. Pioneering work from Perichon,[^22^, ^23^, ^24^] Savéant[^25^, ^26^] and Bordeau[^27^, ^28^] established a route toward their functionalization through single electron reduction that initiates mesolytic C−F bond cleavage.^[29]^ As the CF_3_ group progressively defluorinates, the C−F bond strength decreases[^30^, ^31^, ^32^, ^33^] and the rate of mesolytic cleavage increases, Figure 1C. This favours further defluorination, as encountered in early electrochemical attempts where total defluorination could not be avoided.[^24^, ^34^, ^35^, ^36^] Nevertheless, the use of excess Mg as reductant was reported for the hydrodefluorination of the readily reducible *bis*(CF_3_) arenes under acidic conditions.^[37]^ The reductant in this 2‐electron defluorination/protonation (ET/PT, Figure 1C) strategy must preferentially reduce the ArCF_3_ and avoid direct proton reduction, a compromise that naturally limits the scope of amenable ArCF_3_ substrates.^[38]^ Broader scope and functional group tolerance has since been achieved by Jui,^[39]^ Gouverneur,^[40]^ and Shang,^[41]^ who rely on SET from an excited organophotocatalyst and then hydrogen atom transfer (HAT, Figure 1C). Although good hydrodefluorination yields and improved selectivity are reported, each system is specific to a set range of arene electronics, as the reduction potential window is determined by the photocatalyst/H‐atom donor system.[^42^, ^43^] Hence, a method that is broadly applicable on a range of electronically‐variable ArCF_2_H compounds remains elusive.

Electrochemistry has the innate ability to safely apply deep‐reduction potentials (<−2.0 V vs Fc/Fc^+^). Hence, compounds can be accessed that require reduction potentials outside the range of traditional chemical reductants and excited state photoredox catalysts. As the reduction potential of each intermediate becomes more negative with each defluorination, Figure 1D, by applying only the minimum required potential, the intermediates can be differentiated and over defluorination is easier to avoid. Herein, we detail our successful efforts in the development of a selective electrochemical *mono‐* and *di‐*hydrodefluorination strategy on a broad range of electronically variable substrates.

The primary challenge in the development of this reaction was to accommodate the deeply reducing potentials required while avoiding over‐reduction and competing proton reduction. Investigations initially focused on the electronically neutral Ar‐CF_3_ substrate **1 a** (*E*_red(onset)_ −2.8 V vs Fc/Fc^+^). A divided cell was adopted with a reductively stable electrolyte system (MeCN, *E*_window_ +2.7–−3.4 V *vs* Fc/Fc^+^ with Et_4_NPF_6_).^[44]^ Following extensive optimization studies (see Table 1 and Supporting Information), 76 % of ArCF_2_H **1 b** was achieved with 25 : 1 selectivity for the *mono*‐hydrodefluorinated **1 b** *vs di*‐hydrodefluorinated benzyl fluoride **1 c** (entry 1). It was found that the inclusion of the fluoride trap, TMSCl, enhanced the yield and selectivity (entry 1 vs 2). In place of sacrificial metal anodes, which can competitively reduce and plate the cathode at deep reduction potentials, Bu_4_NBr was used as a suitable organic reductant, as previously discovered in our trifluoromethylketone hydrodefluorination work.^[45]^ As the oxidation product (Br_3_^−^) is anionic, it resists migration to the cathodic chamber and any subsequent short circuiting that would lead to a decline in faradaic efficiency. Application of 1 *F* gave almost ideal efficiency (max=50 %) and selectivity (entry 3). Bu_4_NCl was also suitable but gave lower selectivity (entry 4). The most important factor to achieving high selectivity on this substrate was the choice of a nickel foil working electrode (entries 5–7).^[46]^ Ni was found to give higher yields than Pt or graphite and superior selectivity, halting at **1 b** after 2 *F*. Extensive electrode fouling and physical decomposition of graphite electrodes were observed at these deep reduction potentials. Anhydrous MeCN was required to avoid competing reduction of either water (or HCl formed from the hydrolysis of TMSCl). With the exception of TBAClO_4_, alternative reductively stable solvents or supporting electrolyte salts did not lead to any desired reaction (entries 8–12); competing K^+^ reduction (*E*_red_=−2.27 V vs Fc/Fc^+^)^[47]^ likely decreases efficiency with KPF_6_ (entry 11).

**Table 1:** Hydrodefluorination optimisation.^[a]^

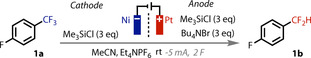			
Entry	Variation from optimized conditions (above)	Yield [%]	Selectivity CF_2_H : CFH_2_ (**1 b** : **1 c**)
1	None	76	25 : 1
2	No TMSCl	64	7 : 1
3	1 *F* not 2 *F*	47	>25 : 1
4	Bu_4_NCl not Bu_4_NBr	71	10 : 1
5	+ Ni foil not Pt	50	20 : 1
6	– Pt not Ni foil	72	5 : 1
7	– C not Ni foil	20	5 : 1
8	Me‐THF not MeCN	n.d	n/a
9	DMF not MeCN	n.d	n/a
10	Me_3_PhNPF_6_ not Et_4_NPF_6_	<5 %	n/a
11	Bu_4_NClO_4_ not Et_4_NPF_6_	71	20 : 1
12	KPF_6_ not Et_4_NPF_6_	n.d	n/a

[a] See Supporting Information for full details. ^19^F NMR yields. Ratios in brackets correspond to ArCF_2_H : ArCFH_2_ determined from crude ^19^F NMR, n.d.=not‐detected.

With optimized conditions for **1 a**, we tested their amenability to a range of electronically variable ArCF_3_ substrates, Figure 2A. Hence, a small collection of electron‐neutral, ‐poor and ‐rich rings were tested under our conditions, as well as in four published state‐of‐the‐art conditions. Electron neutral substrates **1 a**–**4 a** were all well tolerated by our conditions, returning products in good to excellent yields. Yields from the reported methods for this family of substrates were generally comparable to ours. Electron‐poor substrate **5 a** transferred smoothly to **5 b**, without ester reduction, comparing well to the Gouverneur conditions [A] that are specific to electron‐poor substrates. However, conditions [B–D] did not provide any product **5 b**. Pleasingly our electrochemical conditions could tolerate electron‐rich substrates **6 a**–**8 a**, including tris(methoxy)phenyl **8 a**, whilst reported conditions generally did not convert any of these substrates. CV analysis showed that **6 a** and **8 a** required a reduction potential ≈160 mV and 500 mV deeper, respectively, than **1 a**. Finally, pyrazole **7 a** was amenable, while all reported methods gave no **7 b**.

**Figure 2:**
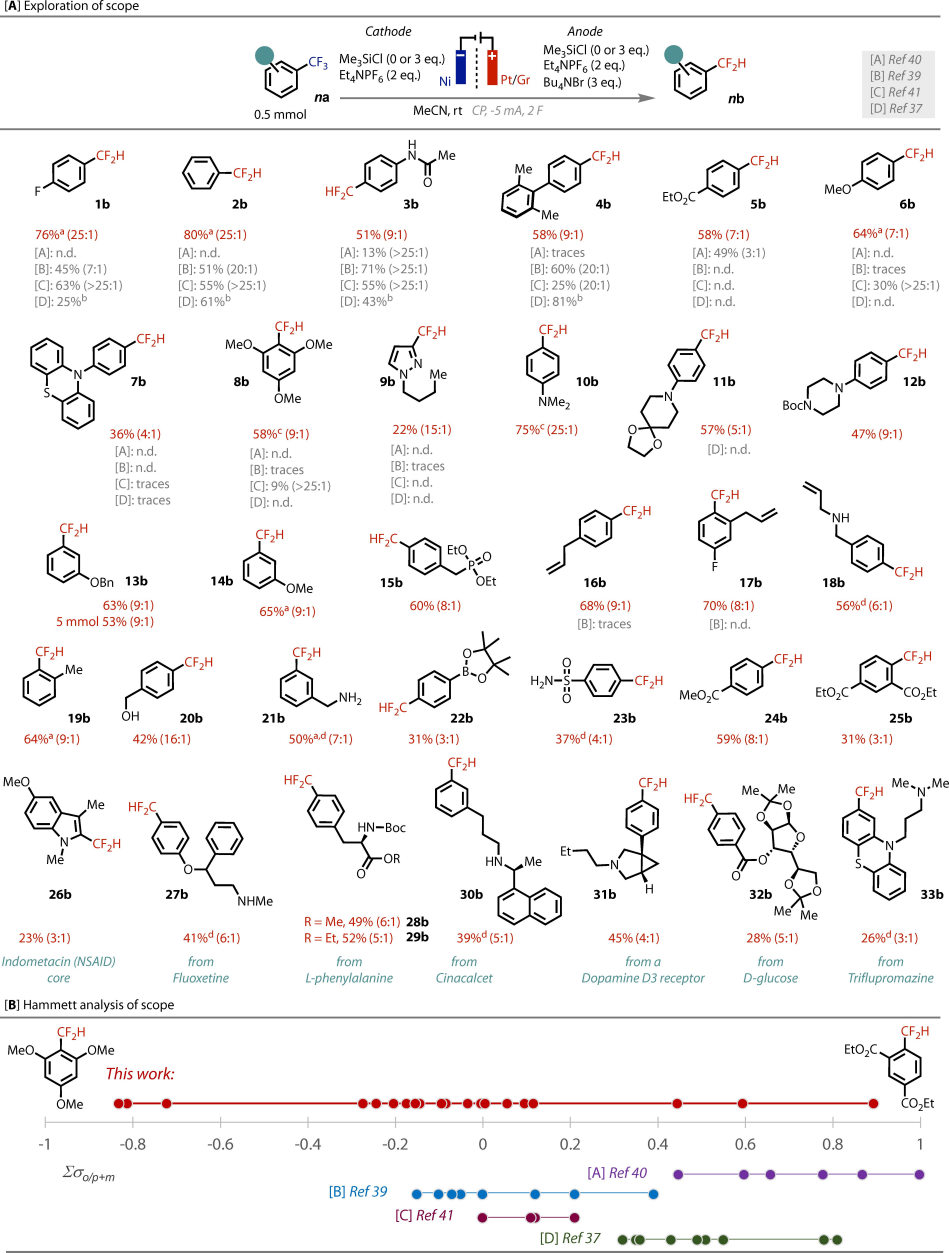
[**A**]: Substrate scope. Isolated yields of ArCF_2_H, unless indicated. Ratios in brackets correspond to CF_2_H:CFH_2_ determined from crude ^19^F NMR, n.d.=not‐detected. CP=Chronopotentiometry. Benchmarking yields are ^19^F NMR yields, except where noted ‘b’. ^a 19^F NMR yield, not isolated due to volatility. ^b^ % conversion, ^c^ CF_2_H observed in ^19^F NMR of crude mixture, product hydrolysed to corresponding aldehyde on silica, ^d^ reaction run without TMSCl. [**B**]: Summed Hammett σ value of successful substrates (see Supporting Information for details).

Encouraged by this, an extensive substrate scope of both commodity and functionalized ArCF_3_ was undertaken. Several other electron‐rich rings were tested, including aniline derivatives (**10**–**12 b**) and with ether (**13**–**14 b**) or alkyl (**15**–**21 b**) substitution, all of which were well tolerated. More substrates with electron‐poor rings (**22**–**25 b**) were tested and found to transform in moderate to good yields, including highly electron‐deficient bis‐ester **25 a**. We tested the conditions on more complex drug‐type molecules (**26**–**33 b**). Pleasingly, all these compounds were tolerated and gave products in moderate to good yields, demonstrating the capability of this procedure to previously unreported substrate classes and to late‐stage functionalization. Analysis of our scope by the total electronic contribution of the substituents revealed the electronic ambivalence of our method,^[48]^ (see Supporting Information) Figure 2B.

Several functional handles were well tolerated in the scope, which is essential for diversification and application as building blocks. Such handles include ester, pinacol boronic ester, alkenes, primary alcohols, primary, secondary and tertiary amines, phosphonate and sulfonamides. For nucleophilic groups, such as amines, the exclusion of TMSCl facilitated a more efficient reaction, possibly avoiding competitive reduction of HCl generated from the formation of a N‐TMS adduct.^[28]^ Acetal, ketal and Boc protecting groups were also tolerated, as well as extended ring systems, which can be affected by electrode grafting.[^46^, ^49^] Functionality that was not tolerated includes halides (except fluoride), nitro and cyano groups, which can be reductively cleaved (see Supporting Information). We successfully scaled the reaction 10‐fold to a gram scale (5 mmol) without a significant decrease in yield or selectivity of product **13 b**.

To gain insight into the mechanism and to probe for radical intermediates (ArCF_2**⋅**_), two radical trap experiments were conducted (Figure 3A). When introducing either an intermolecular (**1 a** + amylene) or intramolecular alkene (**17 a**) to the system, no products expected from the addition of ArCF_2**⋅**_ to an alkene were observed, suggesting a rapid second reduction to the ArCF_2_^−^ anion.[^35^, ^36^] This contrasts the photochemical HAT methods,^[50]^ where transient ArCF_2**⋅**_ radicals trap alkenes, *c.f*. conditions [B] returned no **17 b**, Figure 2A. These data provide evidence for a rapid transfer of 2 electrons through an ECEC mechanism (ET/PT, Figure 1C). As the reaction solution is required to be anhydrous, we were intrigued to probe the origin of the proton. Our previous studies on electrochemical C−F activation found evidence for protonation from the NEt_4_^+^ cation and not from the solvent.^[45]^ However, conducting this reaction in anhydrous D_3_‐MeCN, **1 a** was smoothly transformed to D_1_‐**1 b** (91 % D‐incorporation). Though the p*K*_a_ has not been directly determined (due to F^−^ elimination),[^51^, ^52^] indirect evidence suggests the ArCF_2_^−^ anion is sufficiently basic to deprotonate MeCN.^[53]^

**Figure 3:**
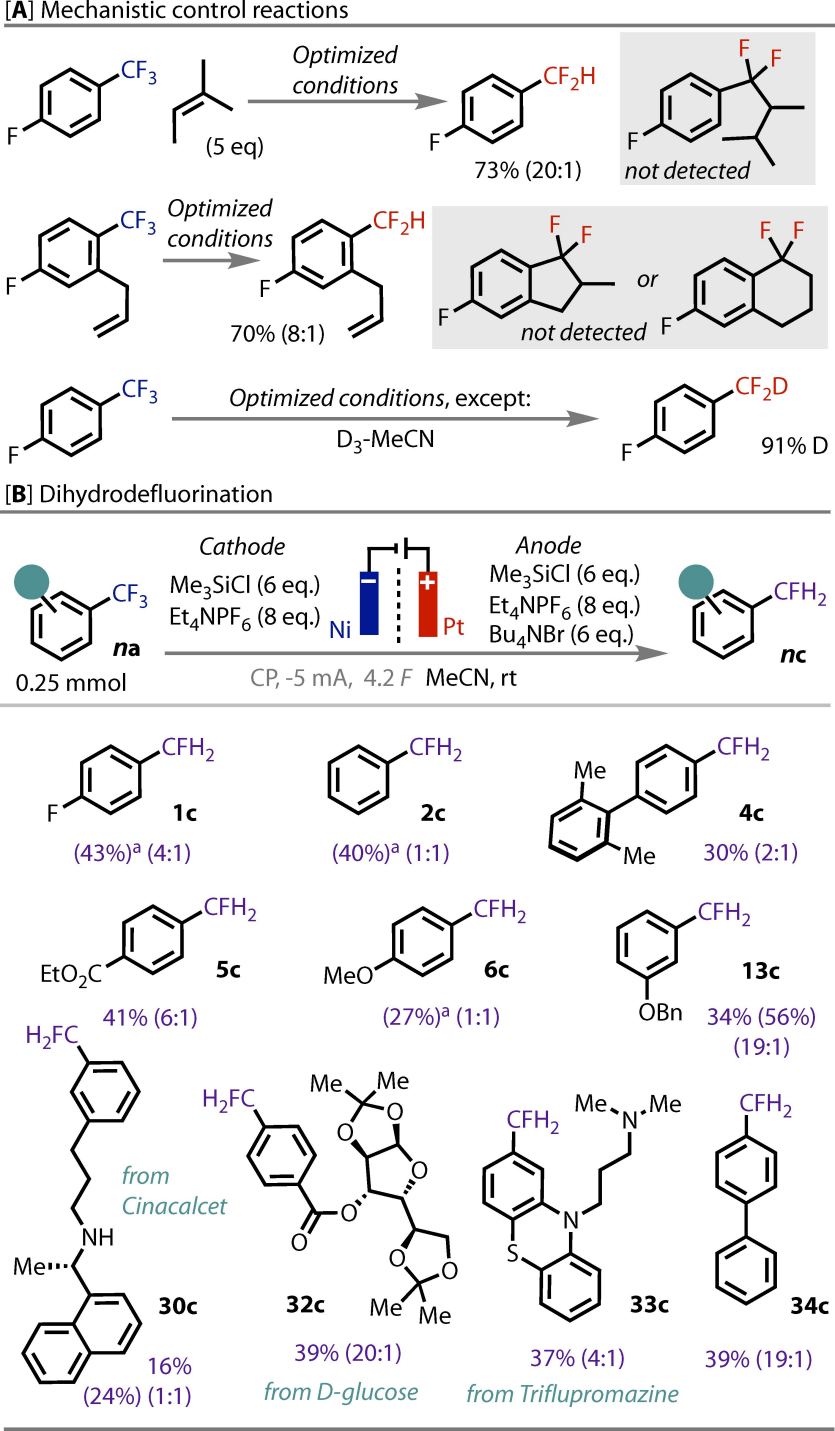
[A] control experiments to probe for radical or anionic intermediates. [B] ArCFH_2_ scope, isolated yields of ArCFH_2_, with ^19^F NMR yields given in parentheses, ratio is CFH_2_ : CF_2_H. ^a^ NMR yield given due to volatility of product.

Motivated by this success, efforts were directed toward the development of direct ArCF_3_ *di‐*hydrodefluorination. There are currently no prior reports for this challenging transformation. Nevertheless, we were encouraged when a purified sample of **1 b** was transformed under the reaction conditions to **1 c** in a good yield (see Supporting Information). As such, a more user friendly one‐pot procedure from ArCF_3_ was targeted. After decreasing the substrate concentration, increasing the current density, and passing 4 *F*, a satisfying yield and selectivity was achieved for product **1 c**, Figure 3C. A modest scope of substrates was examined, demonstrating moderate to good yields of ArCFH_2_ products.^[54]^ Considering how deep the required reduction potentials are to effect this 4‐electron/2‐proton transformation, Figure 1D, the substitution tolerated was varied, and complex biologically relevant molecules were converted (**2**–**34 c**). Of additional interest, biaryl **34 a** was better tolerated in the *di*‐ than in the *mono*‐hydrodefluorination, which is likely due to resonance stabilization of the intermediate radical anion.[^55^, ^56^]

In conclusion, we have developed an electronically ambivalent *mono‐*hydrodefluorination reaction of readily accessed ArCF_3_ substrates. The reactivity and selectivity are enabled by the electrochemical reduction on a Ni cathode at deeply reducing potentials. A broad range of ArCF_2_H compounds are prepared, including pharmaceutically and biologically relevant targets. The methodology is shown to be scalable for med‐chem use, and evidence was gained for an ECEC mechanism. The strategy was extended to a *di*‐hydrodefluorination reaction, yielding ArCFH_2_ compounds. Hence, the rapid construction of fluoromethyl motifs from ArCF_3_ groups up‐values an inexpensive, readily‐available feedstock to more desirable and functionally diverse building blocks, which should aid SAR studies and library synthesis.

## Conflict of interest

The authors declare no conflict of interest.

## Supporting information

As a service to our authors and readers, this journal provides supporting information supplied by the authors. Such materials are peer reviewed and may be re‐organized for online delivery, but are not copy‐edited or typeset. Technical support issues arising from supporting information (other than missing files) should be addressed to the authors.

Supporting Information

## Data Availability

The data that support the findings of this study are available in the supplementary material of this article.
