# Supplementary material for: Electronically Ambivalent Hydrodefluorination of Aryl‐CF3 groups enabled by Electrochemical Deep‐Reduction on a Ni Cathode
Source: Angew Chem Int Ed Engl. 2023 Feb 10;62(12):e202218195. doi: 10.1002/anie.202218195 (PMC10946569; doi:10.1002/anie.202218195)
Supplement: Supplementary file 1 — Supporting Information [file ANIE-62-0-s001.pdf]

Angewandte Chemie
Eine Zeitschrift der Gesellschaft Deutscher Chemiker

Supporting Information

Electronically Ambivalent Hydrodefluorination of Aryl-CF_3 groups enabled by Electrochemical Deep-Reduction on a Ni Cathode

*J. R. Box, M. E. Avanthay, D. L. Poole, A. J. J. Lennox**

Contents

General Experimental Details 5
Prevalence of $ArCF_2H$ in pharmaceutically-relevant compounds 7
Extended Optimisation Table.. 8
Role of TMSCl 9
CV and SWV Studies 12
Cyclic voltammograms 12
Square wave voltammetry 17
Benchmarking details 18
Substrate Synthesis and Characterisation 22
2,6-Dimethyl-4'-(trifluoromethyl)-1,1'-biphenyl, **4a** 22
1,3,5-trimethoxy-2-(trifluoromethyl)benzene, **8a** 23
1-Butyl-3-(trifluoromethyl)-1H-pyrazole, **9a** 24
8-(4-(Trifluoromethyl)phenyl)-1,4-dioxa-8-azaspiro[4.5]decane, **11a** 25
tert-Butyl 4-(4-(trifluoromethyl)phenyl)piperazine-1-carboxylate, **12a** 26
2-Allyl-4-fluoro-1-(trifluoromethyl)benzene, **17a** 27
5-Methoxy-1,3-dimethyl-2-(trifluoromethyl)-1H-indole, ... 28
N-(4-(trifluoromethyl)benzyl)prop-2-en-1-amine, **18a** 29
Diethyl 4-(trifluoromethyl)isophthalate, **25a** 30
Methyl-(R)-2-((tert-butoxycarbonyl)amino)-3-(4-(trifluoromethyl)phenyl)propanoate, **28a** 31
Ethyl-(R)-2-((tert-butoxycarbonyl)amino)-3-(4-(trifluoromethyl)phenyl)propanoate, **29a** 32
(1R,5S)-3-Butyl-1-(4-(trifluoromethyl)phenyl)-3-azabicyclo[3.1.0]hexane, **31a** 33
(3aR,5R,6S,6aR)-5-((R)-2,2-dimethyl-1,3-dioxolan-4-yl)-2,2-dimethyltetrahydrofuro[2,3-d][1,3]dioxol-6-yl 4-(trifluoromethyl)benzoate, **32a** 34
4-(Trifluoromethyl)-1,1'-biphenyl, **34a** 35
General Hydrodefluorination Procedures 36
Electrochemical set-up 38
Notes on setting up electrochemical hydrodefluorination reactions: 40
Characterisation of Ar-CF_2H Products 41
4-Fluoro-(difluoromethyl)benzene, **1b** 41
(Difluoromethyl)benzene, **2b** 42
N-(4-(difluoromethyl)phenyl)acetamide, **3b** 43
4'-(Difluoromethyl)-2,6-dimethyl-1,1'-biphenyl, **4b** 44
Ethyl 4-(difluoromethyl)benzoate, **5b** 45
1-(Difluoromethyl)-4-methoxybenzene, **6b** 46
10-(4-(Difluoromethyl)phenyl)-10H-phenothiazine,**7b** 47

2-(Difluoromethyl)-1,3,5-trimethoxybenzene, **8b** 48

2,4,6-Trimethoxybenzaldehyde, **8e** 48

1-Butyl-3-(difluoromethyl)-1H-pyrazole, **9b** 49

4-(Difluoromethyl)-N,N-dimethylaniline, **10b** 50

4-(Dimethylamino)benzaldehyde, **10e** 50

8-(4-(Difluoromethyl)phenyl)-1,4-dioxa-8-azaspiro[4.5]decane, **11b** 51

Tert-butyl 4-(4-(difluoromethyl)phenyl)piperazine-1-carboxylate, **12b** 52

1-(Benzyloxy)-3-(difluoromethyl)benzene, **13b** 53

1-(Difluoromethyl)-3-methoxybenzene, **14b** 54

Diethyl (4-(difluoromethyl)benzyl)phosphonate, **15b** 55

1-Allyl-4-(difluoromethyl)benzene, **16b** 56

2-Allyl-1-(difluoromethyl)-4-fluorobenzene, **17b** 57

N-(4-(difluoromethyl)benzyl)prop-2-en-1-amine, **18b** 58

1-(Difluoromethyl)-2-methylbenzene, **19b** 59

(4-(Difluoromethyl)phenyl)methanol, **20b** 60

(3-(Difluoromethyl)phenyl)methanamine, **21b** 61

2-(4-(Difluoromethyl)phenyl)-4,4,5,5-tetramethyl-1,3,2-dioxaborolane, **22b** 62

4-(Difluoromethyl)benzenesulfonamide, **23b** 63

Methyl 4-(difluoromethyl)benzoate, **24b** 64

Diethyl 4-(difluoromethyl)isophthalate, **25b** 65

2-(Difluoromethyl)-5-methoxy-1,3-dimethyl-1H-indole, **26b** 66

3-(4-(Difluoromethyl)phenoxy)-N-methyl-3-phenylpropan-1-amine, **27b** 67

Methyl-(R)-2-((tert-butoxycarbonyl)amino)-3-(4-(difluoromethyl)phenyl)propanoate, **28b** 68

Ethyl-(R)-2-((tert-butoxycarbonyl)amino)-3-(4-(difluoromethyl)phenyl)propanoate, **29b** 69

(S)-3-(3-(Difluoromethyl)phenyl)-N-(1-(naphthalen-1-yl)ethyl)propan-1-amine, **30b** 70

(1R,5S)-3-Butyl-1-(4-(diifluoromethyl)phenyl)-3-azabicyclo[3.1.0]hexane, **31b** 71

(3aR,5R,6S,6aR)-5-((R)-2,2-Dimethyl-1,3-dioxolan-4-yl)-2,2-dimethyltetrahydrofuro[2,3-d][1,3]dioxol-6-yl 4-(difluoromethyl)benzoate, **32b** 72

3-(2-(Difluoromethyl)-10H-phenothiazin-10-yl)-N,N-dimethylpropan-1-amine, **33b** 73

Characterisation of Ar-CFH_2 products 74

1-Fluoro-4-(fluoromethyl)benzene, **1c** 74

(Fluoromethyl)benzene), **2c** 75

4'-(Fluoromethyl)-2,6-dimethyl-1,1'-biphenyl, **4c** 76

Ethyl 3-(fluoromethyl)benzoate **5c** 77

(1-(Fluoromethyl)-4-methoxybenzene), **6c** 78

1-(Benzyloxy)-3-(fluoromethyl)benzene, **13c** 79

(R)-3-(3-(Fluoromethyl)phenyl)-N-(1-(naphthalen-1-yl)ethyl)propan-1-amine, **30c** 80
1,2:5,6-Di-O-isopropylidene-3-O-[4-(fluoromethyl)benzoyl]-α-D-glucofuranose, **32c** 81
3-(2-(Fluoromethyl)-10H-phenothiazin-10-yl)-N,N-dimethylpropan-1-amine, **33c** 82
4-(Fluoromethyl)-1,1'-biphenyl, **34c** 83
Low-yielding and unsuccessful substrates 84
Mechanistic experiments 85
Product Hammett Analysis 86
Ar-CF_2H Hydrogen Bonding strength examination 89
NMR Spectra of Novel Substrates 90
1-Butyl-3-(trifluoromethyl)-1H-pyrazole, **9a** 90
2-Allyl-4-fluoro-1-(trifluoromethyl)benzene, **17a** 92
Diethyl 4-(trifluoromethyl)isophthalate, **25b** 94
5-Methoxy-1,3-dimethyl-2-(trifluoromethyl)-1H-indole, **26a** 96
Ethyl-(R)-2-((tert-butoxycarbonyl)amino)-3-(4-(trifluoromethyl)phenyl)propanoate, **29a** 98
(1R,5S)-3-Butyl-1-(4-(trifluoromethyl)phenyl)-3-azabicyclo[3.1.0]hexane, **31a** 100
NMR Spectra of Novel Ar-CF_2H Products 102
4'-(Difluoromethyl)-2,6-dimethyl-1,1'-biphenyl, **4b** 102
10-(4-(Difluoromethyl)phenyl)-10H-phenothiazine, **7b** 104
1-Butyl-3-(difluoromethyl)-1H-pyrazole, **9b** 106
8-(4-(Difluoromethyl)phenyl)-1,4-dioxa-8-azaspiro[4.5]decane, **11b** 108
Tert-butyl 4-(4-(difluoromethyl)phenyl)piperazine-1-carboxylate, **12b** 110
Diethyl (4-(difluoromethyl)benzyl)phosphonate, **15b** 112
1-Allyl-4-(difluoromethyl)benzene, **16b** 114
2-Allyl-1-(difluoromethyl)-4-fluorobenzene, **17b** 116
N-(4-(Difluoromethyl)benzyl)prop-2-en-1-amine, **18b** 118
Diethyl 4-(difluoromethyl)isophthalate, **25b** 120
2-(Difluoromethyl)-5-methoxy-1,3-dimethyl-1H-indole, **26b** 122
3-(4-(Difluoromethyl)phenoxy)-N-methyl-3-phenylpropan-1-amine, **27b** 124
Ethyl-(R)-2-((tert-butoxycarbonyl)amino)-3-(4-(difluoromethyl)phenyl)propanoate, **29b** 126
(S)-3-(3-(Difluoromethyl)phenyl)-N-(1-(naphthalen-1-yl)ethyl)propan-1-amine, **30b** 128
(1R,5S)-3-Butyl-1-(4-(diifluoromethyl)phenyl)-3-azabicyclo[3.1.0]hexane, **31b** 130
NMR Spectra of Novel Ar-CFH_2 Products 132
4'-(Fluoromethyl)-2,6-dimethyl-1,1'-biphenyl, **4c** 132
(R)-3-(3-(Fluoromethyl)phenyl)-N-(1-(naphthalen-1-yl)ethyl)propan-1-amine, **30c** 134
3-(2-(Fluoromethyl)-10H-phenothiazin-10-yl)-N,N-dimethylpropan-1-amine, **33c** 136
References 138

General Experimental Details

Techniques

Manipulations involving air and moisture sensitive materials were conducted employing standard Schlenk-line and glovebox techniques, using vacuum lines attached to a double manifold with greaseless J. Youngs valves equipped with an oil pump (0.1 mmHg) under an atmosphere of dry nitrogen. All glassware was dried overnight before use, in a 180 ºC oven and then allowed to cool under vacuum at 0.05 mbar. The removal of solvents in vacuo was achieved using a Büchi rotary evaporator (bath temperatures up to 40 °C) at a pressure of 15 mmHg (diaphragm pump), or at 0.05 mbar (oil pump) on a vacuum line at room temperature. The addition of < 200 µL of liquids was *via* a Gilson PIPETMAN p20, for larger volumes standard syringe practices were employed.

Solvents

THF (tetrahydrofuran), CH_2Cl_2, CH_3CN and Et_2O was dried using an Anhydrous Engineering alumina column drying system situated in the University of Bristol's chemistry department. All solvents were collected using Strauss flasks using a gastight J. Youngs valve. CH_3CN was degassed by four freeze-pump-thaw cycles under N_2. Deuterated solvents for NMR analysis were purchased from Sigma Aldrich.

Chromatography

TLC analysis was performed on Merck Silica gel $60F_{254}$ glass backed plates. Visualisation was achieved by UV fluorescence (254 nm) or staining with basic $KMnO_4$ or PMA. Flash column chromatography was conducted using Merck 60 silica: 230-400 mesh (40-63 µm) or using a Biotage Selekt automated flash purification system using Biotage Sfar HC Duo pre-packed columns of size 5 g, 25 g or 50 g.

Reagents

All reagents were purchased from TCI UK, Apollo Scientific, Sigma Aldrich, Alfa Aeser or Fluorochem and used as received unless otherwise stated. Anhydrous TMSCl was purchased from Sigma Aldrich and stored under N_2 over 3Å molecular sieves. Electrolyte salts ($TEAPF_6$, $TBAPF_6$, TBAB) were purchased from Sigma Aldrich and stored in a vacuum desiccator between uses.

LCMS

LCMS analysis was performed on an Agilent Infinity 1260 series instrument equipped with a single quadrupole mass spectrometer with either ESI or APCI ion source using MeCN + 0.1% Formic Acid or H_2O, 0.1% Formic Acid or a combination thereof as mobile phases. A Xterra MS C_{18} (3.5 µM, 3.0 x 20 mm IS) column was used for routine analysis alongside a VWD set to 220 nM.

Method 1: Column temperature 40ºC, flow rate 0.8 mL min^{-1}. A = MeCN + 0.1% Formic Acid, B = H_2O + 0.1% Formic Acid. Gradient elution: t = 0 min (95:5, A:B), t = 5 min (5:95, A:B), t = 7 min (5:95, A:B), t = 7.1 min (95:5 A:B), t = 8 min (95:5, A:B). Total run time 8 minutes.

Method 2: Column temperature 40ºC, flow rate 0.8 mL min^{-1}. A = MeCN + 0.1% Formic Acid, B = H_2O + 0.1% Formic Acid. Gradient elution: t = 0 min (95:5, A:B), t = 5 min (50:95, A:B), t = 7 min (50:95, A:B), t = 7.1 min (95:5 A:B), t = 8 min (95:5, A:B). Total run time 8 minutes.

Analysis

NMR spectra were recorded on Bruker Nano 400 or Bruker Advance III HD 500 cryo spectrometers. Chemical shifts (δ) are quoted in parts per million (ppm), referenced to the residual solvent peak (^{1}H and ^{13}C NMR) and coupling constants (J) are given in Hz. Multiplicities are abbreviated as: s (singlet), d (doublet), t (triplet), q (quartet), m (multiplet) or combinations thereof. NMR shifts for novel compounds have been assigned with the use of the appropriate 2D NMR experiments, such as COSY, HSQC and HMBC. Infrared spectra were recorded using a Perkin Elmer Spectrum Two FTIR spectrometer.

Preparative HPLC

HPLC purification was performed on a BUCHI C-850 FlashPrep fitted with a PrepPure C18 100 Å 10 μM, 250 x 20mm column using MeCN + 0.1% Formic Acid or H_2O, 0.1% Formic Acid or a combination thereof as mobile phases. Samples were liquid loaded in methanol (volume 1.0 – 5.0 mL) and a DIAD was employed with monitoring at 220 nM alongside an ELSD.

Method: Flow rate 25 mL min^{-1}. A = MeCN + 0.1% Formic Acid, B = H_2O + 0.1% Formic Acid. Gradient elution: t = 0 min (20:80, A:B), t = 15 min (60:40, A:B), t = 20 min (60:40, A:B).Total run time 30 minutes.

Electrochemical techniques

All cyclic voltametric (CV) and chronopotentiometry experiments were performed at room temperature using either an Autolab M101, MultiPalmsens 4 or ElectraSyn 2.0. CV experiments were carried out with a working electrode (GC = glassy carbon, Pt, Au, Ni = 1-3 mm diameter), a counter electrode (platinum wire) and a 0.1 M $Ag/AgNO_3$ reference electrode. All working electrodes were polished before each experiment. Before each CV, the solution was stirred for approximately 10 seconds, whilst being degassed by a stream of N_2. The CV cell was maintained under an atmosphere of N_2 during analysis.

High resolution mass spectra (HRMS)

HRMS were performed on a Bruker Daltonics MicrOTOF II by ESI, a Thermo Scientific QExactive by EI or a Thermo Scientific Orbitrap Elite by ESI or Atmospheric Pressure Chemical Ionisation (APCI).

Compound naming

Compound names were generated by ChemDraw Professional 20.0 (PerkinElmer) following IUPAC nomenclature.

Prevalence of $ArCF_2H$ in pharmaceutically-relevant compounds

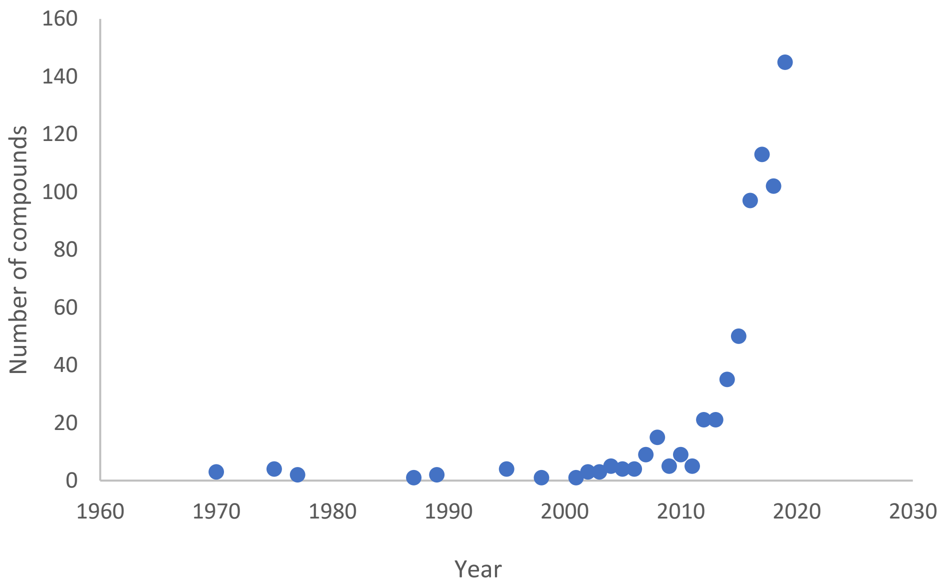

A search was made on Reaxys in Nov 2022 on the number of compounds reported in J. Med. Chem that contain the $ArCF_2H$ moiety between 1970 to 2019 (pre-pandemic). The results are displayed above.

Extended Optimisation Table

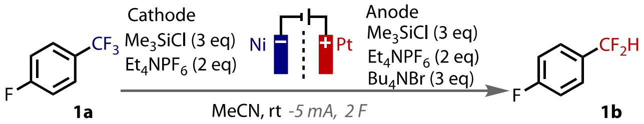

Entry	*Variation from optimised conditions*	*Yield CF_2H / %*	*CF_2H:CFH_2*
**1**	**None**	**76**	**25:1**
2	1 *F* not 2 *F*	47	>25:1
3	-5 mA pulsed voltammetry (30 s, 10 off)	traces	-
4	-10 mA not -5 mA	68	9:1
5	DCE not MeCN	*n.d*	-
6	Me-THF not MeCN	*n.d*	
7	DMF not MeCN	*n.d*	-
8	Me_3PhNPF_6 not Et_4NPF_6	<5	-
9	Bu_4NClO_4 not Et_4NPF_6	71	20:1
10	KPF_6 not Et_4NPF_6	*n.d*	-
11	+ Ni foil not Pt	50	10:1
12	- Pt not Ni foil	72	5:1
13	- C not Ni foil	20	3:1
14	Bu_4NCl not Bu_4NBr	71	9:1
15	Using commercial powerpack	70	10:1
16	No TMSCl	64	7:1
17	$SiCl_4$ not TMSCl	40	5:1
18	HMDS not TMSCl	52	4:1
19	B_2Pin_2 not TMSCl	71	19:1
20	Undivided cell	n.d	-

Table S1. ^{19}F NMR yields relative to internal C_6F_6 standard. Ratios determined from crude ^{19}F and ^{1}H NMR spectra. Conducted on 0.5 mmol scale.

Role of TMSCl

The use of TMSCl was found to improve the selectivity for mono-hydrodefluorination as well as a trap for expelled fluoride (TMSF, b.p 16°C, observed in crude ^{19}F NMR spectra as soluble in MeCN). For substrates with nucleophilic functional groups (*e.g.* benzylamine **21a**) minimal conversion was observed in the presence of TMSCl due to competing reduction of HCl and N-TMS adducts – in these cases no TMSCl was found to give optimal yields (see main text). The bulkier silane, TESCl, was found to improve conversion, however two Ar-CF_2H containing products were formed, with the second putatively assigned as the N-TES adduct (overlapping NMR signals).

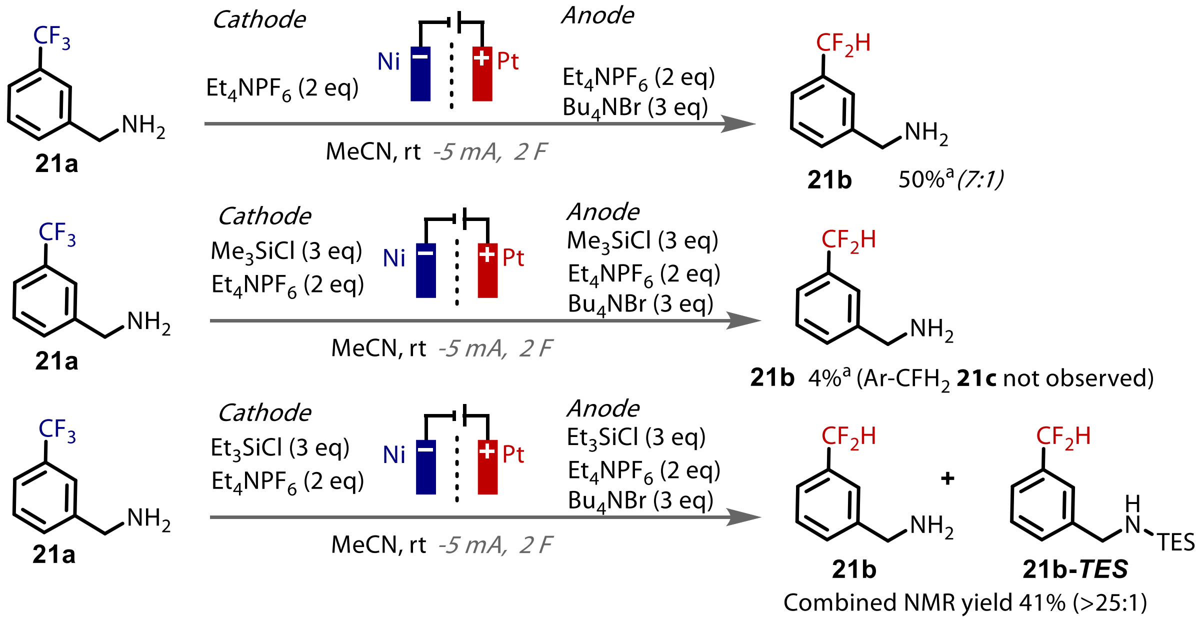

1. Results of **21a** reduction without TMSCl, with TMSCl and with TESCl. Putatively assigned products for TESCl reaction given. [a] ^{19}F NMR yield. Selectivity of $ArCF_2H$:$ArCFH_2$ given in brackets determined from crude ^{19}F NMR.

The reduction potential of TMSCl has been reported as -3.1 V vs SCE (L. Lu, J. C. Siu, Y. Lai, S. Lin, *J. Am. Chem. Soc.* **2020**, *142*, 21272–21278) and as such we do not believe it to be reduced under the reaction conditions as potentials that negative have not been applied.

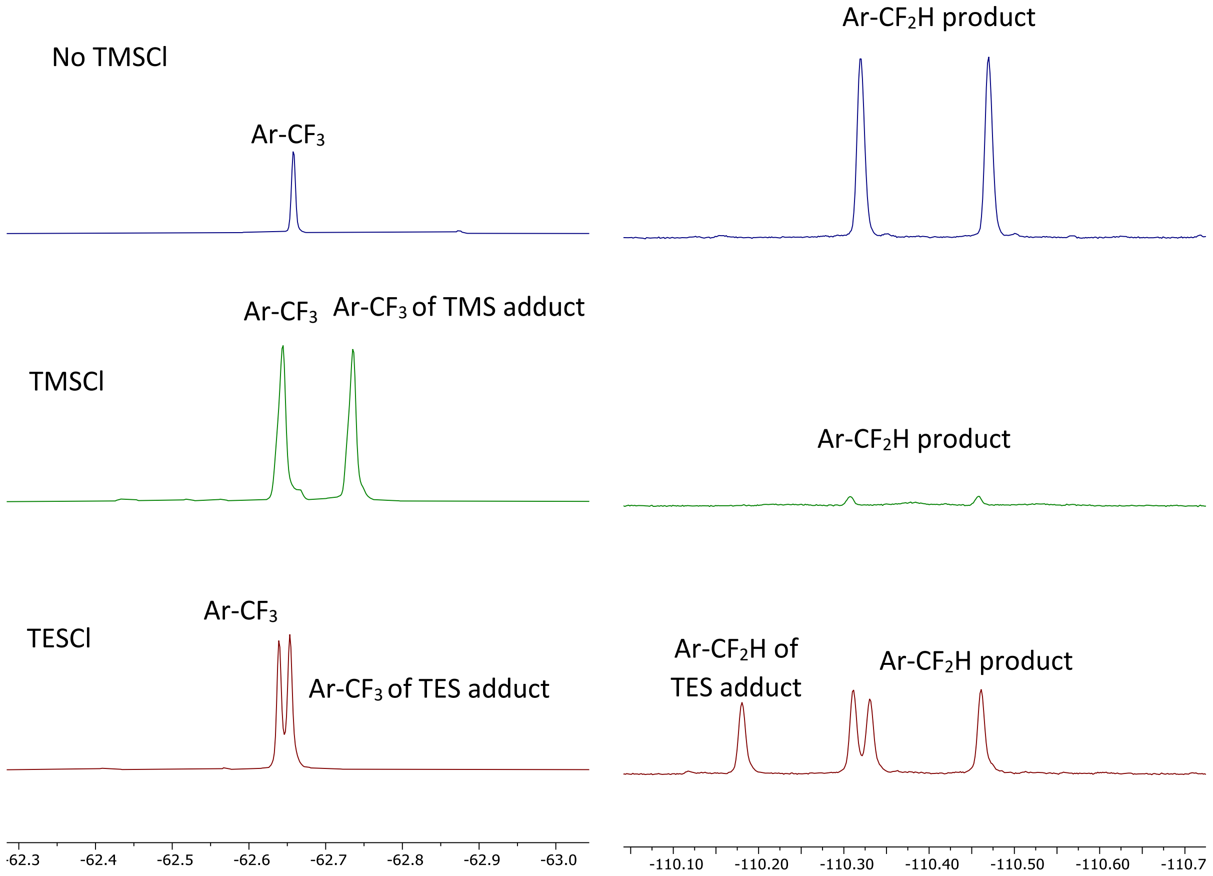

Figure S2. Stacked ^{19}F NMR spectra for reduction of **21a** with a) No TMSCl (top), b) TMSCl (middle) and c) TESCl (bottom).

The formation of adducts that are more easily reduced than the **21a** is evident from the reaction E_{cell} (Figure S3). A more negative potential is required for the reduction of **21a** without TMSCl, however the potential is consistent until almost complete conversion. Dramatic drops in potential are observed with TESCl at 1 *F*, likely due to the consumption of 1 equivalent of N-TES adduct, after which a potential sufficiently negative to facilitate hydrodefluorination is applied.

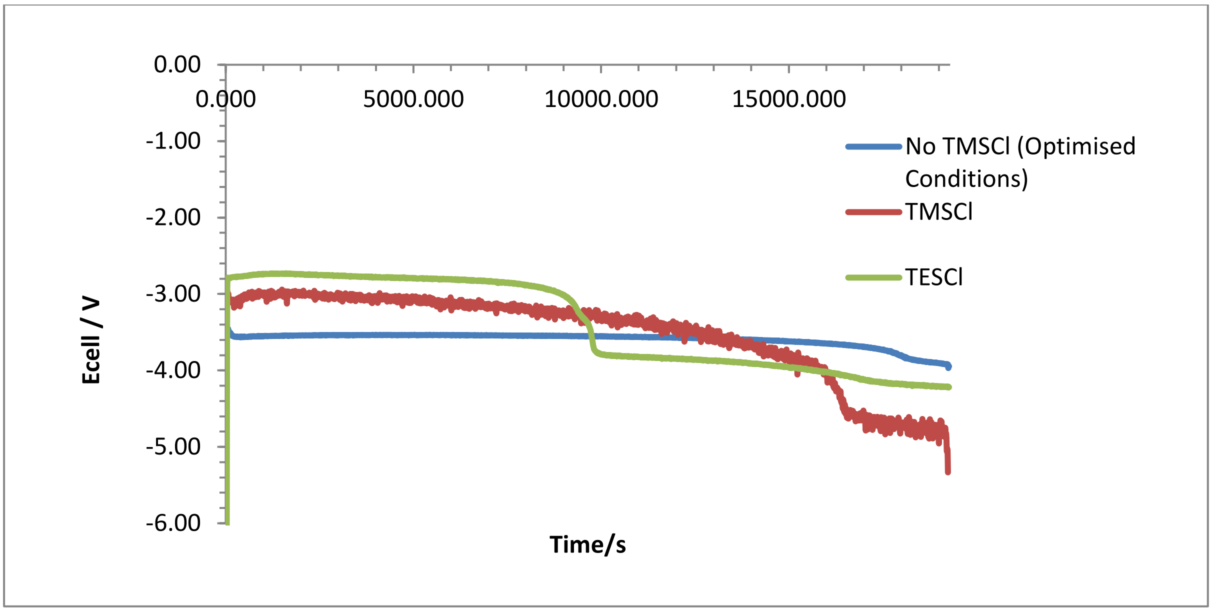

Figure S3. Plot of E_{cell} vs time for hydrodefluorination reactions of **21a** with and without TMSCl and with TESCl.

The addition of TBDMSCl, TESCl or TIPSCl to a solution of **21a** in MeCN resulted in the immediate formation of an insoluble white precipitate, indicating that the issue would likely persist even with silanes with increased steric bulk than TESCl. A lower reaction $|E_{cell}|$ is also observed in the presence of TMSCl, perhaps due to Lewis acidic effects. This lower $|E_{cell}|$ likely contributes to improved selectivity through minimising overreduction.

CV and SWV Studies

Cyclic voltammograms

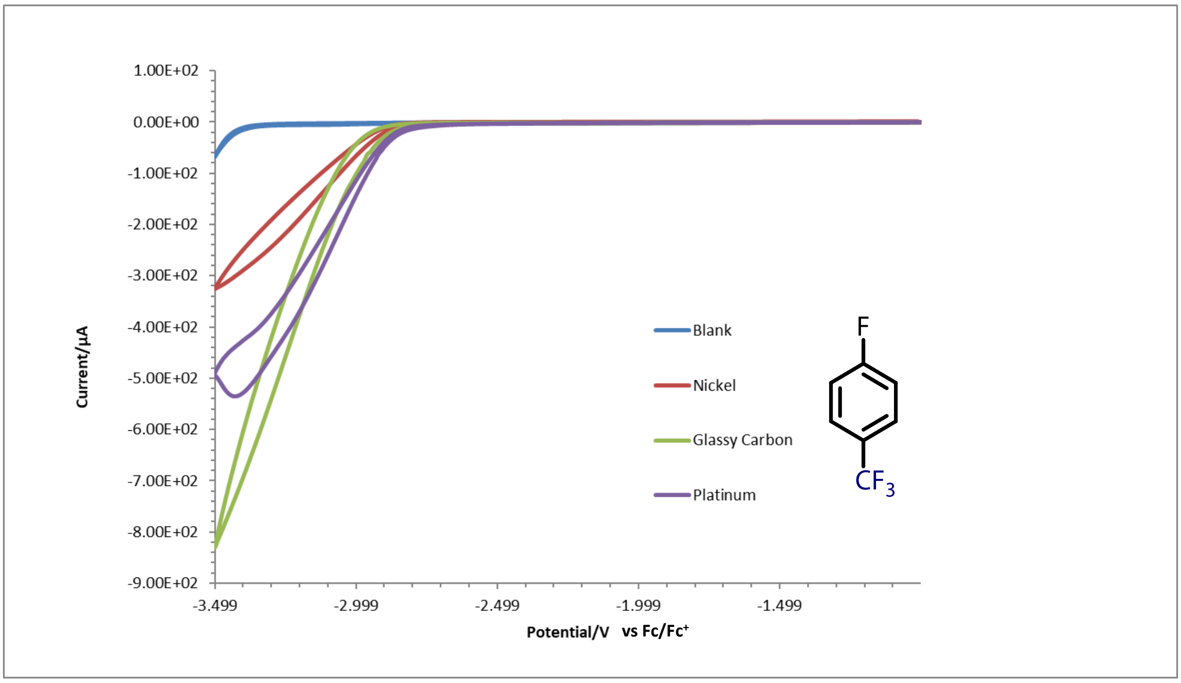

Figure S4. CVs of model substrate **1a**. Cathode: as indicated (disk): Anode: Pt(coil), 0.1 M $TBAPF_6$, 5 mM substrate, 2.5 mL degassed DMF under N_2, scan rate = 100 mv/s, $Ag/AgNO_3$ (0.1 M) reference electrode, externally referenced to Fc/Fc^+.

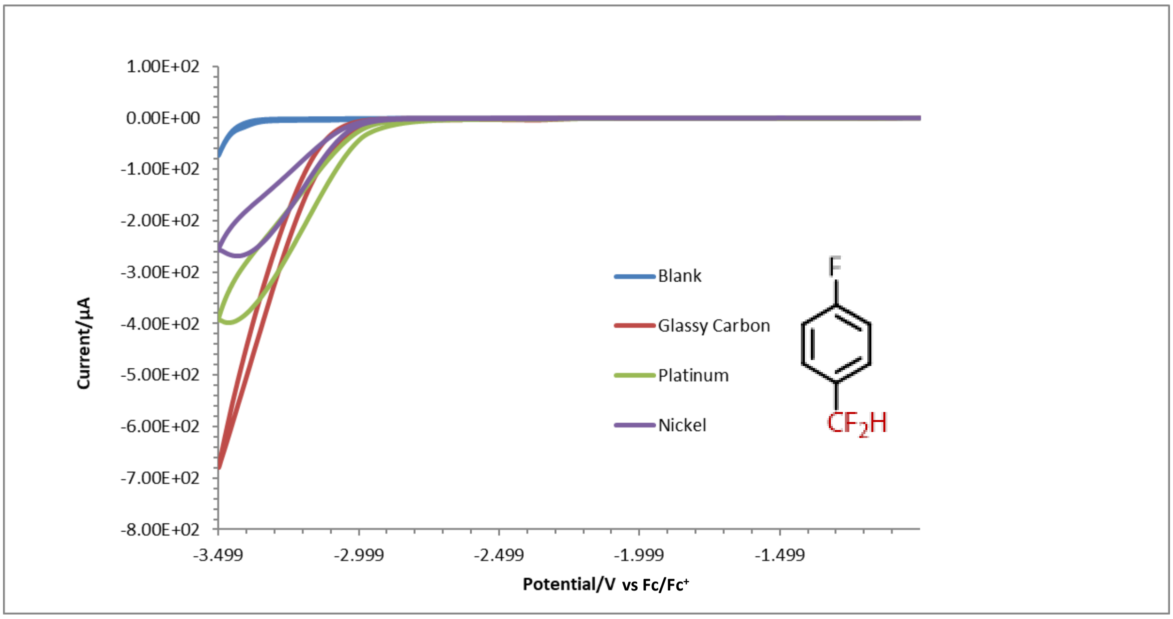

Figure S5. CVs of **1b**. Cathode: as Indicated (disk): Anode: Pt(coil), 0.1 M $TBAPF_6$, 5 mM substrate, 2.5 mL degassed DMF under N_2, scan rate = 100 mv/s, $Ag/AgNO_3$ (0.1 M) reference electrode, externally referenced to Fc/Fc^+.

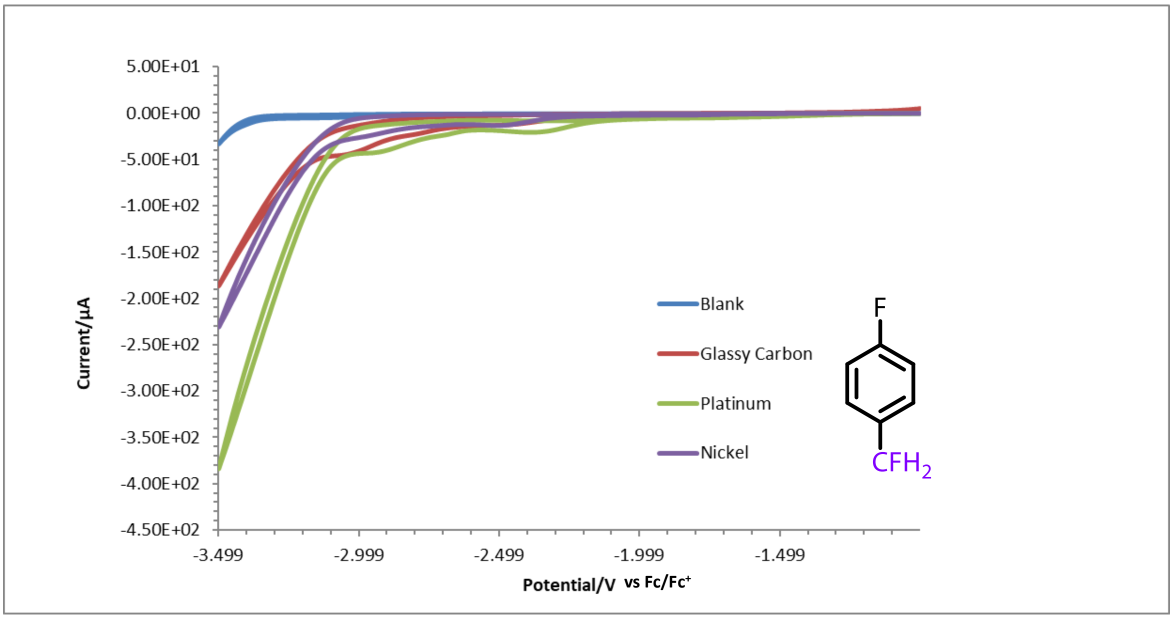

Figure S6. CVs of **1c**. Cathode: as Indicated (disk): Anode: Pt(coil), 0.1 M $TBAPF_6$, 5 mM substrate, 2.5 mL degassed DMF under N_2, scan rate = 100 mv/s, Ag/$AgNO_3$ (0.1 M) reference electrode, externally referenced to Fc/Fc^+.

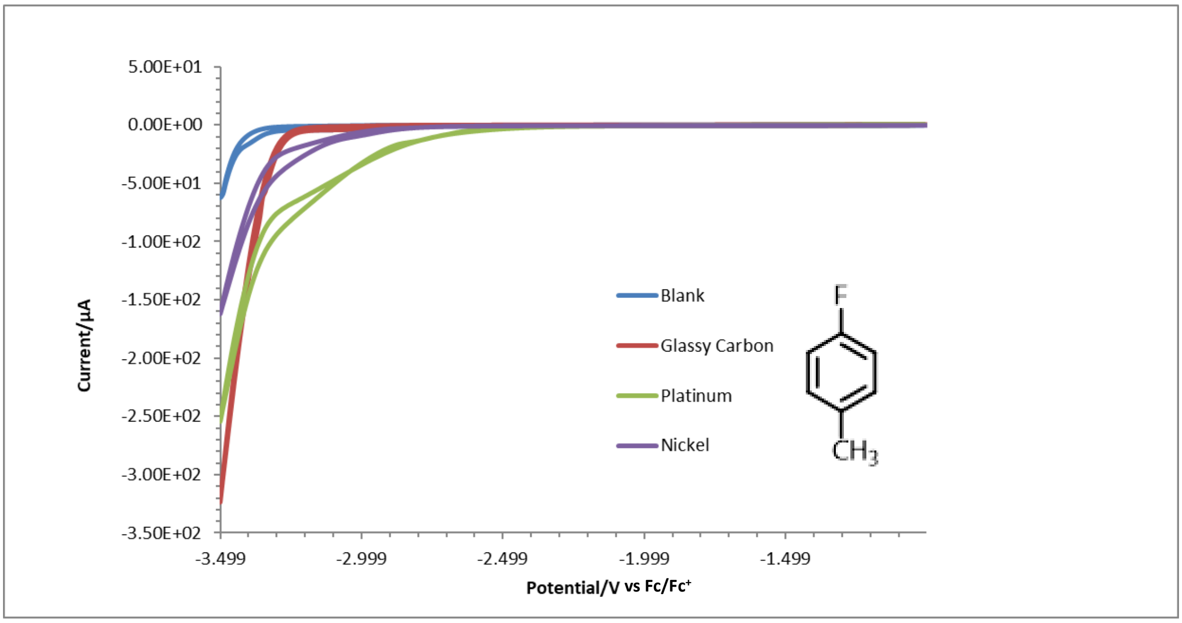

Figure S7. CVs of 4-fluorotoluene. Cathode: as Indicated (disk): Anode: Pt(coil), 0.1 M $TBAPF_6$, 5 mM substrate, 2.5 mL degassed DMF under N_2, scan rate = 100 mv/s, Ag/$AgNO_3$ (0.1 M) reference electrode, externally referenced to Fc/Fc^+.

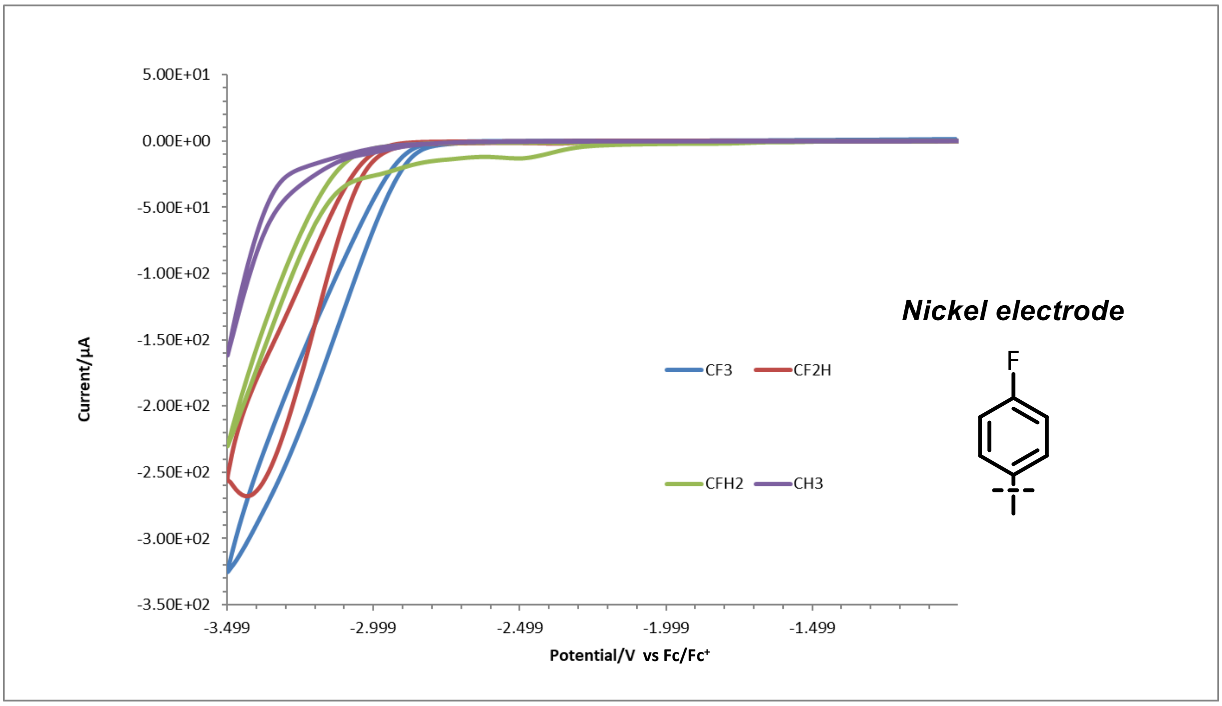

Figure S8. CVs of **1a-c** and 4-fluorotoluene. Cathode: Ni (disk): Anode: Pt(coil), 0.1 M $TBAPF_6$, 5 mM substrate, 2.5 mL degassed DMF under N_2, scan rate = 100 mv/s, Ag/$AgNO_3$ (0.1 M) reference electrode, externally referenced to Fc/Fc^+.

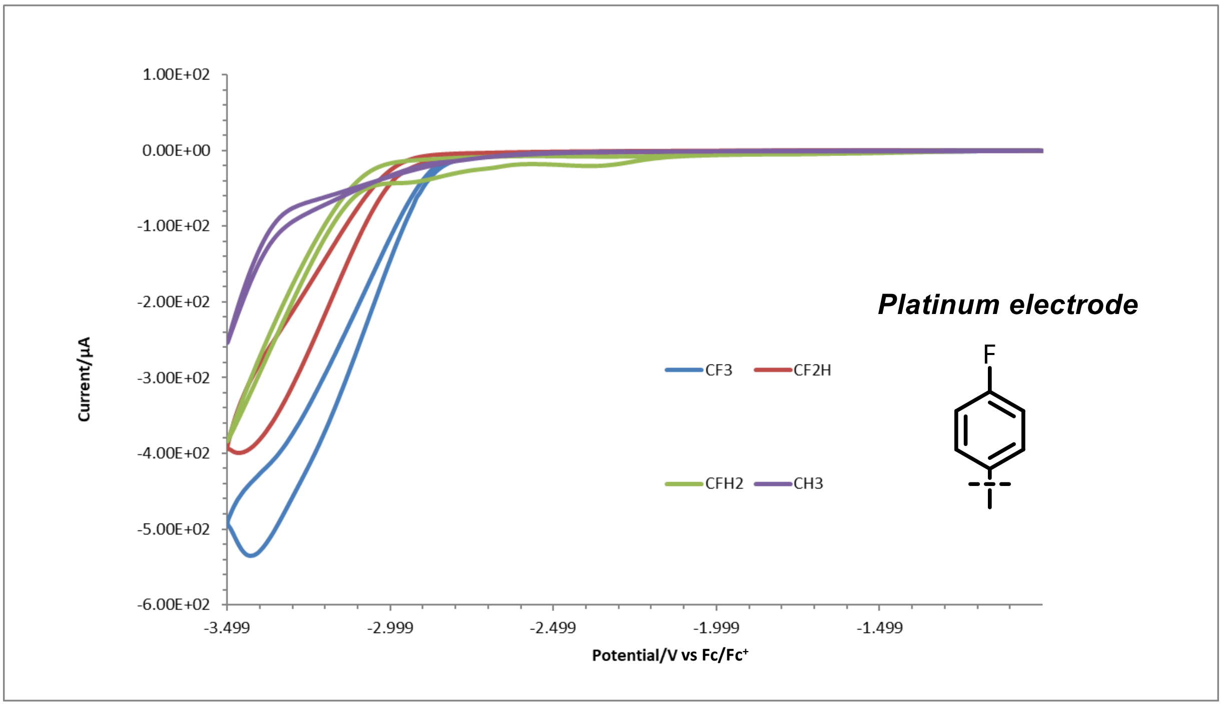

Figure S9. CVs of **1a-c** and 4-fluorotoluene. Cathode: Pt (disk): Anode: Pt(coil), 0.1 M $TBAPF_6$, 5 mM substrate, 2.5 mL degassed DMF under N_2, scan rate = 100 mv/s, Ag/$AgNO_3$ (0.1 M) reference electrode, externally referenced to Fc/Fc^+.

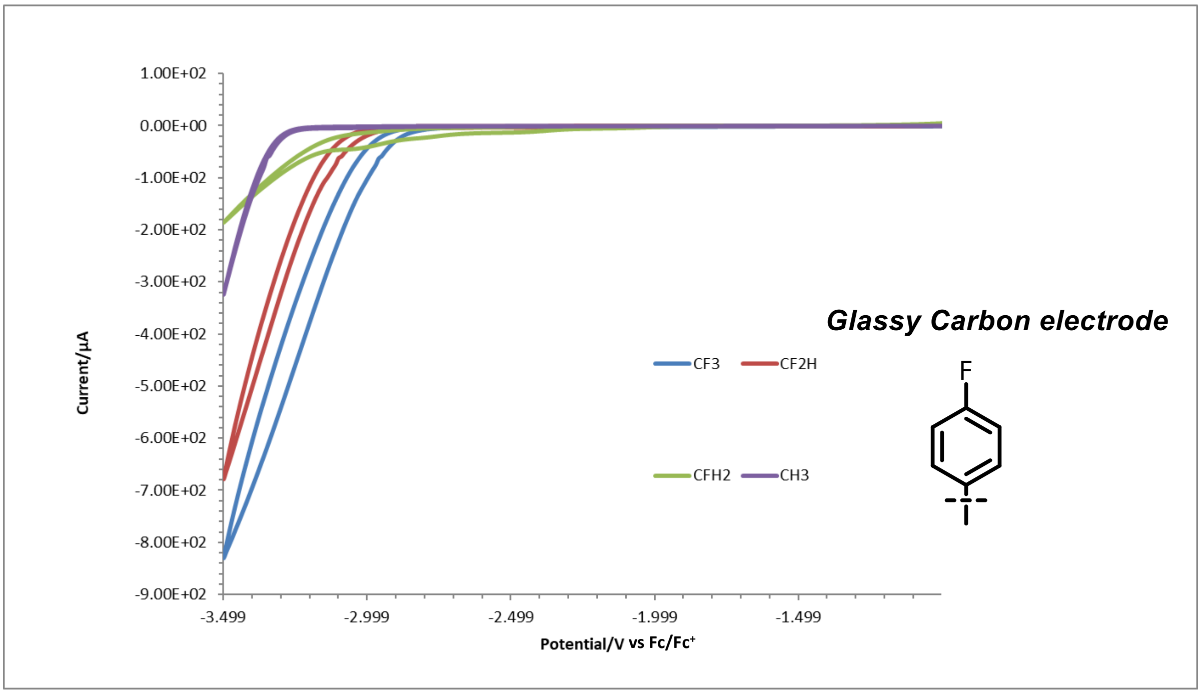

Figure S10. CVs of **1a-c** and 4-fluorotoluene. Cathode: GC (disk): Anode: Pt(coil), 0.1 M $TBAPF_6$, 5 mM substrate, 2.5 mL degassed DMF under N_2, scan rate = 100 mv/s, $Ag/AgNO_3$ (0.1 M) reference electrode, externally referenced to Fc/Fc^+.

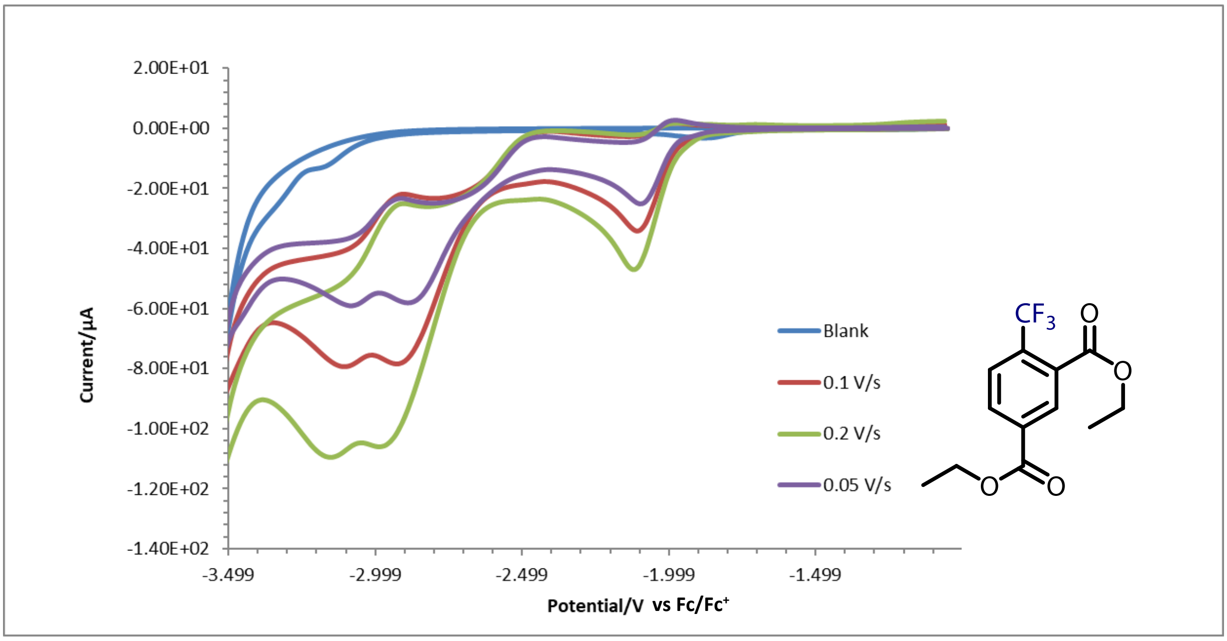

Figure S11. CVs of **25a**. Cathode: Ni (disk): Anode: Pt (coil), 0.1 M $TBAPF_6$, 5 mM substrate, 2.5 mL degassed DMF under N_2, scan rate = as indicated, $Ag/AgNO_3$ (0.1 M) reference electrode, externally referenced to Fc/Fc^+.

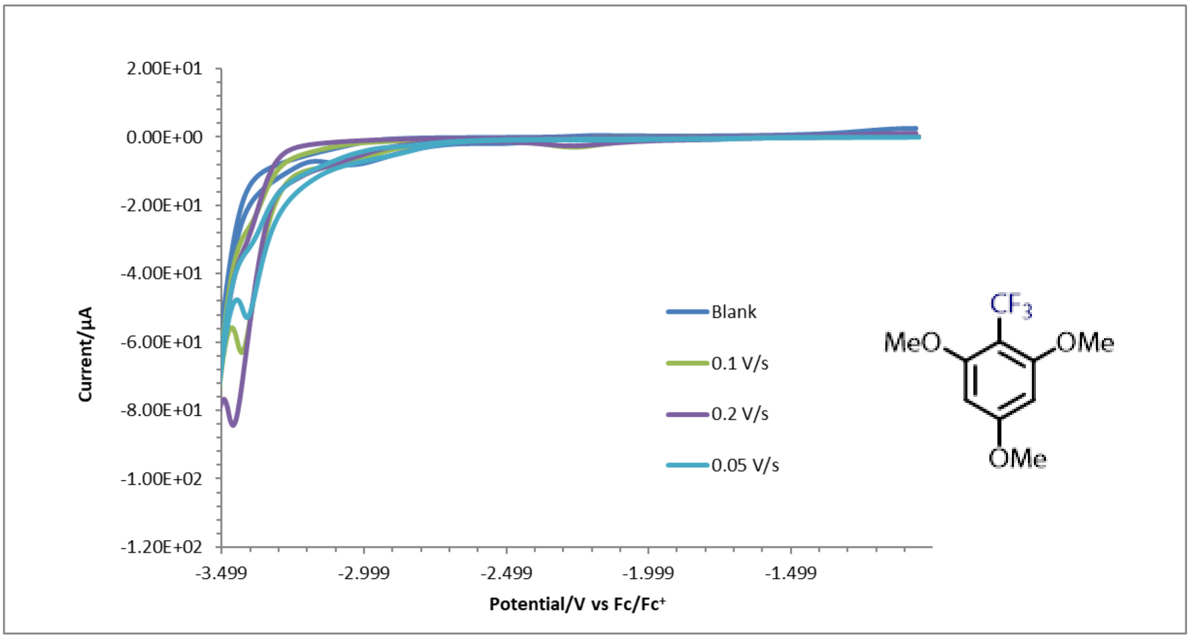

Figure S12. CVs of **8a**. Cathode: Ni (disk): Anode: Pt (coil), 0.1 M $TBAPF_6$, 5 mM substrate, 2.5 mL degassed DMF under N_2, scan rate = as indicated, Ag/$AgNO_3$ (0.1 M) reference electrode, externally referenced to Fc/Fc^+.

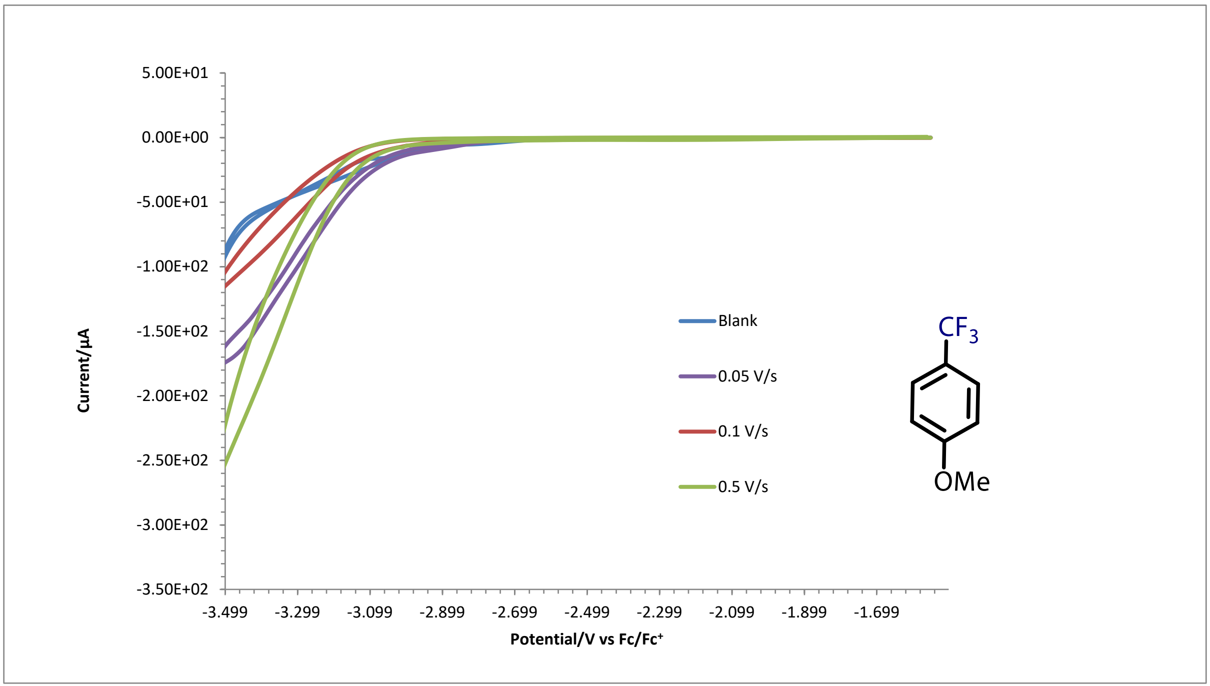

Figure S13. CVs of **6a**. Cathode: Ni (disk): Anode: Pt (coil), 0.1 M $TBAPF_6$, 5 mM substrate, 2.5 mL degassed DMF under N_2, scan rate = as indicated, Ag/$AgNO_3$ (0.1 M) reference electrode, externally referenced to Fc/Fc^+.

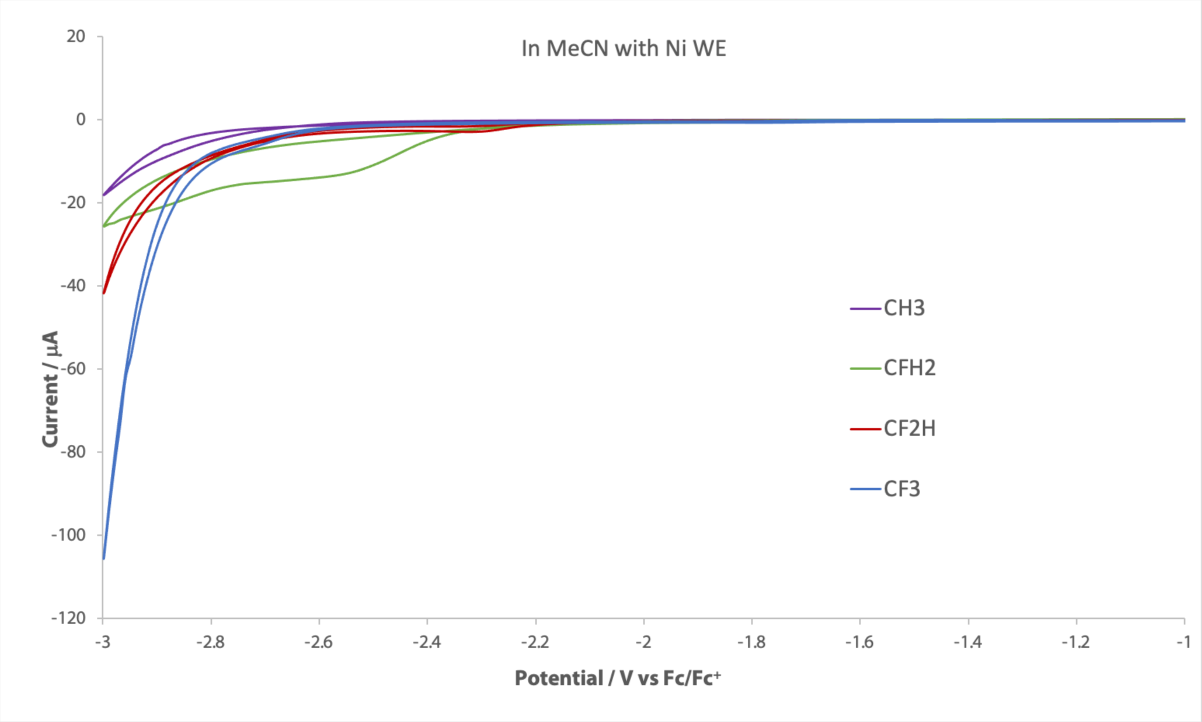

Figure S14. CVs of **1a-c** and 4-fluorotoluene in MeCN. Cathode: Ni (disk): Anode: Pt (coil), 0.1 M $TBAPF_6$, 5 mM substrate, 2.5 mL degassed MeCN under N_2, scan rate = 0.1 V/s, $Ag/AgNO_3$ (0.1 M) reference electrode, externally referenced to Fc/Fc^+.

Square wave voltammetry

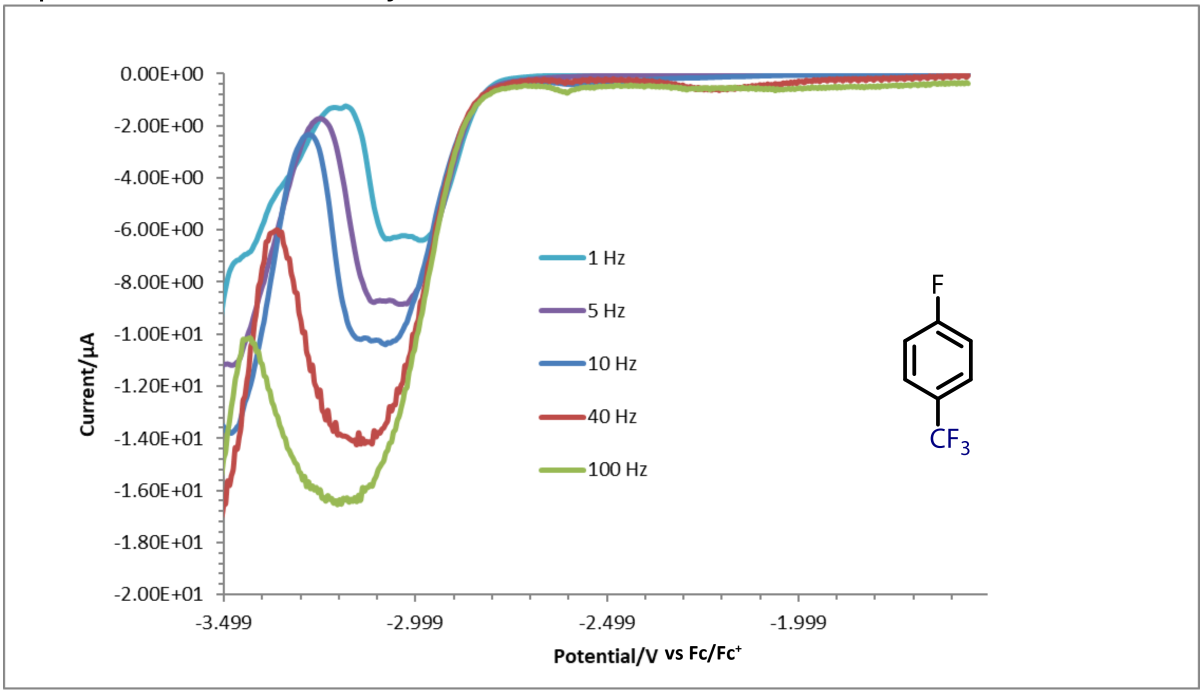

Figure S15. SWV of **1a**. Cathode: Ni (disk): Anode: Pt(coil), 0.1 M $TBAPF_6$, 5 mM substrate, 2.5 mL degassed DMF under N_2, E_{Step} = 0.005 V, Amplitude = 0.01 V, Frequency = As indicated, $Ag/AgNO_3$ (0.1 M) reference electrode, externally referenced to Fc/Fc^+.

Benchmarking details

To allow for a fair comparison between experiments, each of the 4 reported conditions that we tested were run alongside a substrate reported in the original literature to ensure the conditions used matched the reported yield before repeating on our substrates. In addition, for every batch of substrates tested, a substrate reported in the original literature was run alongside to ensure good reproducibility.

Shang (ref 36 in manuscript):[1]

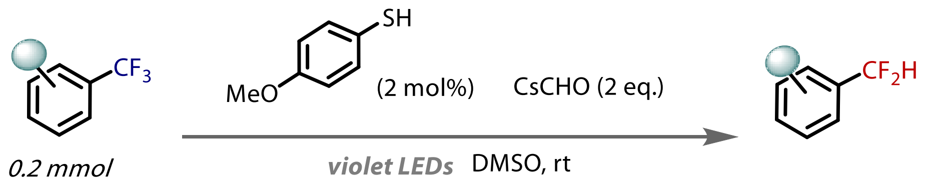

Scheme S1. Shang conditions used for benchmarking.

A Schlenk tube with a stir bar was oven dried and cooled under vacuum. After backfilling of N_2, cesium formate (71 mg, 0.40 mmol, 2.0 eq.) and the substrate (if solid) (0.2 mmol, 1 eq.) were loaded and purged-backfilled with N_2 5 times.

The substrate (if liquid) (0.2 mol, 1eq.), 4-methoxybenzenethiol (4.9 µL, 5.6 mg, 0.040 mmol, 0.20 eq.) were pipetted in under a positive pressure of N_2, and anhydrous DMSO (2mL), previously sparged for 30 min with N_2, was added via needle.

Under a positive pressure of N_2 the septum was replaced by a greased stopped, which was secured with a clip. The sealed vial was then exposed to irradiation by a violet light (KESSIL PR160-427 nm) from about 5 cm of the side, while being maintained at room temperature by the cooling of a fan for 24 h.

Derivation from literature: None

Jui (ref 34 in manuscript): [2]

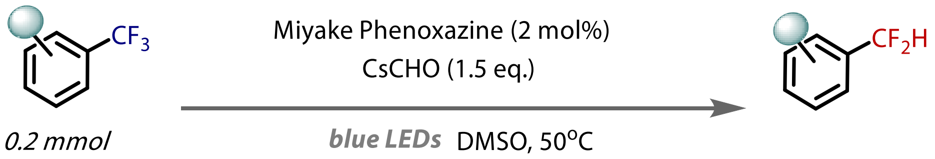

Scheme S2. Jui conditions used for benchmarking.

A Schlenk tube with a stir bar was oven dried and cooled under vacuum. After backfilling of N_2, caesium formate (270 mg, 1.5 mmol, 3.0 eq.) was loaded and purged-backfilled with N_2 5 times.

Another oven-dried Schlenk tube with a stir bar was cooled under vacuum and used to a stock solution of Miyake phenoxazine:
Miyake phenoxazine was added to the Schlenk tube (27 mg) then purged-backfilled with N_2 5 times. Anhydrous DMSO (17.1 mL, 0.0025 M), previously sparged for 30 min with N_2, was added via needle. This solution of the catalyst (4 mL, 0.01 mmol) was then transfer via syringe to the Schlenk tube contain the cesium formate.

The substrate (0.5 mol, 1eq.), was loaded in a 1 mL Eppendorf and dissolved in 1mL of additional DMSO (degassed as above) then added via syringe into the reaction mixture. Under a positive pressure of N_2 the septum was replaced by a greased stopped, which was secured with a clip. The sealed vial was then exposed to irradiation by a blue light (KESSIL) from above, while being stirred at 600 rpm in a 50 °C oil bath for 24 or 48 h.

Derivation from literature:

- Use of a stock solution

Owing to the small scale, it was challenging to accurately dose small amounts of solid catalyst under a positive pressure of nitrogen and so anhydrous stock solutions were used, with reaction concentration being maintained as reported in the original literature.

- Light source

The specified lamp type is no longer available and no emission spectrum reported. An alternative as close to the original report was used in place (Blue LED).

- Reaction time

Monitoring of the crude reaction mixture showed incomplete conversion after the time specified in the original report and so the reaction was continued until the NMR yield of the control run (of original substrate) was in line of that in the original work.

Gouverneur (ref 35 in manuscript):[3]

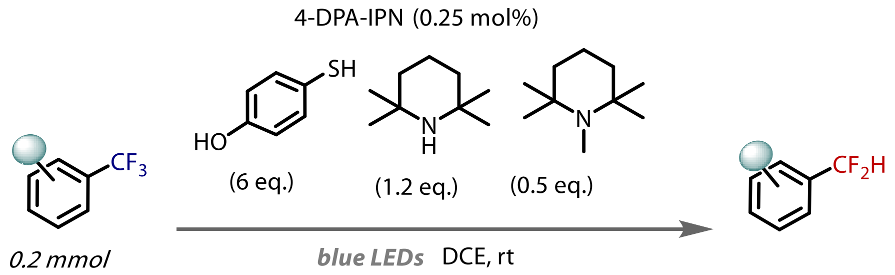

Scheme S3. Gouverneur conditions used for benchmarking.

In Eppendorf tubes, the following stock solutions were prepared in DCE:

TMP (82uL in 1mL DCE, 0.44 M), PMP (36 µL in 1mL DCE, 0.20 M), 4-HTP (241 µL in 1mL DCE, 1.9 M) (4-HTP was molten by gentle heating of the container in a small water bath prior to pipetting), 4-DPA-IPN (0.8 mg in 1mL DCE, 0.0012 M) and the substrate (0.1 mmol in 0.5 mL DCE, 0.2 M).

To an oven-dried and vacuum-cooled Schlenk tube with a stir bar, was added via syringe 2.5 mL of anhydrous DCE, then the stock solutions in the following amounts: TMP, 270 µL; PMP, 250 µL; 4-HTP, 310 µL; 4-PDA-IPN, 250 µL; substrate, 500uL. The resulting solution was sparged for 2 min with N_2, then under a positive pressure of N_2, a greased stopped was fitted and secured with a clip. The sealed vial was then exposed to irradiation by a blue light (KESSIL) from about 5 cm of the side, while being maintain at room temperature by the cooling of a fan for 20 h.

Derivation from literature:

- Light source

The specified lamp type is no longer available and no emission spectrum reported however a wavelength was given. An alternative as close to the original report with the same wavelength was used in place (Blue Kessil).

- Reaction time

Monitoring of the crude reaction mixture showed incomplete conversion after the time specified in the original report and so the reaction was continued until the NMR yield of the control run (of original substrate) was in line of that in the original work.

Typical reaction set-up:

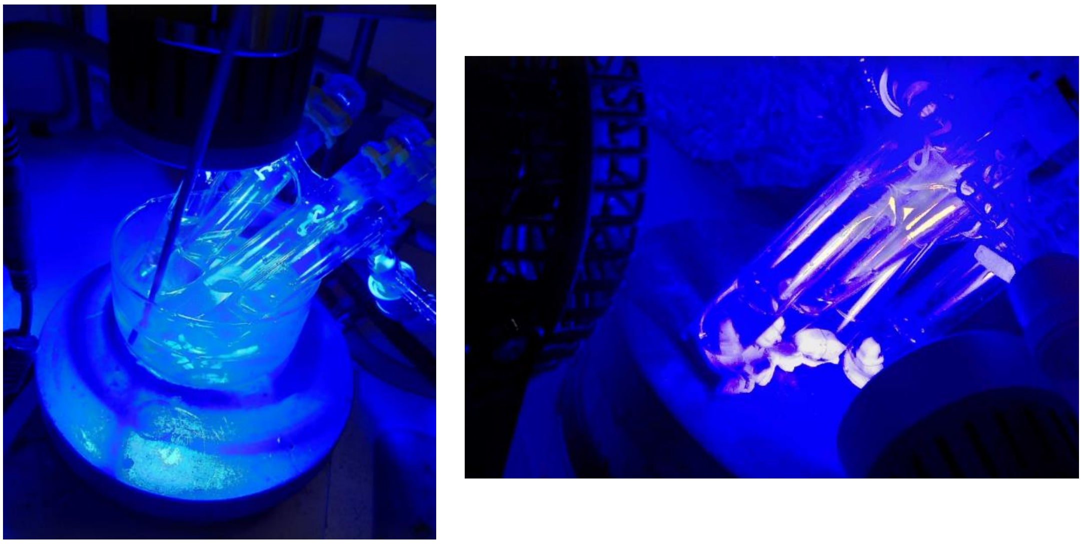

Figure S16. Typical reaction set up for photochemical benchmarking.

Prakash (ref 36 in manuscript):[4]

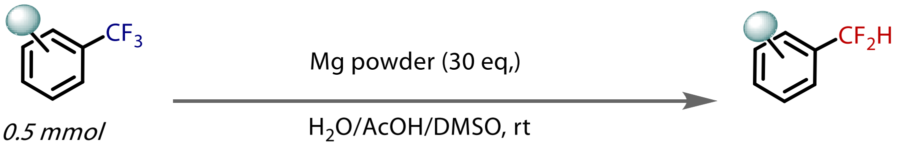

Scheme S4. Prakash conditions used for benchmarking.

To a stirred solution of substrate (1.0 mmol) in 8.0 mL of DMSO was added Mg^0 powder (30 equiv., 360 mg, –20+100 mesh) and 1.50 mL of a 2:1 (v/v) stock solution of glacial AcOH/H_2O in one portion. The resulting mixture was vigorously stirred (750 rpm) at room temperature overnight and the resulting mixture analysed by ^{19}F NMR after dilution with DMSO and agitation.

Owing to the very viscous non-homogenous solution, it was difficult to accurately determine an NMR yield against an internal standard and as such conversion is reported.

Derivation from literature: None

2,6-Dimethyl-4'-(trifluoromethyl)-1,1'-biphenyl, ***4a***

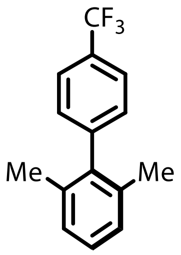

To a 20 mL screw-capped vial was added 2-bromo-1,3-dimethylbenzene (1.11 g, 3.00 mmol, 1 eq.), 4-trifluoromethylphenylboronic acid (1.25 g, 6.60 mmol, 1.10 eq.) and $Pd(PPh_3)_4$ (208 mg, 0.180 mmol, 0.0300 eq.). 1,2-Dimethoxyethane (7 mL) and 2 M aq. Na_2CO_3 (3 mL) was then added and the vial was capped and stirred at 100 °C overnight. The next day, the reaction mixture was filtered through celite eluting with chloroform and the solvent removed under reduced pressure. Flash column chromatography on silica gel (eluting with pentane) afforded **4a** as a colourless solid (860 mg, 57 %).

$\mathbf{R_f}$ = 0.7 (pentane)

^{1}H NMR (400 MHz, $CDCl_3$) δ: 7.76 – 7.68 (m, 2H), 7.34 – 7.28 (m, 2H), 7.23 (dd, *J* = 8.3, 6.7 Hz, 1H), 7.16 (d, *J* = 7.5 Hz, 2H), 2.05 (s, 6H).

^{13}C NMR (151 MHz, $CDCl_3$) δ: 144.9 (q, *J* = 1.4 Hz), 140.4, 135.7, 129.5, 129.0 (q, *J* = 32.4 Hz), 127.6, 127.5, 125.5 (q, *J* = 3.7 Hz), 124.1 (q, *J* = 271.97 Hz) 20.8.

^{19}F NMR (376 MHz, $CDCl_3$) δ: -62.4 (s, 3F).

Spectral data in accordance with literature.[5]

1,3,5-trimethoxy-2-(trifluoromethyl)benzene, ***8a***

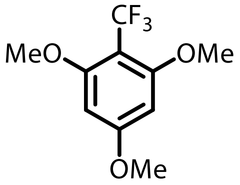

1,3,5-Trimethoxybenzene (10.1 g, 60.0 mmol, 1 eq.) was dissolved in MeCN (150 mL) and N-iodosuccinimide (15.0 g, 66 mmol, 1.1 eq.) was added portion-wise. The solution was stirred overnight at RT and then was diluted with 200 mL Et_2O and washed with sat. aq. $Na_2S_2O_3$ and 1 M aq. NaOH. The organic layer was dried with $MgSO_4$, filtered, and concentrated under reduced pressure to afford 1,3,5-trimethoxy-2-(iodo)benzene (16.0 g, 90%) as a colourless powder, which was used without purification in the next step.

1,3,5-trimethoxy-2-(iodo)benzene (2.94 g, 10.0 mmol, 1 eq.) and CuI (1.90 g, 10.0 mmol, 1.00 eq.) were added to an oven-dried, vacuum-cooled vial fitted with a rubber septum. DMF (50 mL) and methyl 2,2-difluoro-2-(fluorosulfonyl)acetate (1.53 mL, 2.30 g, 12.0 mmol, 1.20 eq.) was added and, under a positive pressure of N_2, the septum was sealed with a screw cap. The vial was heated to 120 °C overnight and the monitored until complete conversion observed by GCMS. The reaction mixture was filtered through celite eluting with Et_2O and the filtrate washed 5 times with brine, dried with $MgSO_4$, filtered and concentrated under reduced pressure. Flash column chromatography on silica gel (5-30% Et_2O in pentane) afforded **8a** as a colourless solid (0.970 g, 41 %).

R_F = 0.2 (5% Et_2O in pentane)

^{1}H NMR (400 MHz, $CDCl_3$) δ: 6.06 (s, 2H), 3.769 (overlap. s, 6H) 3.766 (overlap. s, 3H).

^{13}C NMR (151 MHz, $CDCl_3$) δ: 144.9 (d, *J* = 1.4 Hz), 140.4, 135.7, 129.5, 129.0 (q, *J* = 32.4 Hz), 127.6, 127.5, 125.5 (q, *J* = 3.7 Hz), 124.1 (q, *J* = 271.97 Hz) 20.8.

^{19}F NMR (376 MHz, $CDCl_3$) δ: -54.2 (s, 3F).

Spectral data in accordance with literature.[6]

1-Butyl-3-(trifluoromethyl)-1H-pyrazole, ***9a***

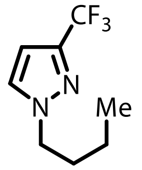

In a 20 mL vial, under air, a suspension of NaH (400 mg, 60% dispersion in mineral oil, 10.0 mmol, 1.20 eq.) in DMF (10 mL) was cooled to 0 °C. To this, 3-(trifluoromethyl)-1*H*-pyrazole (680 mg, 5.00 mmol, 1 eq.) was added portion wise (with care to avoid violent H_2 evolution) and then stirred for 15 minutes at 0 °C then warmed to RT over 45 minutes. BuBr (0.58 mL, 753 mg, 5.50 mmol, 1.1 equiv.) was added dropwise and the reaction was stirred overnight. The following day, the reaction was quenched with sat. aq. NH_4Cl and extracted with Et_2O. The organic layer was washed twice with brine, dried with $MgSO_4$, filtered, and eluted through a silica plug with Et_2O. Removal of the solvent under reduced pressure afforded **9a** as a yellow oil (370 mg, 39 %).

R_f = 0.3 (5% Et_2O in pentane)

^{1}H NMR (500 MHz, DMSO-*d*$_6$) δ: 7.97 (dd, *J* = 2.4, 1.1 Hz, 1H), 6.68 (dd, *J* = 1.7, 0.6 Hz, 1H), 4.19 (t, *J* = 7.1 Hz, 2H), 1.81 – 1.71 (m, 2H), 1.23 (pt, *J* = 7.6, 1.3 Hz, 2H), 0.88 (t, *J* = 7.4 Hz, 3H).

^{13}C NMR (151 MHz, DMSO-*d*$_6$) δ: 140.6 (q, *J* = 37.1 Hz), 132.7, 122.1 (q, *J* = 267.9 Hz), 104.4 (q, *J* = 2.2 Hz), 52.1, 32.1, 19.5, 13.8.

^{19}F NMR (376 MHz, DMSO-*d*$_6$) δ: -60.1 (s, 3F).

HRMS (ESI+) calc: $[M+H]^+$ ($C_8H_{12}N_2F_3$) 193.0953; measured: 193.0957 = 2.1 ppm

difference.

IR (neat) $\tilde{\nu}_{max}$/ cm^{-1}: 2924, 2854, 1576, 1232, 1123, 970.

8-(4-(Trifluoromethyl)phenyl)-1,4-dioxa-8-azaspiro[4.5]decane, ***11a***

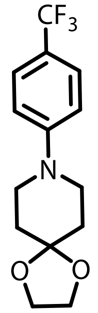

A solution of 4-bromobenzotrifluoride (420 uL, 3.00 mmol, 1 eq.), 1,4-dioxa-8-azaspiro[4.5]decane (769 uL, 6.00 mmol, 2 eq.), Pd_2dba_3 (137 mg, 0.150 mmol, 15 mol%), *rac*-BINAP (187 mg, 0.300 mmol, 10 mol%) and KO^tBu (865 mg, 9.00 mmol, 3 eq.) in toluene (10 mL) was heated to 100 ^oC. After stirring overnight LCMS analysis indicated reaction completion and the reaction solution was filtered over a pad of Celite eluting with $CHCl_3$. The filtrate was concentrated and purified by flash column chromatography on silica gel (eluting with 10% Et_2O in pentane to 44% Et_2O in pentane) to yield **11a** as a colourless solid (530 mg, 62%).

$\mathbf{R_F}$ = 0.4 (30% Et_2O in pentane)

^{1}H NMR (400 MHz, $CDCl_3$) δ: 7.45 (d, *J* = 8.6 Hz, 2H), 6.92 (d, *J* = 8.7 Hz, 2H), 3.98 (s, 4H), 3.58 – 3.14 (m, 4H), 2.29 – 1.57 (m, 4H).

^{13}C NMR (151 MHz, $CDCl_3$) δ: 152.8, 126.5 (q, *J* = 3.9 Hz), 124.9 (q, *J* = 270.4 Hz), 120.0 (q, *J* = 32.6 Hz), 114.8, 107.1, 64.5, 46.6, 34.3.

^{19}F NMR (376 MHz, $CDCl_3$) δ: -61.1 (s, 3F).

Spectral data in accordance with literature.[7]

*tert-Butyl 4-(4-(trifluoromethyl)phenyl)piperazine-1-carboxylate, **12a***

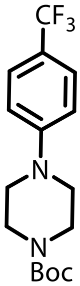

A solution of 4-bromobenzotrifluoride (560 uL, 4.00 mmol, 1 eq.), tert-butyl piperazine-1-carboxylate (1.49 g, 8.00 mmol, 2 eq.), Pd_2dba_3 (183 mg, 0.200 mmol, 5 mol%), *rac*-BINAP (249 mg, 0.400 mmol, 10 mol%) and KO^tBu (1.35 g, 12.0 mmol, 3 eq.) in toluene (10 mL) was heated to 100°C. After stirring overnight GCMS analysis indicated reaction completion and the reaction solution was filtered over a pad of Celite eluting with $CHCl_3$. The filtrate was concentrated and purified by flash column chromatography on silica gel (eluting with 16% Et_2O in pentane to 47% Et_2O in pentane) to yield **12a** as a colourless solid (630 mg, 48%).

R_f = 0.3 (30% Et_2O in pentane)

^{1}H NMR (400 MHz, $CDCl_3$) δ: 7.49 (d, *J* = 8.9 Hz, 2H), 6.92 (d, *J* = 8.6 Hz, 2H), 3.58 (t, *J* = 5.0 Hz, 4H), 3.24 (t, *J* = 5.3 Hz, 4H), 1.49 (s, 9H).

^{13}C NMR (151 MHz, $CDCl_3$) δ: 154.7, 153.2, 126.5 (q, *J* = 3.7 Hz), 124.6 (q, *J* = 270.7 Hz), 121.1 (q, *J* = 32.7 Hz), 115.0, 80.1, 48.1, 48.1, 28.4.

^{19}F NMR (376 MHz, $CDCl_3$) δ: -61.3 (s, 3F).

Spectral data in accordance with literature.[8]

2-Allyl-4-fluoro-1-(trifluoromethyl)benzene, ***17a***

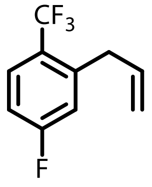

In a Schlenk tube under N_2 was added Mg turnings (292 mg, 12.0 mmol. 1.2 eq.) which were crushed with a spatula. Iodine (*c.a.* 1 crystal), 2-bromo-4-fluoro-benzotrifluoride (1.39 mL, 10.0 mmol 1.0 eq.) and THF (25 mL) were then added to a dropping funnel. A portion was added to just cover the Mg turnings and the suspension was heated to reflux. When the colour had faded, the remaining THF solution was added slowly. The reaction mixture was heated to reflux for 2 hours and then cooled to 0 ^{o}C after which allyl bromide (1.30 mL,15.0 mmol, 1.5 eq.) was then added. The reaction mixture was warmed to RT and stirred overnight. After this, sat. aq. NH_4Cl was added and the mixture extracted with Et_2O. The organic layer was dried over $MgSO_4$, filtered and concentrated. The crude product was purified by flash column chromatography on silica gel (eluting with pentane) to yield **17a** as a colourless oil (1.02 g, 50%).

R_f = 0.3 (5% Et_2O in pentane)

^{1}H NMR (400 MHz, $CDCl_3$) δ: 7.63 (dd, *J* = 8.8, 5.5 Hz, 1H), 7.06 (dd, *J* = 9.6, 2.6 Hz, 1H), 6.98 (td, *J* = 8.3, 2.6 Hz, 1H), 5.93 (ddt, *J* = 16.9, 10.1, 6.7 Hz, 1H), 5.19 – 5.09 (m, 2H), 3.56 (d, *J* = 6.7 Hz, 2H).

^{13}C NMR (151 MHz, $CDCl_3$) δ: 164.5 (dq, *J* = 252.2, 1.5 Hz), 142.2 (dq, *J* = 8.1, 1.5 Hz), 135.4, 128.3 (dq, *J* = 9.3, 5.8 Hz), 124.7 (qd, *J* = 30.5, 3.2 Hz), 124.2 (q, *J* = 273.2 Hz), 118.0 (d, *J* = 22.3 Hz), 117.5, 113.2 (d, *J* = 21.9 Hz), 36.5 (app. p, *J* = 2.0 Hz).

^{19}F NMR (376 MHz, $CDCl_3$) δ: -60.5 (s, 3F), -109.1 – -109.8 (m, 1F).

HRMS (EI+) calc: $[M]^+$ ($C_{10}H_8F_4$) 204.0557; measured: 204.0555 = 0.98 ppm

difference.

IR (neat) $\tilde{\nu}_{max}$/ cm^{-1}: 2989, 2907, 1394, 1066, 1028.

5-Methoxy-1,3-dimethyl-2-(trifluoromethyl)-1H-indole, **26a**

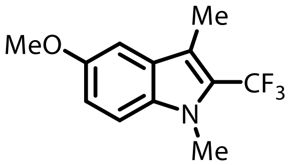

To a solution of 4-methoxyphenylhydrazdine hydrochloride (873 mg, 5.00 mmol. 1.0 eq.) in AcOH (10 mL) was added 1,1,1-Trifluoro-2-butanone (700 μL, 5.25 mmol, 1.05 eq.) and the mixture stirred at 70 °C overnight. The solution was then cooled to RT and H_2O and Et_2O added. The layers were separated and the organic layer dried over $MgSO_4$, filtered and concentrated to yield 5-methoxy-3-methyl-2-(trifluoromethyl)-1H-indole as a yellow solid (987 mg, 86%) which was used without further purification.

In a 20 mL vial, under air, a suspension of NaH (40 mg, 60% dispersion in mineral oil, 1.20 mmol, 1.20 eq.) in DMF (2 mL) was cooled to 0 °C. To this, 5-methoxy-3-methyl-2-(trifluoromethyl)-1H-indole (229 mg, 1.00 mmol, 1.0 eq.) was added portion wise (with care to avoid violent H_2 evolution) and then stirred for 15 minutes at 0 °C then warmed to RT over 45 minutes. MeI (75 μL, 1.10 mmol, 1.1 equiv.) was added dropwise and the reaction was stirred overnight. The following day, the reaction was quenched with sat. aq. NH_4Cl and extracted with Et_2O. The organic layer was washed twice with brine, dried with $MgSO_4$, filtered and concentrated. Purification of the crude mixture using flash column chromatography on silica gel (eluting with 5% Et_2O in pentane to 40% Et_2O in pentane) gave **26a** as a yellow oil which solidified on standing (160 mg, 66%).

R_f = 0.5 (20% Et_2O in pentane)

^{1}H NMR (400 MHz, $CDCl_3$) δ: 7.26 – 7.21 (m, 1H), 7.09 – 6.99 (m, 2H), 3.89 (s, 3H), 3.78 (s, 3H), 2.43 (q, *J* = 2.6 Hz, 3H).

^{13}C NMR (151 MHz, $CDCl_3$) δ: 154.3, 133.1 (q, *J* = 1.7 Hz), 127.1, 123.1 (q, *J* = 35.0 Hz), 122.7 (q, *J* = 269.4 Hz), 115.5, 113.5 (q, *J* = 2.9 Hz), 110.5, 101.0, 55.9, 31.0 (q, *J* = 2.6 Hz), 9.0 (q, *J* = 2.3 Hz).

^{19}F NMR (376 MHz, $CDCl_3$) δ: -57.2 (s, 3F).

HRMS (EI+) calc: $[M]^+$ ($C_{12}H_{12}NOF_3$) 246.0866; measured: 243.08643 = 0.82 ppm

difference.

IR (neat) $\tilde{v}_{max}$/ cm^{-1}: 2951, 2909, 1625, 1492, 1420, 1086.

N-(4-(trifluoromethyl)benzyl)prop-2-en-1-amine, ***18a***

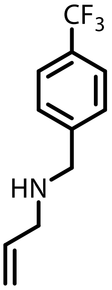

To a solution of 4-(trifluoromethyl)benzaldehyde (1.37 mL, 10.0 mmol, 1 eq.) in 1,2-DCE (35 mL) was added allylamine (788 uL, 10.5 mmol, 1.05 eq.) with stirring. Sodium triacetoxyborohydride (2.97 g, 14.0 mmol, 1.4 eq.) was added in a single portion and the solution stirred overnight at RT. When no aldehyde was observed by TLC, the reaction was quenched with saturated aq. $NaHCO_3$ and Et_2O was added. The layers were separated and the organic layer dried over $MgSO_4$, filtered and concentrated. The crude product was purified by flash column chromatography on silica gel (5% Et_2O in pentane to 70% Et_2O in pentane) to yield **18a** as a colourless oil (1.53 g, 71%).

R_F = 0.1 (30% Et_2O in pentane)

^{1}H NMR (400 MHz, $CDCl_3$) δ: 7.58 (d, *J* = 8.5 Hz, 2H), 7.45 (d, *J* = 8.1 Hz, 2H), 6.01 – 5.82 (m, 1H), 5.30 – 5.06 (m, 2H), 3.85 (s, 2H), 3.27 (ddt, *J* = 5.7, 2.6, 1.5 Hz, 2H), 1.52 (s, 1H).

^{13}C NMR (151 MHz, $CDCl_3$) δ: 144.5 (q, *J* = 1.3 Hz), 136.5, 129.3 (q, *J* = 32.2 Hz), 128.4, 125.3 (q, *J* = 3.7 Hz), 124.4 (q, *J* = 271.6 Hz), 116.3, 52.6, 51.8.

^{19}F NMR (376 MHz, $CDCl_3$) δ: -63.7 (s, 3F).

Spectral data in accordance with literature.[9]

Diethyl 4-(trifluoromethyl)isophthalate, ***25a***

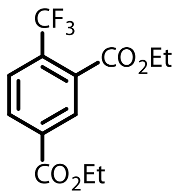

4-Bromoisophthalic acid (1.25 g, 5.1 mmol, 1.0 eq.) was suspended in EtOH (15 mL) and $SOCl_2$ (1.50 mL, 20.4 mmol, 4.0 eq.) added dropwise. The mixture was heated to reflux overnight and the cooled reaction mixture was concentrated. To this was added H_2O and ethyl acetate and the layers separated. The organic layer was washed with sat. aq. $NaHCO_3$ and then dried and concentrated to yield analytically pure diethyl 4-(bromo)isophthalate as a viscous yellow syrup which was used without further purification (980 mg, 60%).

Diethyl 4-(bromo)isophthalate (753 mg, 2.5 mmol, 1.0 eq.) and CuI (500 mg, 2.50 mmol, 1.00 eq.) were added to an oven-dried, vacuum-cooled vial fitted with a rubber septum. DMF (5 mL) and methyl 2,2-difluoro-2-(fluorosulfonyl)acetate (382 μL, 3.00 mmol, 1.2 eq.) was added and, under a positive pressure of N_2, the septum was sealed with a screw cap. The vial was heated to 120 °C overnight and the monitored until complete conversion observed by GCMS. The reaction mixture was filtered through celite eluting with Et_2O and the filtrate washed 5 times with brine, dried with $MgSO_4$, filtered and concentrated under reduced pressure. Flash column chromatography on silica gel (9% Et_2O in pentane) afforded **25a** as a colourless oil (310 mg, 43%).

R_f = 0.3 (5% Et_2O in pentane)

^{1}H NMR (400 MHz, $CDCl_3$) δ: 8.43 – 8.39 (m, 1H), 8.23 (d, *J* = 8.2 Hz, 1H), 7.82 (d, *J* = 8.3 Hz, 1H), 4.51 – 4.34 (m, 4H), 1.50 – 1.31 (m, 6H).

^{13}C NMR (151 MHz, $CDCl_3$) δ: 166.1, 164.5, 132.3, 131.9, 131.1, 127.0 (q, *J* = 5.3 Hz), 122.9 (q, *J* = 273.9 Hz), 62.4, 61.9, 14.2, 13.9.

^{19}F NMR (376 MHz, $CDCl_3$) δ: -60.9 (s, 3F).

HRMS (EI+) calc: $[M]^+$ ($C_{13}H_{13}O_4F_3$) 290.0760; measured: 290.0765 = 1.7 ppm

difference.

IR (neat) $\tilde{\nu}_{max}$/ cm^{-1}: 2986, 1725, 1368, 1308, 1239, 771

Methyl-(R)-2-((tert-butoxycarbonyl)amino)-3-(4-(trifluoromethyl)phenyl)propanoate, ***28a***

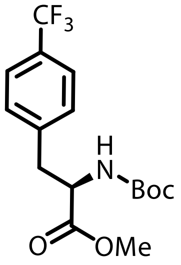

To a solution of L-4-(trifluoromethyl)phenylalanine (1.00 g, 4.30 mmol, 1 eq.) in MeOH (10 mL) at 5 °C was added $SOCl_2$ (1.70 mL, 6.45 mmol, 1.5 eq.) dropwise. The reaction mixture was then heated to 70 °C overnight and concentrated. The crude HCl salt was suspended in $CHCl_3$ (40 mL) and $NaHCO_3$ (2.00 g) added. After stirring for 1 hour, the mixture was filtered and concentrated to yield analytically pure methyl (R)-2-amino-3-(4-(trifluoromethyl)phenyl)propanoate, which was taken forward without further purification.

Methyl (R)-2-amino-3-(4-(trifluoromethyl)phenyl)propanoate (988 mg, 4.00 mmol, 1 eq.) was dissolved in anhydrous DMF (7 mL). To this was added Na_2CO_3 (509 mg, 4.80 mmol, 1.2 eq.) and the suspension was stirred for 5 minutes. Boc_2O (1.10 mL, 4.80 mmol, 1.2 eq.) was added and the reaction stirred at RT. After stirring overnight, full conversion was confirmed by LCMS analysis and the mixture quenched with sat. aq. NH_4Cl and $CHCl_3$ was added. The layers were separated, and the organic layer dried over $MgSO_4$, filtered and concentrated. The crude material was purified was flash column chromatography (eluting with 29% Et_2O in pentane to 32% Et_2O in pentane) to yield **28a** as a colourless solid (978 mg, 70%).

$\mathbf{R_f}$ = 0.3 (10% Et_2O in pentane)

^{1}H NMR (400 MHz, $CDCl_3$) δ: 7.53 (d, *J* = 8.0 Hz, 2H), 7.24 (d, *J* = 8.0 Hz, 2H), 5.06 (d, *J* = 8.2 Hz, 1H), 4.61 (d, *J* = 7.1 Hz, 1H), 3.71 (s, 3H), 3.13 (ddd, *J* = 67.2, 13.8, 6.1 Hz, 2H), 1.39 (s, 9H).

^{13}C NMR (151 MHz, $CDCl_3$) δ: 171.9, 155.0, 140.4, 129.7, 129.3 (d, *J* = 32.2 Hz), 125.4 (q, *J* = 3.7 Hz), 124.2 (q, *J* = 271.9 Hz), 80.1, 54.2, 52.3, 38.2, 28.2.

^{19}F NMR (376 MHz, $CDCl_3$) δ: -63.7 (s, 3F).

Spectral data in accordance with literature.[10]

Ethyl-(R)-2-((tert-butoxycarbonyl)amino)-3-(4-(trifluoromethyl)phenyl)propanoate, ***29a***

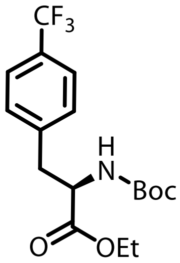

To a solution of L-4-(trifluoromethyl)phenylalanine (1.00 g, 4.30 mmol, 1 eq.) in EtOH (10 mL) at 5 °C was added $SOCl_2$ (1.70 mL, 6.45 mmol, 1.5 eq.) dropwise. The reaction mixture was then heated to 70 °C overnight and concentrated. The crude HCl salt was suspended in $CHCl_3$ (40 mL) and $NaHCO_3$ (2.00 g) added. After stirring for 1 hour, the mixture was filtered and concentrated to yield analytically pure ethyl (R)-2-amino-3-(4-(trifluoromethyl)phenyl)propanoate which was taken forward without further purification.

Ethyl (R)-2-amino-3-(4-(trifluoromethyl)phenyl)propanoate (1.04 g, 4.00 mmol, 1 eq.) was dissolved in anhydrous DMF (7 mL). To this was added Na_2CO_3 (509 mg, 4.80 mmol, 1.2 eq.) and the suspension was stirred for 5 minutes. Boc_2O (1.10 mL, 4.80 mmol, 1.2 eq.) was added and the reaction stirred at RT. After stirring overnight, full conversion was confirmed by LCMS analysis and the mixture quenched with sat. aq. NH_4Cl and $CHCl_3$ was added. The layers were separated, and the organic layer dried over $MgSO_4$, filtered and concentrated. The crude material was purified was flash column chromatography (eluting with 29% Et_2O in pentane to 32% Et_2O in pentane) to yield **29a** as a colourless oil (983 mg, 68%).

R_f = 0.6 (20% Et_2O in pentane)

^{1}H NMR (400 MHz, $CDCl_3$) δ: 7.55 (d, *J* = 8.0 Hz, 2H), 7.26 (d, *J* = 8.1 Hz, 2H), 5.02 (d, *J* = 8.2 Hz, 1H), 4.59 (q, *J* = 6.7 Hz, 1H), 4.17 (q, *J* = 7.1 Hz, 2H), 3.14 (ddd, *J* = 51.1, 13.8, 6.2 Hz, 2H), 1.41 (s, 9H), 1.23 (t, *J* = 7.2 Hz, 3H).

^{13}C NMR (151 MHz, $CDCl_3$) δ: 171.5, 155.0, 140.4 (q, *J* = 1.4 Hz), 129.8, 129.3 (q, *J* = 32.6 Hz), 125.4 (q, *J* = 3.8 Hz), 124.2 (q, *J* = 272.0 Hz), 80.1, 61.6, 54.2, 38.4, 28.3, 14.1.

^{19}F NMR (376 MHz, $CDCl_3$) δ: -63.6 (s, 3F).

HRMS (ESI+) calc: $[M+H]^+$ ($C_{17}H_{23}NO_4F_3$) 362.1574; measured: 362.1566 = 2.2 ppm

difference.

IR (neat) $\tilde{\nu}_{max}$/ cm^{-1}: 3365, 2981, 2935, 1713, 1699, 1323.

(1R,5S)-3-Butyl-1-(4-(trifluoromethyl)phenyl)-3-azabicyclo[3.1.0]hexane, ***31a***

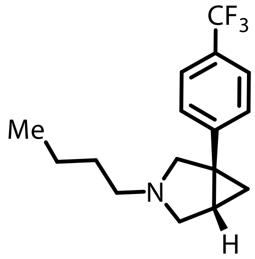

(1R,5S)-1-(4-(trifluoromethyl)phenyl)-3-azabicyclo[3.1.0]hexane (682 mg, 3.00 mmol, 1.0 eq.) was dissolved in acetone (15 mL) and K_2CO_3 (1.24 g, 9.00 mmol, 3.0 eq.) then butyl bromide (484 μL, 4.50 mmol, 1.5 eq.) added. The suspension was heated to reflux overnight and then filtered into H_2O which was then extracted with Et_2O, dried over $MgSO_4$ and concentrated. The crude mixture was purified by flash column chromatography on silica gel (eluting with 6% Et_2O in pentane to 60% Et_2O in pentane) to yield **31a** as a yellow oil (290 mg, 34%).

R_f = 0.5 (20% Et_2O in pentane)

^{1}H NMR (400 MHz, $CDCl_3$) δ: 7.52 (d, *J* = 8.1 Hz, 1H), 7.22 (d, *J* = 8.1 Hz, 1H), 3.36 (d, *J* = 8.4 Hz, 1H), 3.12 (d, *J* = 8.7 Hz, 1H), 2.55 (d, *J* = 8.3 Hz, 1H), 2.50 – 2.42 (m, 3H), 1.75 (ddd, *J* = 8.0, 4.3, 3.5 Hz, 1H), 1.52 (t, *J* = 4.3 Hz, 1H), 1.50 – 1.42 (m, 2H), 1.39 – 1.28 (m, 2H), 0.92 (t, *J* = 7.3 Hz, 3H), 0.83 (dd, *J* = 8.1, 4.3 Hz, 1H).

^{13}C NMR (151 MHz, $CDCl_3$) δ: 147.7 (q, *J* = 1.4 Hz), 127.7 (q, *J* = 32.4 Hz), 126.3, 125.1 (q, *J* = 3.8 Hz), 122.4 (q, *J* = 271.7 Hz),58.4, 55.3, 55.2, 30.9, 30.5, 25.7, 20.6, 18.1, 14.1.

^{19}F NMR (376 MHz, $CDCl_3$) δ: -63.6 (s, 3F).

HRMS (APCI+) calc: $[M+H]^+$ ($C_{16}H_{21}NF_3$) 284.1621; measured: 284.1620 = 0.35 ppm

difference.

IR (neat) $\tilde{\nu}_{max}$/ cm^{-1}: 2958, 2931, 2908, 2791, 1619, 1323.

*(3aR,5R,6S,6aR)-5-((R)-2,2-dimethyl-1,3-dioxolan-4-yl)-2,2-dimethyltetrahydrofuro[2,3-d][1,3]dioxol-6-yl 4-(trifluoromethyl)benzoate, **32a***

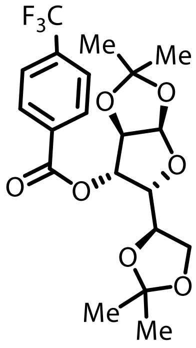

To a solution of 1,2:5,6-Di-*O*-isopropylidene-α-D-glucofuranose (2.00 g, 10.5 mmol, 1.05 eq.), 4-(trifluoromethyl)benzoic acid (2.60 g, 10.0 mmol, 1 eq.) and DCC (2.17 g, 10.5 mmol, 1.05 eq.) in DCM (100 mL) was added DMAP (122 mg, 1.00 mmol, 10 mol%). The reaction mixture was stirred overnight, filtered and concentrated. Purification of the crude mixture with flash column chromatography on silica gel (23% Et_2O in pentane to 75% Et_2O in pentane) in two portions gave **32a** as a colourless foam that solidified in the freezer after several days (3.07 g, 71%).

R_f = 0.3 (50% Et_2O in pentane)

^{1}H NMR (400 MHz, $CDCl_3$) δ: 8.14 (d, *J* = 8.1 Hz, 2H), 7.73 (d, *J* = 8.0 Hz, 2H), 5.96 (d, *J* = 3.6 Hz, 1H), 5.52 (d, *J* = 2.5 Hz, 1H), 4.64 (d, *J* = 3.6 Hz, 1H), 4.39 – 4.29 (m, 2H), 4.18 – 4.02 (m, 2H), 1.56 (s, 3H), 1.41 (s, 3H), 1.33 (s, 3H), 1.26 (s, 3H).

^{13}C NMR (151 MHz, $CDCl_3$) δ: 163.9, 134.8 (q, *J* = 32.7 Hz), 132.7 (q, *J* = 1.3 Hz), 130.0, 125.5 (q, *J* = 3.7 Hz), 123.4 (q, *J* = 272.8 Hz), 112.4, 109.4, 105.0, 83.2, 79.8, 77.1, 72.4, 67.3, 26.7, 26.6, 26.1, 25.1.

^{19}F NMR (376 MHz, $CDCl_3$) δ: -64.3 (s, 3F).

Spectral data in accordance with literature.[11]

4-(Trifluoromethyl)-1,1'-biphenyl, ***34a***

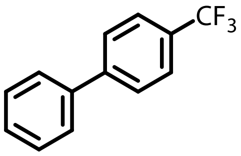

To a 20 mL screw-capped vial was added 4-bromobenzotrifluoride (657 mg, 3.00 mmol, 1 eq.), phenylboronic acid (403 mg, 3.30 mmol, 1.10 eq.) and $Pd(PPh_3)_4$ (87 mg, 0.075 mmol, 0.030 eq.).1,2-Dimethoxyethane (7 mL) and 2 M aq. Na_2CO_3 (3 mL) were then added and he vial was capped and stirred at 100 °C overnight. The next day, the reaction mixture was filtered through celite eluting with chloroform and the solvent removed under reduced pressure. Flash column chromatography on silica gel (eluting with 5% ethyl acetate in pentane) afforded **34a** as a colourless powder (585 mg, 88 %).

R_F = 0.6 (Pentane)

^{1}H NMR (500 MHz, $CDCl_3$) δ: 7.64 (d, *J* = 7.6 Hz, 2H), 7.54 – 7.51 (m, 2H), 7.42 (tt, *J* = 7.6, 1.3 Hz, 1H).

^{13}C NMR (126 MHz, $CDCl_3$) δ: 144.9 (q, *J* = 1.4 Hz), 139.9, 129.5 (q, *J* = 32.5 Hz), 129.1, 128.3, 127.6, 127.4, 125.9 (q, *J* = 3.8 Hz), 124.5 (q, *J* = 271.8 Hz).

^{19}F NMR (377 MHz, $CDCl_3$) δ: -62.3 (s, 3F).

Spectral data in accordance with literature.[12]

General Hydrodefluorination Procedures

General Procedure A - Standard 0.5 mmol scale procedure

An oven-dried (180°C) H-type divided cell equipped with a porous glass frit was evacuated and backfilled with N_2 three times. $TEAPF_6$ (275 mg, 1 mmol, 2 equiv.) was added to both the cathode and anode compartments. Trifluoromethylarene substrate (0.5 mmol, 1 equiv.) was added to the cathode compartment and Bu_4NBr (480 mg, 1.5 mmol, 3 equiv.) was added to the anode compartment. To each compartment was added (with stirring, 900 RPM) degassed, anhydrous MeCN (2.5 mL, 0.2 M) and then TMSCl (0.19 mL, 1.5 mmol, 3 equiv.). Under a strong flow of N_2 the septa were replaced with ones containing a Pt wire electrode (entire coil area made of ~ 10 cm Pt wire submerged) as the anode and a Ni foil electrode (submerged surface area 2.5 cm^2). A constant current of -5.0 mA was applied for 19320 s (2 *F*). When the electrolysis was finished, the catholyte was concentrated directly onto silica and purified by flash column chromatography or concentrated, dissolved in MeOH and purified by preparative HPLC.

General Procedure B - Standard larger scale (5.0 mmol) procedure

An oven-dried (180°C) H-type divided cell equipped with a porous glass frit was evacuated and backfilled with N_2 three times. $TEAPF_6$ (2.75 g, 10.0 mmol, 2 equiv.) was added to both the cathode and anode compartments. 1-(benzyloxy)-3-(trifluoromethyl)benzene (1.26 g, 5.00 mmol, 1 equiv.) was added to the cathode compartment and Bu_4NBr (7.20 g, 15.0 mmol, 3 equiv.) was added to the anode compartment. To each compartment was added (with stirring, 900 RPM) degassed, anhydrous MeCN (25 mL, 0.2 M) and then TMSCl (1.90 mL, 15.0 mmol, 3 equiv.). Under a strong flow of N2 the septa were replaced with ones containing a Graphite rod electrode (60 mm diameter, 6.0 cm length submerged) as the anode and a Ni foil electrode (submerged surface area 25 cm^2). A constant current of -20.0 mA was applied for 48300 s (2 *F*). When the electrolysis was finished, the catholyte was concentrated directly onto silica and purified by flash column chromatography.

General procedure C - Standard 0.25 mmol scale double hydrodefluorination procedure

An oven-dried (180°C) H-type divided cell equipped with a porous glass frit was evacuated and backfilled with N_2 three times. $TEAPF_6$ (550 mg, 2.0 mmol, 4 equiv.) was added to both the cathode and anode compartments. Trifluoromethylarene substrate (0.25 mmol, 1 equiv.) was added to the cathode compartment and Bu_4NBr (480 mg, 1.5 mmol, 6 equiv.) was added to the anode compartment. To each compartment was added (with stirring, 900 RPM) degassed, anhydrous MeCN (5.0 mL, 0.1 M) and then TMSCl (0.19 mL, 1.5 mmol, 3 equiv.). Under a strong flow of N2 the septa were replaced with ones containing a Pt wire electrode (entire coil area made of ~ 10 cm Pt wire submerged) as the anode and a Ni foil electrode (submerged surface area 5.0 cm^2). A constant current of -5.0 mA was applied for 21000 s (4.2 *F*). After the electrolysis had ended, the catholyte was collected *via* pipette and quenched using TBAF 1 M in THF (2.0 mL, 2.0 mmol, 8 eq.). After concentration, trituration of the solids in ether, filtration, the crude product was either loaded onto silica and purified by silica gel chromatography or concentrated, dissolved in MeOH and purified by preparative HPLC.

Note: Decreasing the substrate concentration through dilution (5 mL MeCN vs 2.5 mL) resulted in an increase in electrode surface area as more was submerged. This lowered the current density and resulted in better selectivity and less overreduction.

Electrochemical set-up

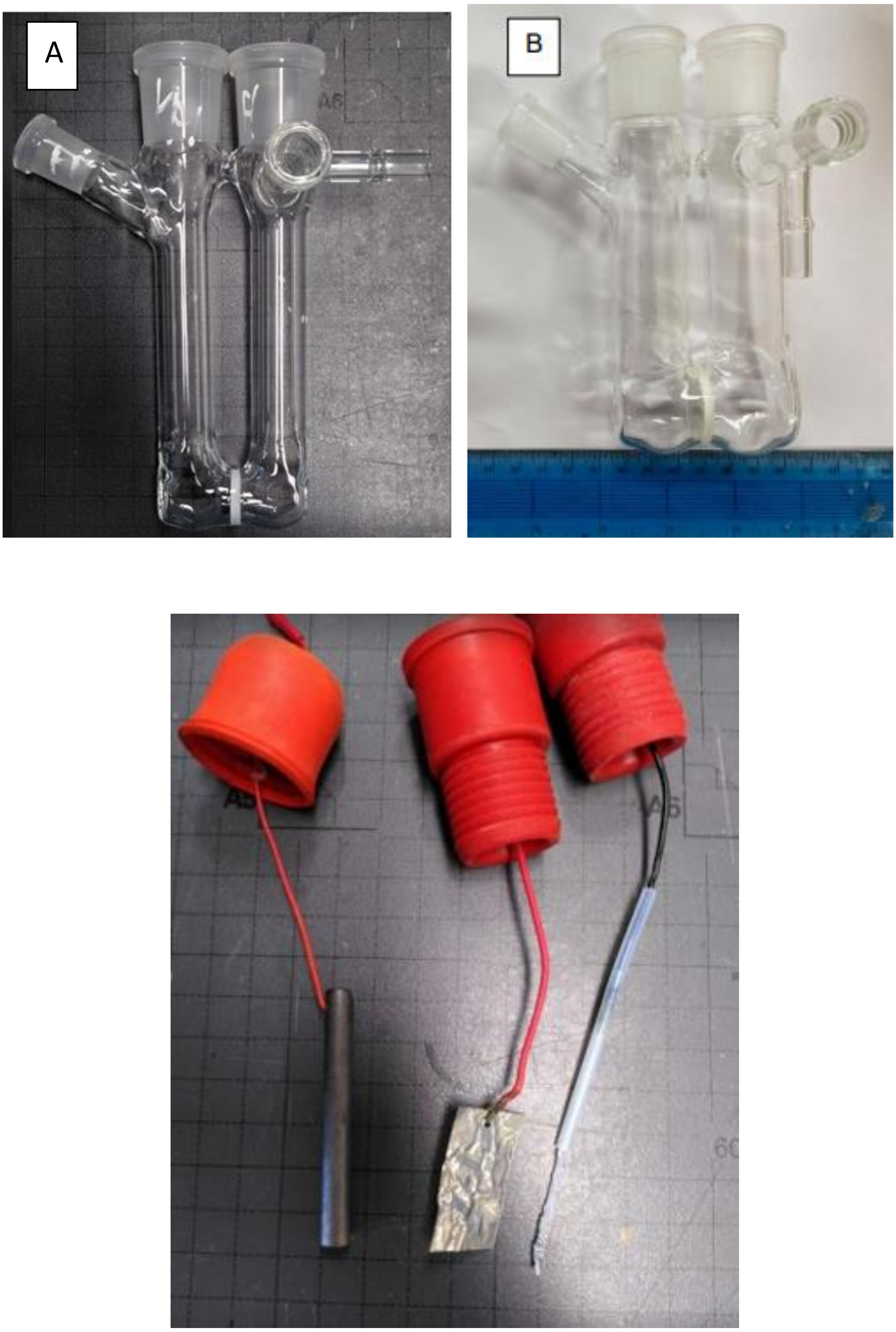

Figure S17. Set-up photos.

All cyclic voltametric (CV) and chronopotentiometry experiments were performed at room temperature using an Autolab M101, MultiPalmsens 4 or ElectraSyn 2.0 (purchased from IKA). CV experiments were carried out with a working electrode (GC = glassy carbon, Pt, Au, Ni = 1-3 mm diameter), a counter electrode (platinum wire) and a 0.1 M Ag/$AgNO_3$ reference electrode. All working electrodes were polished before each experiment. Before each CV, the solution was stirred for approximately 10 seconds, whilst being degassed by a stream of N_2. The CV cell was maintained under an atmosphere of N_2 during analysis.

- Pt wire was purchased from Advent research materials: diameter 0.4 mm, 99.99% temper annealed, part number PT5441

https://www.advent-rm.com/en-GB/Products/Pure-Metals/Platinum/Form/Wire/Line/PT5441

Platinum electrodes were made by wrapping platinum wire around PTFE tubing to create a surface area approximately ~1 cm^2. The platinum wire was then fed through PTFE tubing by creating a small hole on the side of the tubing. The wire was then spot- welded to a copper wire. Using a large gauge needle as a guide, the wire was fed through a new suba seal.

- Ni foil was purchased from Alfa Aesar: 0.05mm (0.002in) thick, annealed, 99+% (metals basis), part number 42634.CH and cut to size with scissors.

https://www.alfa.com/en/catalog/042634/

- Graphite rods were purchased from Alfa Aesar (6.3mm (0.25in) dia. x 61cm (24in) long, 99% (metals basis)), part number 10135, and machined to size in house.

https://www.alfa.com/en/catalog/010135/

Electrochemical cell set-up is as described in our previous report[32] utilising an in-house designed glass H-cell, with a porous glass frit (pore 3) divider separating two compartments with a cell volume of either 3 or 25 mL on each side.

Notes on setting up electrochemical hydrodefluorination reactions:

- Anhydrous and degassed solvents are essential, as well as anaerobic conditions

Use of wet MeCN leads to hydrolysis of TMSCl – the resulting HCl is easier to reduce than the substrate and so cathodic hydrogen evolution is the major process, reducing the faradaic efficiency of the process. Furthermore, water/oxygen is more easily reduced than the Ar-CF_3 substrates and the resulting reduced species may result in undesired decomposition side reactions.

- Working up the reaction

It was found that diffusion of Bu_4NBr_3 (the oxidation product formed at the anode) as well as substrate diffusion was minimal during the course of the electrolysis. However, when leaving the completed reaction in the H-Cell without current for extended periods of time, it was found that substantial diffusion occurred. This is most problematic for electron rich products/starting materials, which were found to undergo unwanted bromination reactions with Bu_4NBr_3 upon mixing. A blue colour may also be observed in catholyte if diffusion occurs with the Ni foil electrode still submerged. This arises from the oxidation of Ni by Bu_4NBr_3. Hence, it was found to be important to work up the reaction as soon as possible after the reaction is finished.

- Repeated use of electrodes

Diminished yields were observed with Ni foil that had been used more than once – to avoid this, a new piece of Ni foil was used for each electrolysis. The Pt wire coil electrodes could be rinsed with MeCN and allowed to dry in air for repeating use without affecting reaction yield. After multiple uses, it was found that the wire connections to the Ni foil corroded – this results in a lower observed cell voltage (*c.a* -1.8 V instead of -3.5 V) and no substrate conversion is observed. In these cases, replacing the electrical wire used rectifies the issue. If this persists, connection *via* Ni wire is a suitable alternative.

- H-cell cleaning

After the reaction he glass cells are washed with MeCN. The frit can be washed if one compartment is filled with MeCN and the cell is laid on its side: gravity will push the solvent through the frit, rinsing through any trapped material. This is repeated until the frit and the liquid passing through it are colourless. Careful drying with nitrogen is required before putting the cell in the oven to avoid pyrolysis of residual solvent. A change in the rate of liquid passing through the frit by action of gravity during cleaning can be an indication of frit damage – this may change the rate of diffusion which can lead to decreased reaction efficiency.

*4-Fluoro-(difluoromethyl)benzene, **1b***

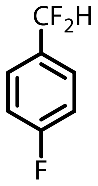

1b was synthesised from 4-fluoro-(trifluoromethyl)benzene following General Procedure A, owing to its volatility a spectroscopic yield is given (76%). Diagnostic NMR signals given below, confirmed through spiking authentic commercial material into crude reaction mixture.

^{1}H NMR (400 MHz, $CDCl_3$) δ: 6.57 (t, *J* = 56.3 Hz, 1H).

^{19}F NMR (376 MHz, $CDCl_3$) δ: -109.6 (d, *J* = 56.4 Hz, 2F).

Data in accordance with the literature.[13]

*(Difluoromethyl)benzene, **2b***

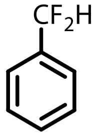

2b was synthesised from (trifluoromethyl)benzene following General Procedure A, owing to its volatility a spectroscopic yield is given (80%). Diagnostic NMR signals given below, confirmed through spiking authentic commercial material into crude reaction mixture.

^{1}H NMR (400 MHz, $CDCl_3$) δ: 6.65 (t, *J* = 56.7 Hz, 1H)

^{19}F NMR (376 MHz, $CDCl_3$) δ: -110.6 (d, *J* = 56.5 Hz, 2F).

Data in accordance with the literature.[14]

N-(4-(difluoromethyl)phenyl)acetamide, ***3b***

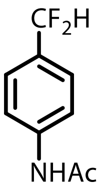

3b was synthesised from N-(4-(trifluoromethyl)phenyl)acetamide following General Procedure A and purified by using silica gel chromatography (30% Et_2O in pentane to 50% Et_2O in pentane) to yield a colourless solid (49 mg, 51%) that co-eluted alongside 5% remaining starting material.

$\mathbf{R_f}$ = 0.2 (20% Et_2O in pentane)

^{1}H NMR (400 MHz, $CDCl_3$) δ: 7.60 (d, *J* = 8.4 Hz, 2H), 7.46 (d, *J* = 8.3 Hz, 2H), 7.35 (br. s, 1H), 6.61 (t, *J* = 56.6 Hz, 1H), 2.20 (s, 3H).

^{13}C NMR (151 MHz, $CDCl_3$) δ: 169.1, 138.4, 135.0 (t, J = 22.3 Hz), 129.1, 122.1, 121.2 (t, *J* = 6.0 Hz), 117.1 (t, *J* = 6.4 Hz), 114.3 (t, J = 239.0 Hz), 24.3.

^{19}F NMR (376 MHz, $CDCl_3$) δ: -109.6 (d, *J* = 56.5 Hz, 2F).

Data in accordance with the literature.[1]

*4'-(Difluoromethyl)-2,6-dimethyl-1,1'-biphenyl, **4b***

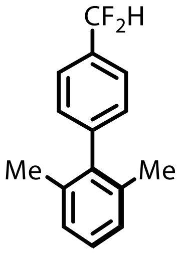

4b was synthesised from **4a** following General Procedure A and purified by using silica gel chromatography (pentane) to yield a colourless solid (67 mg, 58%).

R$_f$ = 0.5 (20% Et_2O in pentane)

^{1}H NMR (400 MHz, $CDCl_3$) δ: 7.64 (dt, *J* = 8.4, 1.2 Hz, 2H), 7.35 – 7.29 (m, 2H), 7.25 (dd, *J* = 8.3, 6.6 Hz, 1H), 7.18 (d, *J* = 7.5 Hz, 2H), 6.77 (t, *J* = 56.6 Hz, 1H), 2.08 (s, 6H).

^{13}C NMR (151 MHz, $CDCl_3$) δ: 143.8 (t, *J* = 2.1 Hz), 140.8, 135.9, 132.8 (t, *J* = 22.4 Hz), 129.5, 127.44, 127.43, 125.8 (t, *J* = 6.0 Hz), 114.9 (t, *J* = 238.5 Hz), 20.8.

^{19}F NMR (376 MHz, $CDCl_3$) δ: -110.1 (d, *J* = 56.5 Hz, 2F).

HRMS (EI+) calc: $[M]^+$ ($C_{15}H_{14}F_2$) 232.1058; measured: 232.1059 = 0.43 ppm

difference.

IR (neat) $\tilde{\nu}_{max}$/ cm^{-1}: 3021, 2959, 1376, 1070, 1027.

*Ethyl 4-(difluoromethyl)benzoate, **5b***

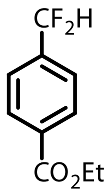

5b was synthesised from ethyl 4-(trifluoromethyl)benzoate following General Procedure A however prior to concentration TBAF (1 M in THF) (0.5 mL, 0.5 mmol) was added to the crude mixture in a vial and agitated for 5 minutes. After this the residue was purified by using silica gel chromatography (15% Et_2O in pentane to 30% Et_2O in pentane) to yield a colourless oil (64 mg, 58%) contaminated with *c.a* 8% of the analogous CFH_2 product **5c** which co-elutes.

$\mathbf{R_f}$ = 0.5 (20% Et_2O in pentane)

^{1}H NMR (400 MHz, $CDCl_3$) δ: 8.13 (d, *J* = 7.8 Hz, 2H), 7.58 (d, *J* = 8.7 Hz, 2H), 6.69 (t, *J* = 56.1 Hz, 1H), 4.40 (q, *J* = 7.2 Hz, 2H), 1.41 (t, *J* = 7.1 Hz, 3H).

^{13}C NMR (151 MHz, $CDCl_3$) δ: 165.8, 138.4 (t, *J* = 22.4 Hz), 132.7 (t, *J* = 1.9 Hz), 129.9, 125.6 (t, *J* = 6.0 Hz), 114.0 (t, *J* = 239.7 Hz), 61.4, 14.3.

^{19}F NMR (376 MHz, $CDCl_3$) δ: -113.4 (d, *J* = 55.6 Hz).

Data in accordance with the literature.[15]

*1-(Difluoromethyl)-4-methoxybenzene, **6b***

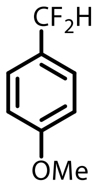

6b was synthesised from 1-(trifluoromethyl)-4-methoxybenzene following General Procedure A, owing to its volatility a spectroscopic yield is given (64%). Diagnostic NMR signals given below, confirmed through literature comparison.

^{1}H NMR (400 MHz, $CDCl_3$) δ: 7.35 (d, J = 8.9 Hz, 1H), 6.87 (d, J = 8.0 Hz, 2H), 6.53 (t, J = 56.7 Hz, 2H), 3.76 (s, 3H).

^{19}F NMR (376 MHz, $CDCl_3$) δ: -108.2 (d, J = 56.5 Hz, 2F).

Data in accordance with the literature.[13]

*10-(4-(Difluoromethyl)phenyl)-10H-phenothiazine,**7b***

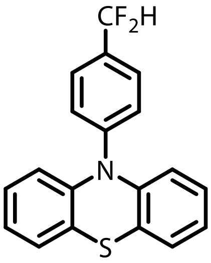

7b, was synthesised from **7a** following General Procedure x and purified by using silica gel chromatography (5% Et_2O in pentane to 15% Et_2O in pentane) to yield a colourless solid (59 mg, 36%).

R_f = 0.5 (20% Et_2O in pentane)

^{1}H NMR (400 MHz, $CDCl_3$) δ: 7.70 (d, *J* = 8.4 Hz, 2H), 7.44 (d, *J* = 8.7 Hz, 2H), 7.15 (dd, *J* = 7.5, 1.7 Hz, 2H), 7.06 – 6.89 (m, 4H), 6.74 (t, *J* = 56.4 Hz, 1H), 6.44 (dd, *J* = 8.0, 1.3 Hz, 2H).

^{13}C NMR (151 MHz, $CDCl_3$) δ: 144.3, 143.4, 132.7 (t, *J* = 22.7 Hz), 128.2, 127.9 (t, *J* = 5.9 Hz), 127.3, 127.0, 123.5, 123.3, 118.3, 114.4 (t, *J* = 238.9 Hz).

^{19}F NMR (376 MHz, $CDCl_3$) δ: -110.2 (d, *J* = 56.4 Hz, 2F).

HRMS (ESI+) calc: $[M]^+$ ($C_{19}H_{13}NSF_2$) 325.0731; measured: 325.0729 = 0.62 ppm difference.

IR (neat) $\tilde{\nu}_{max}$/ cm^{-1}: 2958, 2924, 1460, 1307, 745.

2-(Difluoromethyl)-1,3,5-trimethoxybenzene, ***8b***

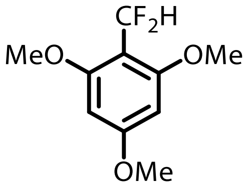

8b was synthesised from **8a** following General Procedure A. **8b** decomposed on silica and in $CDCl_3$ to aldehyde **8e**. Characteristic signals from crude reaction mixture given below for **8b**:

^{1}H NMR (400 MHz, $CDCl_3$) δ: 7.04 (t, *J* = 54.4 Hz, 1H), 6.10 (s, 2H), 3.83 (s, 9H).

^{19}F NMR (376 MHz, $CDCl_3$) δ: -116.0 (d, *J* = 54.4 Hz, 2F).

2,4,6-Trimethoxybenzaldehyde, ***8e***

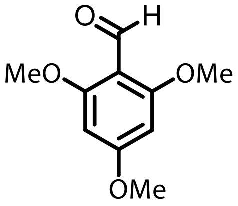

8e was isolated from column chromatography (pentane) of **8b** to yield a colourless solid (63 mg, 58% *wrt* **8a**).

R_f = 0.2 (pentane)

^{1}H NMR (400 MHz, $CDCl_3$) δ: 10.31 (s, 1H), 6.03 (s, 2H), 3.83 (s, 9H).

^{13}C NMR (151 MHz, $CDCl_3$) δ: 187.7, 166.3, 164.1, 108.8, 90.3, 56.0.

Data in accordance with the literature.[16]

*1-Butyl-3-(difluoromethyl)-1H-pyrazole, **9b***

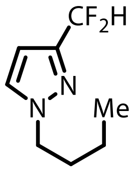

9b was synthesised from **9a** following General Procedure A and purified by using silica gel chromatography (10% Et_2O in pentane to 20% Et_2O in pentane) to yield a colourless oil (19 mg, 22%).

R_f = 0.5 (20% Et_2O in pentane)

^{1}H NMR (400 MHz, $CDCl_3$) δ: 7.39 (d, *J* = 2.4 Hz, 1H), 6.68 (t, *J* = 55.3 Hz, 1H), 6.45 (dt, *J* = 2.3, 1.1 Hz, 1H), 4.12 (t, *J* = 7.2 Hz, 2H), 1.88 – 1.80 (m, 2H), 1.40 – 1.28 (m, 2H), 0.94 (t, *J* = 7.4 Hz, 3H).

^{13}C NMR (151 MHz, $CDCl_3$) δ: 146.4 (t, *J* = 29.3 Hz), 130.3, 111.3 (t, *J* = 233.3 Hz), 103.0 (t, *J* = 1.7 Hz), 52.3, 32.3, 19.8, 13.5.

^{19}F NMR (376 MHz, $CDCl_3$) δ: -112.2 (d, *J* = 55.4 Hz, 2F).

HRMS (ESI+) calc: $[M+H]^+$ ($C_8H_{13}N_2F_2$) 175.1047; measured: 175.1052 = 2.9 ppm

difference.

IR (neat) $\tilde{\nu}_{max}$/ cm^{-1}: 2917, 2849, 1739, 1066, 1027.

4-(Difluoromethyl)-N,N-dimethylaniline, ***10b***

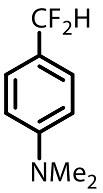

10b was synthesised from 4-(trifluoromethyl)-N,N-dimethylaniline following General Procedure A. **10b** decomposed on silica to aldehyde **10e**. Characteristic signals from crude reaction mixture given below for **10b**:

^{1}H NMR (400 MHz, $CDCl_3$) δ: 7.46 – 7.33 (m, 4H), 6.53 (t, *J* = 56.5 Hz, 1H), 3.01 (s, 6H).

^{19}F NMR (376 MHz, $CDCl_3$) δ: -115.1 (d, *J* = 56.4 Hz, 2F).

4-(Dimethylamino)benzaldehyde, ***10e***

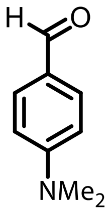

10e was isolated from **10b** following column chromatography (30% Et_2O in pentane to 50% Et_2O in pentane)) to yield a light yellow solid (56 mg, 75% *wrt* **10a**).

R_f = 0.3 (20% Et_2O in pentane)

^{1}H NMR (400 MHz, $CDCl_3$) δ: 9.73 (s, 1H), 7.73 (d, *J* = 8.9 Hz, 2H), 7.24 – 6.56 (m, 2H), 3.08 (s, 6H).

^{13}C NMR (151 MHz, $CDCl_3$) δ: 190.3, 154.3, 131.8, 125.0, 111.0, 40.0.

Data in accordance with the literature.[17]

8-(4-(Difluoromethyl)phenyl)-1,4-dioxa-8-azaspiro[4.5]decane, ***11b***

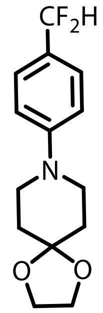

11b was synthesised from **11a** following General Procedure A and purified by using silica gel chromatography (25% Et_2O in pentane to 45% Et_2O in pentane) to yield a colourless solid (77 mg, 57%).

R_f = 0.3 (20% Et_2O in pentane)

^{1}H NMR (400 MHz, $CDCl_3$) δ: 7.37 (d, *J* = 8.9 Hz, 2H), 6.95 (d, *J* = 8.8 Hz, 2H), 6.56 (t, *J* = 56.9 Hz, 1H), 3.99 (s, 4H), 3.44 – 3.38 (m, 4H), 1.86 – 1.77 (m, 4H).

^{13}C NMR (151 MHz, $CDCl_3$) δ: 152.3 (t, *J* = 1.7 Hz), 126.7 (t, *J* = 5.9 Hz), 124.4 (t, *J* = 22.7 Hz), 115.4, 115.3 (t, *J* = 236.6 Hz), 107.1, 64.4, 46.9, 34.3.

^{19}F NMR (376 MHz, $CDCl_3$) δ: -108.8 (d, *J* = 56.9 Hz, 2F).

HRMS (ESI+) calc: $[M+H]^+$ ($C_{14}H_{18}F_2NO_2$) 270.1300; measured: 270.1290 = 3.7 ppm

difference.

IR (neat) $\tilde{v}_{max}$/ cm^{-1}: 2989, 2965, 1618, 1369, 1003, 838.

Tert-butyl 4-(4-(difluoromethyl)phenyl)piperazine-1-carboxylate, ***12b***

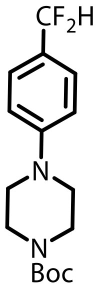

12b was synthesised from **12a** following General Procedure A and purified by using silica gel chromatography (15% Et_2O in pentane to 30% Et_2O in pentane) to yield a colourless solid (73 mg, 47%).

R_f = 0.4 (20% Et_2O in pentane)

^{1}H NMR (400 MHz, $CDCl_3$) δ: 7.40 (d, *J* = 8.3 Hz, 2H), 6.94 (d, *J* = 8.3 Hz, 2H), 6.57 (t, *J* = 56.8 Hz, 1H), 3.58 (t, *J* = 5.2 Hz, 4H), 3.20 (t, *J* = 5.2 Hz, 4H), 1.48 (s, 9H).

^{13}C NMR (151 MHz, $CDCl_3$) δ: 154.8, 152.9 (t, *J* = 1.7 Hz), 126.9 (t, *J* = 5.9 Hz), 125.5 (t, *J* = 22.8 Hz), 115.7, 115.2 (t, *J* = 236.9 Hz), 80.2, 48.6, 48.6, 28.6.

^{19}F NMR (376 MHz, $CDCl_3$) δ: -108.0 (d, *J* = 56.8 Hz, 2F).

HRMS (ESI+) calc: $[M+H]^+$ ($C_{16}H_{23}N_2O_2F_2$) 313.1722; measured: 313.1717 = 1.6 ppm

difference.

IR (neat) $\tilde{v}_{max}$/ cm^{-1}: 2949, 2844, 1323, 1104, 976.

*1-(Benzyloxy)-3-(difluoromethyl)benzene, **13b***

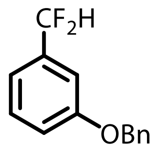

13b was synthesised from 1-(benzyloxy)-3-(trifluoromethyl)benzene following General Procedure A and purified by using silica gel chromatography (34% Et_2O in pentane to 55% Et_2O in pentane)) to yield a colourless solid (74 mg, 63%).

13b was synthesised from 1-(benzyloxy)-3-(trifluoromethyl)benzene following General Procedure B and purified by using silica gel chromatography (34% Et_2O in pentane to 55% Et_2O in pentane)) to yield an off-white solid (618 mg, 53%).

R_f = 0.4 (20% Et_2O in pentane)

^{1}H NMR (400 MHz, $CDCl_3$) δ: 7.53 – 7.32 (m, 6H), 7.19 – 7.05 (m, 3H), 6.62 (t, *J* = 56.5 Hz, 1H), 5.10 (s, 2H).

^{13}C NMR (151 MHz, $CDCl_3$) δ: 159.0, 136.5, 135.8 (t, *J* = 22.3 Hz), 130.0, 128.7, 128.2, 127.6, 118.1 (t, *J* = 6.2 Hz), 117.4 (t, *J* = 1.9 Hz), 114.5 (t, *J* = 239.1 Hz), 111.8 (t, *J* = 6.2 Hz), 70.2.

^{19}F NMR (376 MHz, $CDCl_3$) δ: -110.6 (d, *J* = 56.6 Hz, 2F).

Data in accordance with the literature.[18]

*1-(Difluoromethyl)-3-methoxybenzene, **14b***

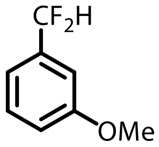

14b was synthesised from 1-(trifluoromethyl)-3-methoxybenzene following General Procedure A, owing to its volatility a spectroscopic yield is given (65%). Diagnostic NMR signals given below, confirmed through literature comparison.

^{1}H NMR (400 MHz, $CDCl_3$) δ: 6.54 (t, *J* = 56.5 Hz, 1H), 3.76 (s, 3H).

^{19}F NMR (376 MHz, $CDCl_3$) δ: -110.6 (d, *J* = 56.4 Hz, 2F).

Data in accordance with the literature.[19]

Diethyl (4-(difluoromethyl)benzyl)phosphonate, ***15b***

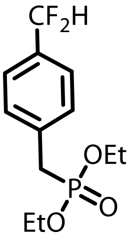

15b was synthesised from *diethyl (4-(trifluoromethyl)benzyl)phosphonate* following General Procedure A and purified by trituration of the concentrated catholyte with cold Et_2O to yield a colourless oil (94 mg, 60%) contaminated with *c.a.* 8% remaining starting material. **15b** was found to be slightly unstable on silica and so was not subjected to further purification, the yield has been adjusted to reflect this.

R_f = 0.5 (20% Et_2O in pentane)

^{1}H NMR (400 MHz, $CDCl_3$) δ: 7.42 (d, *J* = 7.6 Hz, 2H), 7.39 (d, *J* = 4.1 Hz, 2H), 6.62 (t, *J* = 56.4 Hz, 1H), 4.05 – 3.99 (m, 4H), 3.18 (d, *J* = 21.7 Hz, 2H), 1.24 (t, *J* = 7.1 Hz, 6H).

^{13}C NMR (151 MHz, $CDCl_3$) δ: 134.7 (td, *J* = 22.3, 3.1 Hz), 132.6 (d, *J* = 9.2 Hz), 132.1 (dt, *J* = 6.4, 1.9 Hz), 128.9 (d, *J* = 3.1 Hz), 114.6 (t, *J* = 238.9 Hz), 62.2 (d, *J* = 6.7 Hz), 33.7 (d, *J* = 138.5 Hz), 16.3 (d, *J* = 6.0 Hz).

^{19}F NMR (376 MHz, $CDCl_3$) δ: -111.9 (d, *J* = 56.5 Hz, 2F).

HRMS (ESI+) calc: $[M+H]^+$ ($C_{12}H_{18}O_3F_2P$) 279.0956; measured: 279.0951 = 1.8 ppm

difference.

IR (neat) $\tilde{v}_{max}$/ cm^{-1}: 3462, 2984, 2912, 1051, 1019.

*1-Allyl-4-(difluoromethyl)benzene, **16b***

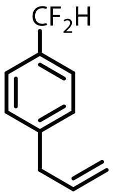

16b was synthesised from 4-trifluoromethyl-1-allylbenzene following General Procedure A and purified by using silica gel chromatography (pentane) to yield a colourless oil (57 mg, 68%).

R_f = 0.5 (20% Et_2O in pentane)

^{1}H NMR (400 MHz, $CDCl_3$) δ: 7.45 (d, *J* = 7.7 Hz, 2H), 7.29 (d, *J* = 7.8 Hz, 2H), 6.63 (t, *J* = 56.6 Hz, 1H), 5.97 (ddt, *J* = 17.0, 10.7, 6.7 Hz, 1H), 5.14 – 5.12 (m, 1H), 5.11 – 5.07 (m, 1H), 3.44 (d, *J* = 6.9 Hz, 2H).

^{13}C NMR (151 MHz, $CDCl_3$) δ: 143.0 (t, *J* = 2.0 Hz), 136.7, 132.3 (t, *J* = 22.4 Hz), 128.9, 125.7 (t, *J* = 6.0 Hz), 116.4, 114.9 (t, *J* = 238.2 Hz), 40.0.

^{19}F NMR (376 MHz, $CDCl_3$) δ: -109.8 (d, *J* = 56.3 Hz, 2F).

HRMS (EI+) calc: $[M]^+$ ($C_{10}H_{10}F_2$) 167.0745; measured: 168.0743 = 1.2 ppm difference.

IR (neat) $\tilde{\nu}_{max}$/ cm^{-1}: 2960, 2925, 1252, 1080, 992, 847.

*2-Allyl-1-(difluoromethyl)-4-fluorobenzene, **17b***

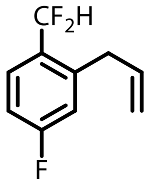

17b was synthesised from **17a** following General Procedure A and purified by using silica gel chromatography (pentane) to yield a colourless oil (65 mg, 70%).

R_f = 0.3 (5% Et_2O in pentane)

^{1}H NMR (400 MHz, $CDCl_3$) δ: 7.54 (dd, *J* = 8.4, 5.7 Hz, 1H), 7.04 – 6.94 (m, 2H), 6.76 (t, *J* = 55.3 Hz, 1H), 5.94 (ddt, *J* = 16.8, 10.2, 6.4 Hz, 1H), 5.25 – 5.00 (m, 2H), 3.51 (d, *J* = 6.4 Hz, 2H).

^{13}C NMR (151 MHz, $CDCl_3$) δ: 164.0 (dt, *J* = 250.1, 2.1 Hz), 141.1 (dt, *J* = 8.0, 4.5 Hz), 135.4, 128.2 (dt, *J* = 9.1, 7.4 Hz), 117.3, 117.2 (d, *J* = 22.2 Hz), 113.5 (t, *J* = 263.4 Hz), 113.4 (d, *J* = 3.4 Hz), 36.1 (d, *J* = 1.4 Hz).

^{19}F NMR (376 MHz, $CDCl_3$) δ: -111.0 – -111.3 (m, 1F), -112.2 (d, *J* = 55.5 Hz, 2F).

HRMS (EI+) calc: $[M]^+$ ($C_{10}H_9F_3$) 186.0651; measured: 186.0651 = 0.54 ppm

difference.

IR (neat) $\tilde{\nu}_{max}$/ cm^{-1}: 2923, 2054, 1394, 1379, 1078, 1066.

N-(4-(difluoromethyl)benzyl)prop-2-en-1-amine, ***18b***

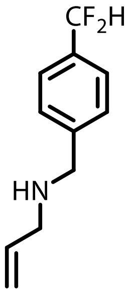

18b was synthesised from **18a** following General Procedure A without TMSCl and purified by using silica gel chromatography (20% Et_2O in pentane to 30% Et_2O in pentane) to yield a colourless oil (56 mg, 56%).

R_f = 0.1 (20% Et_2O in pentane)

^{1}H NMR (400 MHz, $CDCl_3$) δ: 7.46 (d, *J* = 7.9 Hz, 2H), 7.41 (d, *J* = 8.0 Hz, 2H), 6.63 (t, *J* = 56.6 Hz, 1H), 5.91 (ddt, *J* = 16.6, 10.2, 6.0 Hz, 1H), 5.19 (dq, *J* = 17.2, 1.7 Hz, 1H), 5.12 (dq, *J* = 10.3, 1.4 Hz, 1H), 3.83 (s, 2H), 3.26 (dt, *J* = 6.0, 1.4 Hz, 2H), 1.55 (br. s, 1H).

^{13}C NMR (151 MHz, $CDCl_3$) δ: 143.2 (t, *J* = 2.0 Hz), 136.6, 133.1 (t, *J* = 22.4 Hz), 128.4, 125.7 (t, *J* = 6.0 Hz), 116.2, 114.8 (t, *J* = 238.3 Hz), 52.8, 51.7.

^{19}F NMR (376 MHz, $CDCl_3$) δ: -109.9 (d, *J* = 56.5 Hz, 2F).

HRMS (ESI+) calc: $[M+H]^+$ ($C_{11}H_{14}NF_2$) 198.1089; measured: 198.1085 = 2.0 ppm

difference.

IR (neat) $\tilde{v}_{max}$/ cm^{-1}: 3351, 3085, 2983, 2926, 2190, 1619, 1576.

*1-(Difluoromethyl)-2-methylbenzene, **19b***

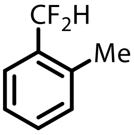

19b was synthesised from 1-(difluoromethyl)-2-methylbenzene following General Procedure A, owing to its volatility a spectroscopic yield is given (64%). Diagnostic NMR signals given below, confirmed through literature comparison.

^{1}H NMR (400 MHz, $CDCl_3$) δ: 6.69 (t, *J* = 55.1 Hz, 1H), 2.35 (s, 3H).

^{19}F NMR (376 MHz, $CDCl_3$) δ: -113.2 (d, *J* = 55.6 Hz, 2F).

Data in accordance with the literature.[19]

*(4-(Difluoromethyl)phenyl)methanol, **20b***

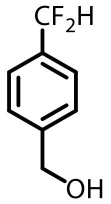

20b was synthesised from (4-(trifluoromethyl)phenyl)methanol following General Procedure A without TMSCl and purified by using silica gel chromatography (50% Et_2O in Pentane to 70% Et_2O in Pentane) to yield a colourless oil (33 mg, 42%).

R_f = 0.2 (50% Et_2O in Pentane)

^{1}H NMR (400 MHz, $CDCl_3$) δ: 7.51 (d, *J* = 8.0 Hz, 2H), 7.46 (d, *J* = 8.1 Hz, 2H), 6.65 (t, *J* = 56.5 Hz, 1H), 4.76 (s, 2H), 4,34 (br s, 1H).

^{13}C NMR (151 MHz, $CDCl_3$) δ: 143.6, 133.6 (t, *J* = 22.4 Hz), 127.0, 125.8 (t, *J* = 6.0 Hz), 114.7 (t, *J* = 238.5 Hz), 64.7.

^{19}F NMR (376 MHz, $CDCl_3$) δ: -110.2 (d, *J* = 56.5 Hz, 2F).

Data in accordance with the literature.[20]

*(3-(Difluoromethyl)phenyl)methanamine, **21b***

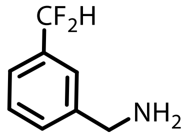

21b was synthesised from *(3-(trifluoromethyl)phenyl)methanamine* following General Procedure A without TMSCl. **21b** was unable to be isolated satisfactorily (by silica chromatography or reverse phase prep-HPLC) despite numerous attempts. Owing to this a spectroscopic yield is given (50%). Diagnostic NMR signals given below, and product identity confirmed through HRMS analysis of the crude reaction mixture.

LCMS (ESI+): t_r = 0.42 min, 158.1 $[M+H]^+$ (Method A).

^{1}H NMR (400 MHz, MeOD) δ: 7.52 – 7.32 (m, 4H), 6.73 (t, *J* = 56.2 Hz, 1H), 4.62 (s, 2H).

^{19}F NMR (376 MHz, MeOD) δ: -112.6 (d, *J* = 56.0 Hz, 2F).

HRMS (ESI+) calc: $[M+H]^+$ ($C_8H_{10}F_2N$) 158.0776; measured: 158.0777 = 0.63 ppm difference.

*2-(4-(Difluoromethyl)phenyl)-4,4,5,5-tetramethyl-1,3,2-dioxaborolane, **22b***

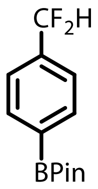

22b was synthesised from 2-(4-(trifluoromethyl)phenyl)-4,4,5,5-tetramethyl-1,3,2-dioxaborolane following General Procedure A and purified by using silica gel chromatography (15% Et_2O in pentane to 30% Et_2O in pentane) to yield a colourless solid (20 mg, 31%).

R_f = 0.4 (20% Et_2O in pentane)

^{1}H NMR (400 MHz, $CDCl_3$) δ: 7.89 (d, *J* = 7.4 Hz, 2H), 7.50 (d, *J* = 7.2 Hz, 2H), 6.65 (t, *J* = 56.4 Hz, 1H), 1.36 (s, 12H).

^{13}C NMR (151 MHz, $CDCl_3$) δ: 136.8 (t, *J* = 22.2 Hz), 135.0, 124.7 (t, *J* = 6.0 Hz), 114.7 (t, *J* = 239.0 Hz), 84.1, 24.9. ipso-Carbon adjacent to B not observed.

^{19}F NMR (376 MHz, $CDCl_3$) δ: -112.6 (d, *J* = 56.4 Hz, 2F).

Data in accordance with the literature.[21]

*4-(Difluoromethyl)benzenesulfonamide, **23b***

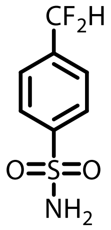

23b was synthesised from 4-trifluoromethyl)benzenesulfonamide following General Procedure A without TMSCl and purified by preparative HPLC to yield a colourless solid (38 mg, 37%).

LCMS (ESI+): t_r = 1.75 min, 208.0 $[M+H]^+$ (Method A).

^{1}H NMR (400 MHz, MeOD) δ: 7.99 (d, *J* = 7.9 Hz, 2H), 7.69 (d, *J* = 7.7 Hz, 2H), 6.84 (t, *J* = 55.8 Hz, 1H).

^{13}C NMR (151 MHz, MeOD) δ: 147.4 (d, *J* = 2.2 Hz), 139.5 (t, *J* = 22.6 Hz), 127.6, 127.4 (t, *J* = 6.1 Hz), 115.4 (t, *J* = 237.8 Hz).

^{19}F NMR (376 MHz, MeOD) δ: -113.6 (d, *J* = 56.3 Hz, 2F).

Data in accordance with the literature.[22]

*Methyl 4-(difluoromethyl)benzoate, **24b***

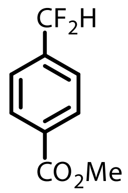

24b was synthesised from methyl 4-(trifluoromethyl)benzoate following General Procedure A however prior to concentration TBAF (1 M in THF) (0.5 mL, 0.5 mmol) was added to the crude mixture in a vial and agitated for 5 minutes. After this the residue was purified by using silica gel chromatography (15% Et_2O in pentane to 30% Et_2O in pentane) to yield a colourless oil (55 mg, 59%).

R_f = 0.4 (20% Et_2O in pentane)

^{1}H NMR (400 MHz, $CDCl_3$) δ: 8.12 (d, *J* = 8.6 Hz, 2H), 7.59 (d, *J* = 8.2 Hz, 2H), 6.69 (t, *J* = 56.1 Hz, 1H), 3.94 (s, 3H).

^{13}C NMR (151 MHz, $CDCl_3$) δ: 166.3, 138.5 (t, *J* = 22.4 Hz), 132.3 (t, *J* = 1.9 Hz), 130.0, 125.7 (t, *J* = 6.0 Hz), 114.0 (t, *J* = 239.8 Hz), 52.4.

^{19}F NMR (376 MHz, $CDCl_3$) δ: -112.1 (d, *J* = 56.2 Hz, 2F).

Data in accordance with the literature.[23]

*Diethyl 4-(difluoromethyl)isophthalate, **25b***

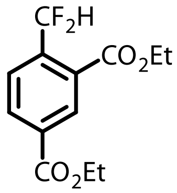

25b was synthesised from **25a** following General Procedure x however prior to concentration TBAF (1 M in THF) (0.5 mL, 0.5 mmol) was added to the crude mixture in a vial and agitated for 5 minutes. After this the residue was purified by using silica gel chromatography (pentane) to yield a colourless oil (65 mg, 36%) isolated alongside *c.a.* 5% remaining **25a** which co elutes, the yield has been adjusted to reflect this.

R_f = 0.3 (5% Et_2O in pentane)

^{1}H NMR (400 MHz, $CDCl_3$) δ: 8.62 (s, 1H), 8.24 (dd, J = 8.2, 1.8 Hz, 1H), 7.85 (d, J = 8.1 Hz, 1H), 7.52 (t, J = 55.2 Hz, 1H), 4.40 (q, J = 7.2 Hz, 4H), 1.40 (td, J = 7.1, 2.5 Hz, 6H).

^{13}C NMR (151 MHz, $CDCl_3$) δ: 165.3, 165.0, 139.0 (t, *J* = 22.4 Hz), 134.6, 133.3, 132.6 (t, *J* = 2.3 Hz), 131.9, 129.6 (t, *J* = 5.1 Hz), 126.3 (t, *J* = 8.1 Hz), 111.6 (t, *J* = 238.6 Hz), 62.0, 61.7, 14.3, 14.2.

^{19}F NMR (376 MHz, $CDCl_3$) δ: -114.4 (d, *J* = 55.2 Hz, 2F).

HRMS (EI+) calc: $[M]^+$ ($C_{13}H_{14}O_4F_2$) 272.0855; measured: 272.0857 = 0.74 ppm

difference.

IR (neat) $\tilde{v}_{max}$/ cm^{-1}: 2985, 2908, 1720, 1368, 1234, 1019, 761.

2-(Difluoromethyl)-5-methoxy-1,3-dimethyl-1H-indole, ***26b***

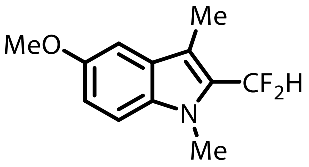

26b was synthesised from **26a** following General Procedure A and purified by using silica gel chromatography (pentane) to yield a colourless solid (26 mg, 23%).

R_f = 0.3 (5% Et_2O in pentane)

^{1}H NMR (400 MHz, $CDCl_3$) δ: 7.37 – 7.18 (m, 1H), 7.04 – 7.01 (m, 2H), 6.96 (t, *J* = 52.9 Hz, 1H), 3.91 (s, 3H), 3.86 (s, 3H), 2.40 (t, *J* = 2.4 Hz, 3H).

^{13}C NMR (151 MHz, $CDCl_3$) δ: 154.0, 133.3, 127.2, 126.9 (t, *J* = 21.0 Hz), 114.9 (d, *J* = 1.3 Hz), 112.9 (t, *J* = 8.0 Hz), 110.4 (t, *J* = 231.9 Hz), 110.3, 101.1, 55.9, 31.0 (t, *J* = 3.1 Hz), 8.4.

^{19}F NMR (376 MHz, $CDCl_3$) δ: -109.4 (d, *J* = 52.9 Hz, 2F).

HRMS (EI+) calc: $[M]^+$ ($C_{12}H_{13}ONF_2$) 225.0960; measured: 225.0957 = 1.3 ppm

difference.

IR (neat) $\tilde{\nu}_{max}$/ cm^{-1}: 2989, 2923, 1625, 1491, 1167, 946.

3-(4-(Difluoromethyl)phenoxy)-N-methyl-3-phenylpropan-1-amine, ***27b***

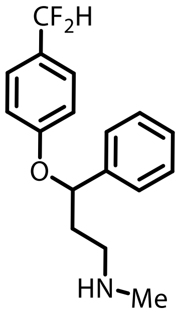

27b was synthesised from *3-(4-trifluoromethyl)phenoxy)-N-methyl-3-phenylpropan-1-amine (Fluoxetine free base)* following General Procedure A without TMSCl and purified by preparative HPLC to yield a colourless oil (60 mg, 41%).

LCMS (ESI+): t_r = 2.51 min, 292.2 $[M+H]^+$ (Method A).

^{1}H NMR (400 MHz, $CDCl_3$) δ: 7.36 – 7.29 (m, 6H), 7.28 – 7.23 (m, 1H), 6.93 – 6.86 (m, 2H), 6.52 (t, *J* = 56.7 Hz, 1H), 5.29 (dd, *J* = 8.3, 4.7 Hz, 1H), 2.82 – 2.70 (m, 2H), 2.44 (s, 3H), 2.20 (dddd, *J* = 14.2, 8.2, 7.2, 6.0 Hz, 1H), 2.09 – 1.98 (m, 1H), 1.90 (s, 1H).

^{13}C NMR (151 MHz, $CDCl_3$) δ: 159.9 (t, *J* = 1.9 Hz), 141.3, 128.7, 127.8, 127.0 (t, *J* = 5.9 Hz), 126.7 (t, *J* = 22.7 Hz), 125.8, 115.9, 114.8 (t, *J* = 237.5 Hz), 78.6, 48.3, 38.6, 36.4.

^{19}F NMR (376 MHz, $CDCl_3$) δ: -109.6 (d, *J* = 56.8 Hz, 2F).

HRMS (ESI+) calc: $[M+H]^+$ ($C_{17}H_{20}NOF_2$) 292.1507; measured: 292.1500 = 2.4 ppm

difference.

IR (neat) $\tilde{\nu}_{max}$/ cm^{-1}: 2942, 1614, 1515, 1382, 1175, 701.

Methyl-(R)-2-((tert-butoxycarbonyl)amino)-3-(4-(difluoromethyl)phenyl)propanoate, ***28b***

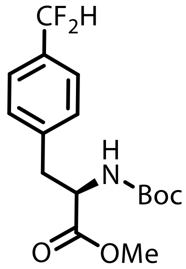

28b was synthesised from **28a** following General Procedure A and purified by using silica gel chromatography (25% Et_2O in pentane to 50% Et_2O in pentane) to yield a colourless solid (87 mg, 49%).

R$_F$ = 0.2 (10% Et_2O in pentane)

^{1}H NMR (400 MHz, $CDCl_3$) δ: 7.44 (d, *J* = 7.8 Hz, 2H), 7.22 (d, *J* = 7.8 Hz, 2H), 6.62 (t, *J* = 56.5 Hz, 1H), 4.99 (d, *J* = 8.5 Hz, 1H), 4.61 (d, *J* = 7.4 Hz, 1H), 3.72 (s, 3H), 3.18 (dd, *J* = 13.8, 5.8 Hz, 1H), 3.07 (dd, *J* = 13.8, 6.4 Hz, 1H), 1.41 (s, 9H).

^{13}C NMR (151 MHz, $CDCl_3$) δ: 172.2, 155.1, 139.2 (t, *J* = 1.9 Hz), 133.3 (t, *J* = 22.4 Hz), 129.8, 125.9 (t, *J* = 6.0 Hz), 114.8 (t, *J* = 238.5 Hz), 80.2, 54.4, 52.5, 38.4, 28.4.

^{19}F NMR (376 MHz, $CDCl_3$) δ: -110.7 (d, *J* = 56.3 Hz, 2F).

Spectral data in accordance with literature.[20]

*Ethyl-(R)-2-((tert-butoxycarbonyl)amino)-3-(4-(difluoromethyl)phenyl)propanoate, **29b***

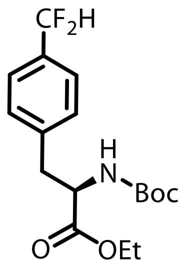

29b was synthesised from **29a** following General Procedure A and purified by using silica gel chromatography (25% Et_2O in pentane to 45% Et_2O in pentane) to yield a colourless solid (99 mg, 52%) alongside *c.a.* 6% Ar-CFH_2 which was inseparable, the yield has been adjusted to reflect this.

R_f = 0.2 (20% Et_2O in pentane)

^{1}H NMR (400 MHz, $CDCl_3$) δ: 7.43 (d, *J* = 8.1 Hz, 2H), 7.21 (d, *J* = 7.9 Hz, 2H), 6.62 (t, *J* = 56.6 Hz, 1H), 4.40 – 4.19 (m, 1H), 4.18 – 4.06 (m, 1H), 3.84 – 3.71 (m, 1H), 3.56 – 3.27 (m, 2H), 1.48 (s, 9H), 1.26 (t, *J* = 7.1 Hz, 3H).

^{13}C NMR (151 MHz, $CDCl_3$) δ: 172.1, 156.9, 142.3, 132.7 (t, *J* = 22.4 Hz), 130.0, 125.6 (t, *J* = 6.0 Hz), 114.8 (t, *J* = 238.3 Hz), 80.7, 61.1, 59.0, 35.3, 28.3, 14.1.

^{19}F NMR (376 MHz, $CDCl_3$) δ: -109.9 (d, *J* = 56.6 Hz, 2F).

HRMS (ESI+) calc: $[M\text{-}Boc+H]^+$ ($C_{12}H_{16}F_2NO_2$) 244.1144; measured: 244.1137 = 2.9 ppm difference.

IR (neat) $\tilde{v}_{max}$/ cm^{-1}: 3365, 2980, 2934, 1709, 1699, 1367, 1161.

(S)-3-(3-(Difluoromethyl)phenyl)-N-(1-(naphthalen-1-yl)ethyl)propan-1-amine, ***30b***

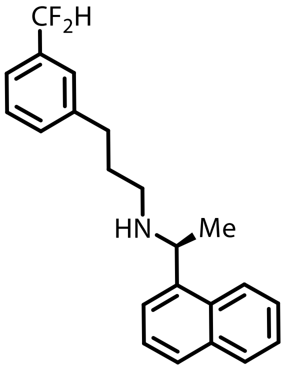

30b was synthesised from *(S)-3-(3-(trifluoromethyl)phenyl)-N-(1-(naphthalen-1-yl)ethyl)propan-1-amine (Cinacalcet free base)* following General Procedure A without TMSCl and purified by preparative HPLC to yield a yellow oil (66 mg, 39%).

LCMS (ESI+): t_r = 2.93 min, 340.2 $[M+H]^+$ (Method A).

^{1}H NMR (400 MHz, $CDCl_3$) δ: 8.22 (d, *J* = 8.3 Hz, 1H), 7.90 (dd, *J* = 7.9, 1.7 Hz, 1H), 7.78 (d, *J* = 8.1 Hz, 1H), 7.67 (d, *J* = 6.1 Hz, 1H), 7.57 – 7.44 (m, 3H), 7.37 – 7.27 (m, 4H), 6.60 (t, *J* = 56.6 Hz, 1H), 4.65 (q, *J* = 6.6 Hz, 1H), 2.78 – 2.57 (m, 4H), 1.86 (p, *J* = 7.4 Hz, 2H), 1.52 (d, *J* = 6.6 Hz, 3H).

^{13}C NMR (151 MHz, $CDCl_3$) δ: 142.9, 141.2, 134.4 (t, *J* = 22.1 Hz), 134.0, 131.3, 130.8 (t, *J* = 2.0 Hz), 129.0, 128.7, 127.2, 125.8, 125.7, 125.4 (t, *J* = 6.0 Hz), 125.3, 123.0 (t, *J* = 6.1 Hz), 123.0, 122.7, 114.9 (t, *J* = 238.5 Hz), 53.8, 47.4, 33.5, 31.9, 23.6.

^{19}F NMR (376 MHz, $CDCl_3$) δ: -111.5 (d, *J* = 56.4 Hz, 2F).

HRMS (ESI+) calc: $[M+H]^+$ ($C_{22}H_{24}F_2N$) 340.1867; measured: 340.1869 = 0.58 ppm

difference.

IR (neat) $\tilde{v}_{max}$/ cm^{-1}: 2942, 1614, 1515, 1382, 1175, 701.

*(1R,5S)-3-Butyl-1-(4-(diifluoromethyl)phenyl)-3-azabicyclo[3.1.0]hexane, **31b***

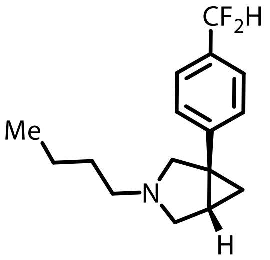

31b was synthesised from **31a** following General Procedure A and purified by using silica gel chromatography (15% Et_2O in pentane to 30% Et_2O in pentane) to yield a colourless oil (64 mg, 45%), the yield has been correct to reflect 8% remaining **31a** that was inseparable, the yield has been adjusted to reflect this.

R_f = 0.3 (20% Et_2O in pentane)

^{1}H NMR (400 MHz, $CDCl_3$) δ: 7.42 (d, *J* = 8.5 Hz, 2H), 7.21 (d, *J* = 7.6 Hz, 2H), 6.62 (t, *J* = 56.6 Hz, 1H), 3.37 (d, *J* = 8.3 Hz, 1H), 3.13 (d, *J* = 8.6 Hz, 1H), 2.56 (d, *J* = 8.4 Hz, 1H), 2.52 – 2.41 (m, 3H), 1.76 – 1.70 (m, 1H), 1.58 – 1.41 (m, 3H), 1.36 (dq, *J* = 8.2, 6.9 Hz, 2H), 0.93 (t, *J* = 7.3 Hz, 3H), 0.82 (dd, *J* = 8.1, 4.3 Hz, 1H).

^{13}C NMR (151 MHz, $CDCl_3$) δ: 146.4 (t, *J* = 2.0 Hz), 131.7 (t, *J* = 22.4 Hz), 126.4, 125.5 (t, *J* = 5.9 Hz), 114.8 (t, *J* = 238.1 Hz), 58.6, 55.4, 55.3, 30.9, 25.4, 20.6, 17.8, 14.1.

^{19}F NMR (376 MHz, $CDCl_3$) δ: -109.6 (d, *J* = 56.6 Hz, 2F).

HRMS (ESI+) calc: $[M+H]^+$ ($C_{16}H_{22}NF_2$) 266.1715; measured: 266.1708 = 2.6 ppm

difference.

IR (neat) $\tilde{\nu}_{max}$/ cm^{-1}: 2957, 2930, 2791, 1618, 1326, 1071.

(3aR,5R,6S,6aR)-5-((R)-2,2-Dimethyl-1,3-dioxolan-4-yl)-2,2-dimethyltetrahydrofuro[2,3-d][1,3]dioxol-6-yl 4-(difluoromethyl)benzoate, ***32b***

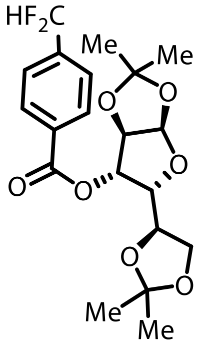

32b was synthesised from **32a** following General Procedure A however prior to concentration TBAF (1 M in THF) (0.5 mL, 0.5 mmol) was added to the crude mixture in a vial and agitated for 5 minutes. After this the residue was purified by using silica gel chromatography (25% Et_2O in pentane to 50% Et_2O in pentane) two times to yield a colourless solid (58 mg, 28%).

$\mathbf{R_f}$ = 0.3 (20% Et_2O in pentane)

^{1}H NMR (400 MHz, $CDCl_3$) δ: 8.11 (dd, *J* = 7.9, 0.9 Hz, 2H), 7.69 – 7.57 (m, 2H), 6.69 (t, *J* = 56.1 Hz, 1H), 5.95 (d, *J* = 3.7 Hz, 1H), 5.51 (d, *J* = 2.8 Hz, 1H), 4.63 (d, *J* = 3.7 Hz, 1H), 4.37 – 4.30 (m, 2H), 4.15 – 4.03 (m, 2H), 1.55 (s, 3H), 1.41 (s, 3H), 1.32 (s, 3H), 1.25 (s, 3H).

^{13}C NMR (151 MHz, $CDCl_3$) δ: 164.4, 139.0 (t, *J* = 22.5 Hz), 131.7 (t, *J* = 1.8 Hz), 130.1, 125.8 (t, *J* = 6.0 Hz), 113.8 (t, *J* = 240.0 Hz), 112.5, 109.5, 105.1, 83.4, 80.0, 77.0, 72.5, 67.4, 26.8, 26.7, 26.2, 25.2.

^{19}F NMR (376 MHz, $CDCl_3$) δ: -112.6 (d, *J* = 55.9 Hz, 2F).

Data in accordance with the literature.[20]

3-(2-(Difluoromethyl)-10H-phenothiazin-10-yl)-N,N-dimethylpropan-1-amine, ***33b***

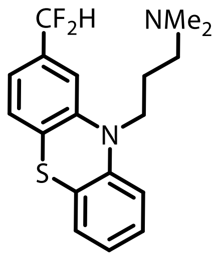

33b was synthesised from 3-(2-(trifluoromethyl)-10H-phenothiazin-10-yl)-N,N-dimethylpropan-1-amine (Triflupromazine free base) following General Procedure A without TMSCl and purified preparative HPLC to yield a colourless oil (43 mg, 26%).

LCMS (ESI+): t_r = 2.75 min, 335.1 $[M+H]^+$ (Method A).

^{1}H NMR (400 MHz, $CDCl_3$) δ: 7.21 – 7.11 (m, 3H), 7.05 – 6.84 (m, 4H), 6.58 (t, *J* = 56.6 Hz, 1H), 3.94 (dd, *J* = 7.7, 6.4 Hz, 2H), 2.44 – 2.35 (m, 2H), 2.21 (s, 6H), 2.01 – 1.90 (m, 2H).

^{13}C NMR (151 MHz, $CDCl_3$) δ: 145.8, 144.7, 137.2, 133.5 (t, *J* = 22.3 Hz), 128.5 (t, *J* = 2.2 Hz), 127.5, 127.5, 124.4, 122.9, 119.6 (t, *J* = 6.3 Hz), 115.8, 114.6, 112.2 (t, *J* = 5.9 Hz), 57.1, 45.6, 45.5, 25.1.

^{19}F NMR (376 MHz, $CDCl_3$) δ: -111.5 (d, *J* = 56.6 Hz, 2F).

Data in accordance with the literature.[24]

Characterisation of Ar-CFH_2 products

*1-Fluoro-4-(fluoromethyl)benzene, **1c***

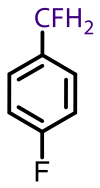

1c was synthesised from 4-fluoro-(trifluoromethyl)benzene following General Procedure C, owing to its volatility a spectroscopic yield is given (43%). Diagnostic NMR signals given below, confirmed through comparison with authentic material synthesised chemically.

^{1}H NMR (400 MHz, $CDCl_3$) δ: 5.34 (d, *J* = 48.0 Hz, 1H).

^{19}F NMR (376 MHz, $CDCl_3$) δ: -114.0 (ddt, *J* = 14.1, 8.6, 5.4 Hz, 1F), -205.1 (t, *J* = 48.3 Hz, 1F).

Data in accordance with the literature.[25]

(Fluoromethyl)benzene), ***2c***

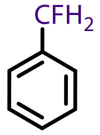

2c was synthesised from (trifluoromethyl)benzene following General Procedure C, owing to its volatility a spectroscopic yield is given (40%). Diagnostic NMR signals given below, confirmed through literature comparison.

^{1}H NMR (400 MHz, $CDCl_3$) δ: 5.40 (d, *J* = 47.9 Hz, 1H).

^{19}F NMR (376 MHz, $CDCl_3$) δ: -205.2 (t, *J* = 47.9 Hz, 1F).

Data in accordance with the literature.[26]

4'-(Fluoromethyl)-2,6-dimethyl-1,1'-biphenyl, ***4c***

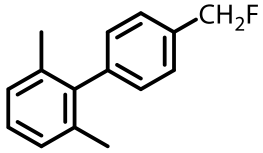

4c was synthesised from **4a** following General Procedure C and purified using silica gel chromatography (pentane) to yield a colourless solid (16 mg, 30 %).

R_f = 0.4 (pentane)

^{1}H NMR (400 MHz, $CDCl_3$) δ: 7.37 (dq, *J* = 8.2, 2.0 Hz, 2H), 7.14 – 7.01 (m, 5H), 5.37 (d, *J* = 47.9 Hz, 2H), 1.95 (s, 6H).

^{13}C NMR (126 MHz, $CDCl_3$) δ: 141.9 (d, *J* = 3.3 Hz), 141.4 (d, *J* = 0.8 Hz), 136.1 (d, *J* = 1.0 Hz), 134.6 (d, *J* = 17.1 Hz), 129.5 (d, *J* = 1.6 Hz), 127.9 (d, *J* = 5.6 Hz), 127.5, 127.3, 84.77 (d, *J* = 165.8 Hz), 20.9.

^{19}F NMR (376 MHz, $CDCl_3$) δ: -205.7 (t, *J* = 48.4 Hz, 1F).

HRMS (EI$^+$) calc: $[M]^+$ ($C_{15}H_{15}F$) 214.1152, measured: 214.1152, 0.0 ppm difference.

IR (neat) νmax/ cm-^{1}: 3023, 2956, 2921, 2853, 1465, 1376, 985, 770

*Ethyl 3-(fluoromethyl)benzoate **5c***

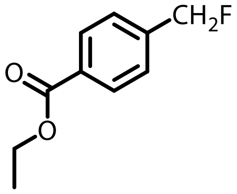

5c was synthesised from ethyl 3-(trifluoromethyl)benzoate following General Procedure C and purified using silica gel chromatography (1% Et_2O in pentane to 10% Et_2O in pentane) to yield a colourless oil (20 mg, 41%).

R_f = 0.5 (20% Et_2O in pentane)

^{1}H NMR (500 MHz, $CDCl_3$) δ: 8.07 (dd, *J* = 8.5, 1.2 Hz, 2H), 7.43 (ddd, *J* = 8.0, 1.5, 0.7 Hz, 2H), 5.45 (d, *J* = 47.2 Hz, 2H), 4.39 (q, *J* = 7.1 Hz, 2H), 1.40 (t, *J* = 7.1 Hz, 3H).

^{13}C NMR (126 MHz, $CDCl_3$) δ: 166.2, 141.0 (d, *J* = 17.2 Hz), 130.7 (d, *J* = 2.4 Hz), 129.8, 126.6 (d, *J* = 6.6 Hz), 83.7 (d, *J* = 168.4 Hz), 61.11, 14.3.

^{19}F NMR (376 MHz, $CDCl_3$) δ: -212.7 (app. tt, *J* = 47.2, 1.4 Hz, 1F).

Spectral data in accordance with literature.[27]

(1-(Fluoromethyl)-4-methoxybenzene), ***6c***

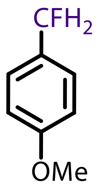

6c was synthesised from 4-methoxy-(trifluoromethyl)benzene following General Procedure C, owing to its volatility a spectroscopic yield is given (27%). Diagnostic NMR signals given below, confirmed by literature comparison.

^{1}H NMR (400 MHz, $CDCl_3$) δ: 5.31 (d, *J* = 48.7 Hz, 1H).

^{19}F NMR (376 MHz, $CDCl_3$) δ: -197.5 (t, *J* = 48.8 Hz, 1F).

Data in accordance with the literature.[28]

*1-(Benzyloxy)-3-(fluoromethyl)benzene, **13c***

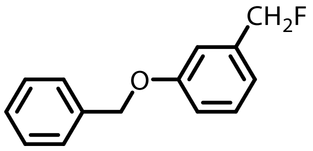

13c was synthesised from 1-(benzyloxy)-3-(trifluoromethyl)benzene following General Procedure C and purified using silica gel chromatography (pentane to 10% Et_2O in pentane) to yield a colourless oil (21.2 mg, 37 %). *Note: **13c** was isolated alongside c.a 6% **13b** that remained as an unreacted intermediate, the yield has been adjusted to reflect this.*

R_f = 0.1 (pentane)

^{1}H NMR (500 MHz, $CDCl_3$) δ: 7.48 – 7.43 (m, 2H), 7.43-7.28 (m, 2H), 7.37 – 7.29 (m, 2H), 7.03 (q, *J* = 1.9 Hz, 1H), 7.01 – 6.92 (m, 2H), 5.37 (d, *J* = 47.7 Hz, 2H), 5.09 (s, 2H).

^{13}C NMR (126 MHz, $CDCl_3$) δ: 159.1 (d, *J* = 1.0 Hz), 137.9 (d, *J* = 17.0 Hz), 136.9, 129.9, 128.7, 128.2, 127.6, 119.9 (d, *J* = 6.1 Hz), 115.3 (d, *J* = 2.8 Hz), 113.8 (d, *J* = 6.3 Hz), 84.5 (d, *J* = 166.9 Hz), 70.2.

^{19}F NMR (376 MHz, $CDCl_3$) δ: 208.0 (t, *J* = 47.2 Hz, 1F).

Spectral data in accordance with literature.[29]

(R)-3-(3-(Fluoromethyl)phenyl)-N-(1-(naphthalen-1-yl)ethyl)propan-1-amine, ***30c***

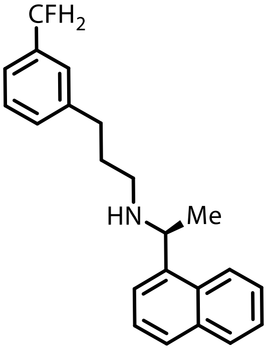

30c was synthesised from (R)-3-(3-(trifluoromethyl)phenyl)-N-(1-(naphthalen-1-yl)ethyl)propan-1-amine (Cinacalcet free base) following General Procedure C and purified using preparative HPLC to yield a yellow oil (13 mg, 16%).

LCMS (ESI+): t_r = 2.77 min, 322.2 $[M+H]^+$ (Method A).

^{1}H NMR (400 MHz, $CDCl_3$) δ: 8.18 (d, *J* = 8.1 Hz, 1H), 7.88 (d, *J* = 7.2 Hz, 1H), 7.76 (d, *J* = 8.1 Hz, 1H), 7.65 (d, *J* = 7.2 Hz, 1H), 7.49 (dt, *J* = 9.9, 5.9 Hz, 3H), 7.22 – 7.09 (m, 3H), 5.32 (d, *J* = 48.0 Hz, 2H), 4.65 (q, *J* = 6.6 Hz, 1H), 2.76 – 2.54 (m, 3H), 1.90 – 1.81 (m, 2H), 1.73 (s, 1H), 1.50 (d, *J* = 6.5 Hz, 2H), 1.00 – 0.76 (m, 3H).

^{13}C NMR (151 MHz, $CDCl_3$) δ: 142.6 (d, *J* = 1.4 Hz), 140.9, 136.2 (d, *J* = 16.9 Hz), 134.0, 131.3, 129.0, 128.8 (d, *J* = 3.1 Hz), 128.6 (d, *J* = 1.4 Hz), 127.6 (d, *J* = 5.7 Hz), 127.3, 125.8, 125.7, 125.4, 125.0 (d, *J* = 5.8 Hz), 122.9, 122.7, 84.7 (d, *J* = 165.9 Hz), 53.7, 47.4, 33.5, 29.7, 23.5.

^{19}F NMR (376 MHz, $CDCl_3$) δ: -208.0 (t, *J* = 48.0 Hz, 1F).

HRMS (ESI+) calc: $[M+H]^+$ ($C_{22}H_{25}NF$) 322.1967; measured: 322.1966 = 0.31 ppm difference.

IR (neat) $\tilde{v}_{max}$/ cm^{-1}: 2959, 2814, 1600, 1490, 1112, 745.

1,2:5,6-Di-O-isopropylidene-3-O-[4-(fluoromethyl)benzoyl]-α-D-glucofuranose, ***32c***

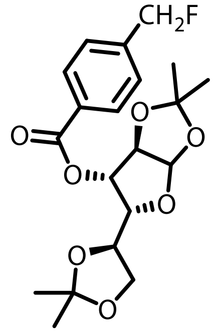

32c was synthesised from **32a** following General Procedure C and purified using silica gel chromatography (5% ethyl acetate in pentane to 30% ethyl acetate in pentane) to yield a colourless solid (39 mg, 39%).

R_f = 0.2 (20% Et_2O in pentane)

^{1}H NMR (500 MHz, $CDCl_3$) δ: 8.05 (d, *J* = 7.4 Hz, 2H), 7.45 (d, *J* = 8.1 Hz, 2H), 5.96 (d, *J* = 3.7 Hz, 1H), , 5.51 (s, 1H), 5.46 (d, *J* = 49.6 Hz, 2H), 4.64 (d, *J* = 3.7 Hz, 1H), 4.41 – 4.29 (m, 2H), 4.15 – 4.05 (m, 2H), 1.56 (s, 3H), 1.41 (s, 3H), 1.32 (s, 3H), 1.26 (s, 3H).

^{13}C NMR (126 MHz, $CDCl_3$) δ: 164.8, 141.9 (d, *J* = 17.3 Hz), 130.0, 129.6 (d, *J* = 2.3 Hz), 126.7 (d, *J* = 6.6 Hz), 112.4, 109.5, 105.2, 83.6 (d, *J* = 169.1 Hz), 83.4, 80.0, 77.2, 72.6, 67.3, 26.8, 26.7, 26.2, 25.2.

^{19}F NMR (283 MHz, $CDCl_3$) δ: -214.7 (t, *J* = 47.0 Hz, 1F).

Spectral data in accordance with literature.[30]

3-(2-(Fluoromethyl)-10H-phenothiazin-10-yl)-N,N-dimethylpropan-1-amine, ***33c***

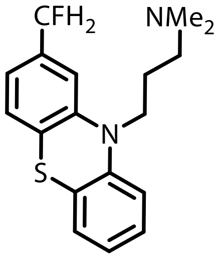

33c was synthesised from 3-(2-(trifluoromethyl)-10H-phenothiazin-10-yl)-N,N-dimethylpropan-1-amine (Triflupromazine free base) following General Procedure C for 20000 s (4 *F*) and purified using preparative HPLC and then trituration with pentane to removal residual electrolyte to yield a yellow oil (29 mg, 37%).

LCMS (ESI+): t_r = 2.73 min, 317.2 $[M+H]^+$ (Method A).

^{1}H NMR (400 MHz, $CDCl_3$) δ: 7.16 – 7.04 (m, 3H), 7.01 – 6.81 (m, 4H), 5.28 (d, *J* = 47.9 Hz, 2H), 3.94 – 3.85 (m, 2H), 2.38 (t, *J* = 7.1 Hz, 2H), 2.18 (s, 6H), 1.92 (p, *J* = 7.1 Hz, 2H).

^{13}C NMR (151 MHz, $CDCl_3$) δ: 145.7 (d, *J* = 1.2 Hz), 145.0, 135.5 (d, *J* = 17.0 Hz), 127.5, 127.5 (d, *J* = 1.4 Hz), 127.4, 126.0 (d, *J* = 3.4 Hz), 124.9, 122.7, 121.6 (d, *J* = 5.9 Hz), 115.7, 114.7 (d, *J* = 6.0 Hz), 84.5 (d, *J* = 166.9 Hz), 57.2, 45.6, 45.5, 25.2

^{19}F NMR (376 MHz, $CDCl_3$) δ: -206.0 (app. tq, *J* = 48.2, 2.1 Hz, 1F).

HRMS (ESI+) calc: $[M+H]^+$ ($C_{18}H_{22}N_2FS$) 317.1482; measured: 317.1480 = 0.63 ppm

difference.

IR (neat) $\tilde{v}_{max}$/ cm^{-1}: 2930, 2856, 2766, 1598, 1566, 1460, 1422, 748.

*4-(Fluoromethyl)-1,1'-biphenyl, **34c***

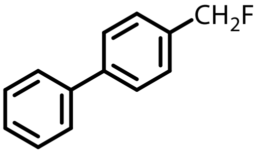

34c was synthesised from **34a** following General Procedure C and purified using silica gel chromatography (pentane) to yield a colourless oil (18 mg, 39 %).

R_f = 0.2 (pentane)

^{1}H NMR (500 MHz, $CDCl_3$) δ: 7.68 – 7.60 (m, 4H), 7.51 – 7.45 (m, 4H), 7.39 (tt, *J* = 7.2, 1.2 Hz, 1H), 5.46 (d, *J* = 48.0 Hz, 2H).

^{13}C NMR (126 MHz, $CDCl_3$) δ: 141.8 (d, *J* = 3.2 Hz), 140.6 (d, *J* = 1.3 Hz), 135.1 (d, *J* = 17.1 Hz), 128.8, 128.1 (d, *J* = 5.7 Hz), 127.6, 127.4 (d, *J* = 1.5 Hz), 127.2 (d, *J* = 0.6 Hz), 84.4 (d, *J* = 166.0 Hz).

^{19}F NMR (376 MHz, $CDCl_3$) δ: -206.3 (t, *J* = 47.8 Hz, 1F).

Spectral data in accordance with literature.[31]

Low-yielding and unsuccessful substrates

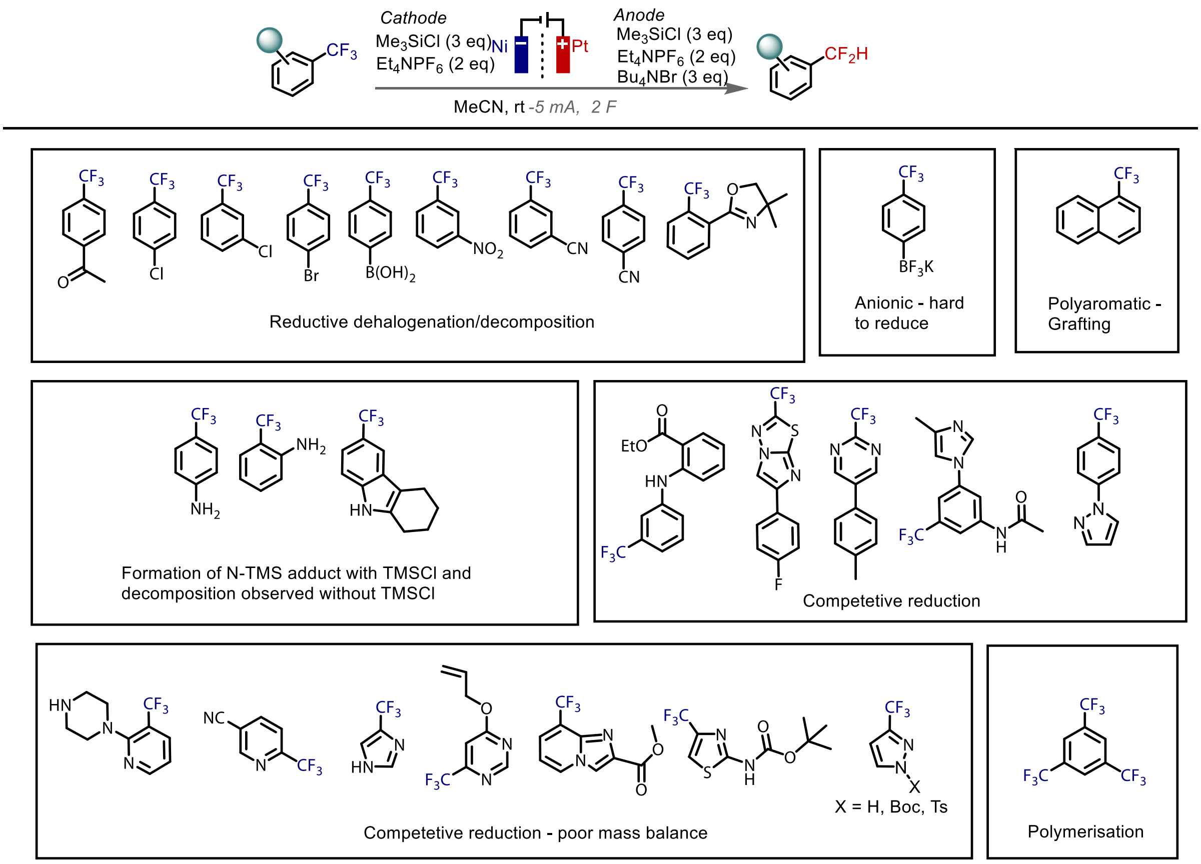

Figure S18. Failed and low yielding substrates.

Mechanistic experiments

Intramolecular alkene trapping

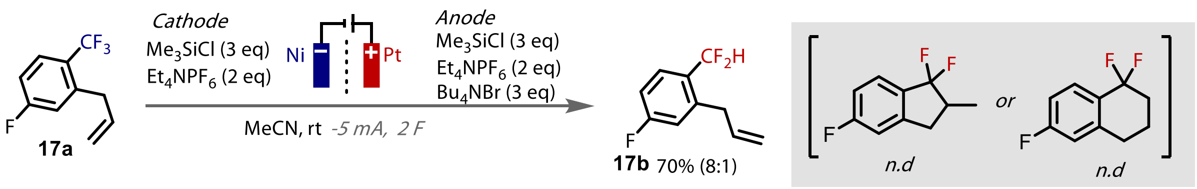

Scheme S6. Intramolecular trap experiment

An oven-dried (180°C) H-type divided cell equipped with a porous glass frit was evacuated and backfilled with N2 three times. $TEAPF_6$ (275 mg, 1 mmol, 2 equiv.) was added to both the cathode and anode compartments. **17a** (102.1 mg, 0.5 mmol, 1 equiv.) was added to the cathode compartment and Bu_4NBr (480 mg, 1.5 mmol, 3 equiv.) was added to the anode compartment. To each compartment was added (with stirring, 900 RPM) degassed, anhydrous MeCN (2.5 mL, 0.2 M) and then TMSCl (0.19 mL, 1.5 mmol, 3 equiv.). Under a strong flow of N2 the septa were replaced with ones containing a Pt wire electrode (entire coil area made of ~ 10 cm Pt wire submerged) as the anode and a Ni foil electrode (submerged surface area 2.5 cm^2). A constant current of -5.0 mA was applied for 19320 s (2 *F*). When the electrolysis was finished, the catholyte was concentrated directly onto silica and purified by flash column chromatography eluting with Pentane to yield a colourless oil (65 mg, 70%).

Intermolecular alkene trapping

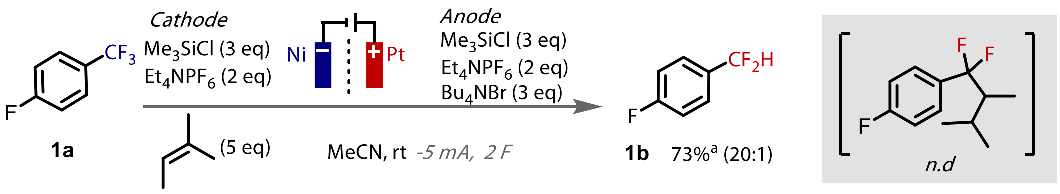

Scheme S7. Intermolecular trap experiment

An oven-dried (180°C) H-type divided cell equipped with a porous glass frit was evacuated and backfilled with N2 three times. $TEAPF_6$ (275 mg, 1 mmol, 2 equiv.) was added to both the cathode and anode compartments. **1a** (62.4 mL, 0.5 mmol, 1 equiv.) was added to the cathode compartment and Bu_4NBr (480 mg, 1.5 mmol, 3 equiv.) was added to the anode compartment. To each compartment was added (with stirring, 900 RPM) degassed, anhydrous MeCN (2.5 mL, 0.2 M) and then TMSCl (0.19 mL, 1.5 mmol, 3 equiv.). Amylene (265 μL, 2.5 mmol, 5 equiv.) was then added to the cathode compartment. Under a strong flow of N2 the septa were replaced with ones containing a Pt wire electrode (entire coil area made of ~ 10 cm Pt wire submerged) as the anode and a Ni foil electrode (submerged surface area 2.5 cm^2). A constant current of -5.0 mA was applied for 19320 s (2 *F*). A spectroscopic yield of **1b** was determined using ^{19}F NMR against an internal C_6F_6 standard.

Product Hammett Analysis

Combined data for the construction of Figure **2b** using our data and that from work reported by Shang, Jui, Gouverneur and Prakash. Products with 0% yield were excluded from this analysis as well as substituents with poorly defined sigma values, e.g., heterocycles.

For each substrate, the total sigma parameter (o+p+m) was calculated using values described by Taft[33] for substituents on the Ar-CF_2H ring.

This work

Number	σ (o/p+m)	yield
	0	80
	0.06	76
	-0.14	68
	0	42
	0.12	65
	-0.27	64
	-0.83	75
	-0.17	64
	0.1	63
	0.45	59
	0.45	58
	0.9	31
	-0.81	58
	0.6	37
	0.12	31
	0	51
	-0.03	50
	0.06	60
	-0.08	70
	0.01	56
	-0.03	58
	-0.72	47
	-0.72	57
	-0.83	36
	-0.24	41
	-0.15	49
	-0.15	39
	-0.2	45
	0.45	28
	-0.09	26

Table S2. Calculated total Hammett values for this work.

Jui (ref 34 in manuscript)[2]

	σ p	σ m	Total	yields
**53**	0		0	75
**54**		0.21	0.21	55
**55**	0		0	70
**56**	-0.05		-0.05	64
**57**		-0.07	-0.07	48
**58**	-0.15		-0.15	50
**59**	-0.66	0.56	-0.1	65
**60**		0.12	0.12	53
**61**		0.39	0.39	45

Table S3. Calculated total Hammett values for work by Jui.

Gouverneur (ref 35 in manuscript)[3]

	yield	σ p	σ m	total
**1b**	65	0.66		0.66
**2b**	72	0.66		0.66
**3b**	93	0.66	0.34	1
**4b**	40	0.45		0.45
**5b**	48	0.66	0.12	0.78
**6b**	34	0.6		0.6
**7b**	69	0.66	0.21	0.87
**8b**	49	0.45		0.45

Table S4. Calculated total Hammett values for work by Gouverneur.

Shang (ref 36 in manuscript)[1]

	σ p	σ m	Total	yields
**63**	0		0	87
**64**		0.21	0.21	70
**65**	0		0	71
**66**	0.12	-0.01	0.12	72
**70**	-0.15	0.26	0.11	63

Table S5. Calculated total Hammett values for work by Shang.

Prakash (ref 31 in manuscript)[4]

All have m-CF_3	**σ m**	**Total**	**yields**
**1**	0.12	0.55	60
**4**	-0.11	0.32	41
**5**	0	0.43	74
**6**	0.08	0.51	60
**7**	-0.08	0.35	60
**8**	-0.08	0.35	63
**9**	-0.08	0.35	41
**10**	-0.08	0.35	71
**11**	-0.08	0.35	71
**12**	-0.08	0.35	56
**13**	0	0.43	60
**14**	-0.08	0.35	76
**15**	-0.07	0.36	74
**16**	0.35	0.78	60
**17**	0.38	0.81	45
**18**	0.06	0.49	72

Table S6. Calculated total Hammett values for work by Prakash.

Ar-CF2H Hydrogen Bonding strength examination

The solute hydrogen-bond (HB) acidity (A) of a variety of Ar-CF_2H and Ar-CFH_2 products was calculated using the method detailed previously by our group.[32][35]

	^{1}H NMR Shift δ / ppm			A Value
	$CDCl_3$	d_6-DMSO	$\Delta\delta$ (d_6-DMSO - $CDCl_3$)	= 0.0065 + 0.133($\Delta\delta$)
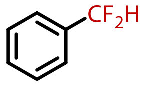	6.6508	7.0309	0.3801	**0.057**
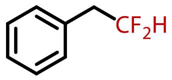	5.9253	6.2236	0.2983	**0.046**
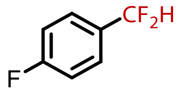	6.6349	7.0419	0.407	**0.061**
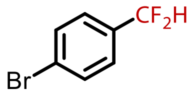	6.6137	7.0454	0.4317	**0.064**
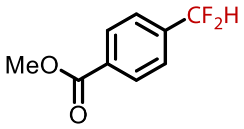	6.6911	7.1422	0.4511	**0.066**
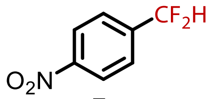	6.7422	7.2182	0.476	**0.070**
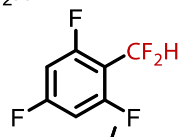	6.8797	7.2698	0.3901	**0.058**
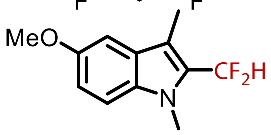	6.956	7.3484	0.3924	**0.059**
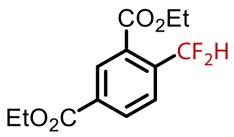	7.5211	7.5785	0.0574	**0.014**
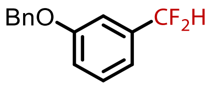	6.6181	6.9852	0.3671	**0.055**
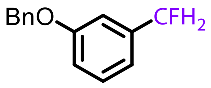	5.37	5.3809	0.0109	**0.008**
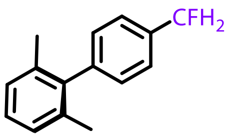	5.37	5.4764	0.1064	**0.021**

Table S8. Spectroscopically determined A values.

NMR Spectra of Novel Substrates

*1-Butyl-3-(trifluoromethyl)-1H-pyrazole, **9a***

^{1}H NMR (400 MHz, DMSO-d_6):

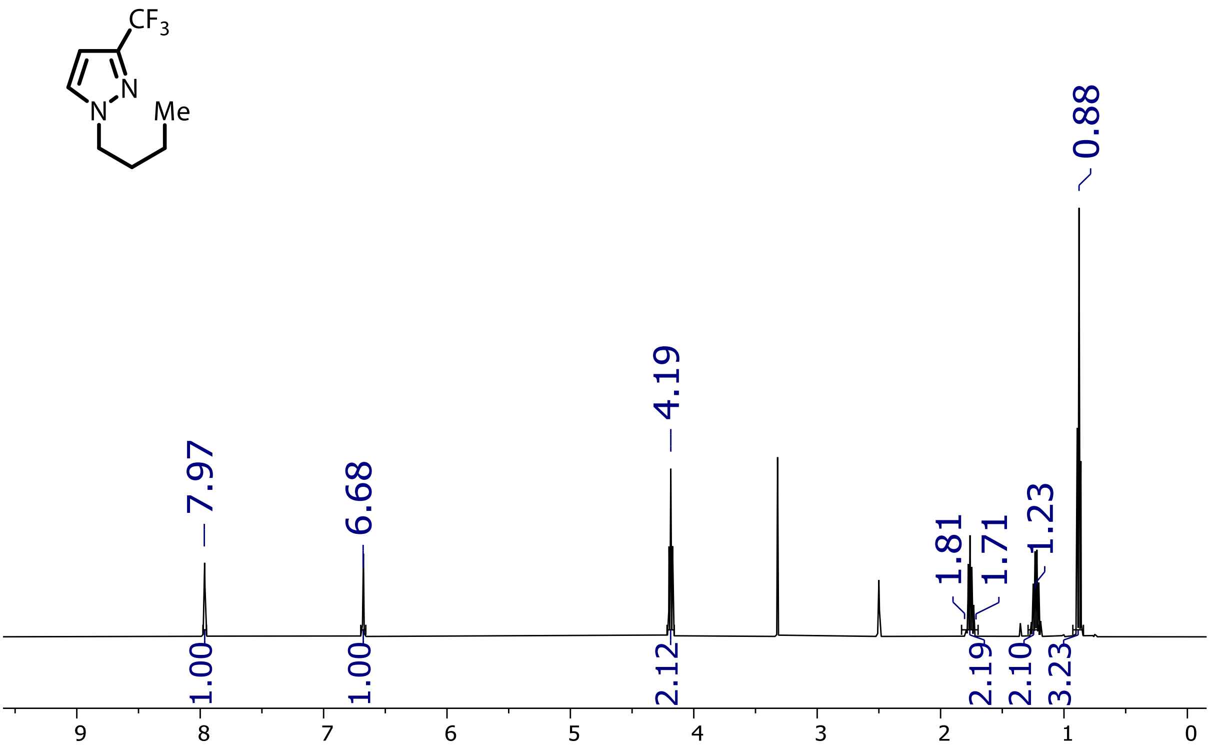

^{13}C NMR (151 MHz, DMSO-d_6):

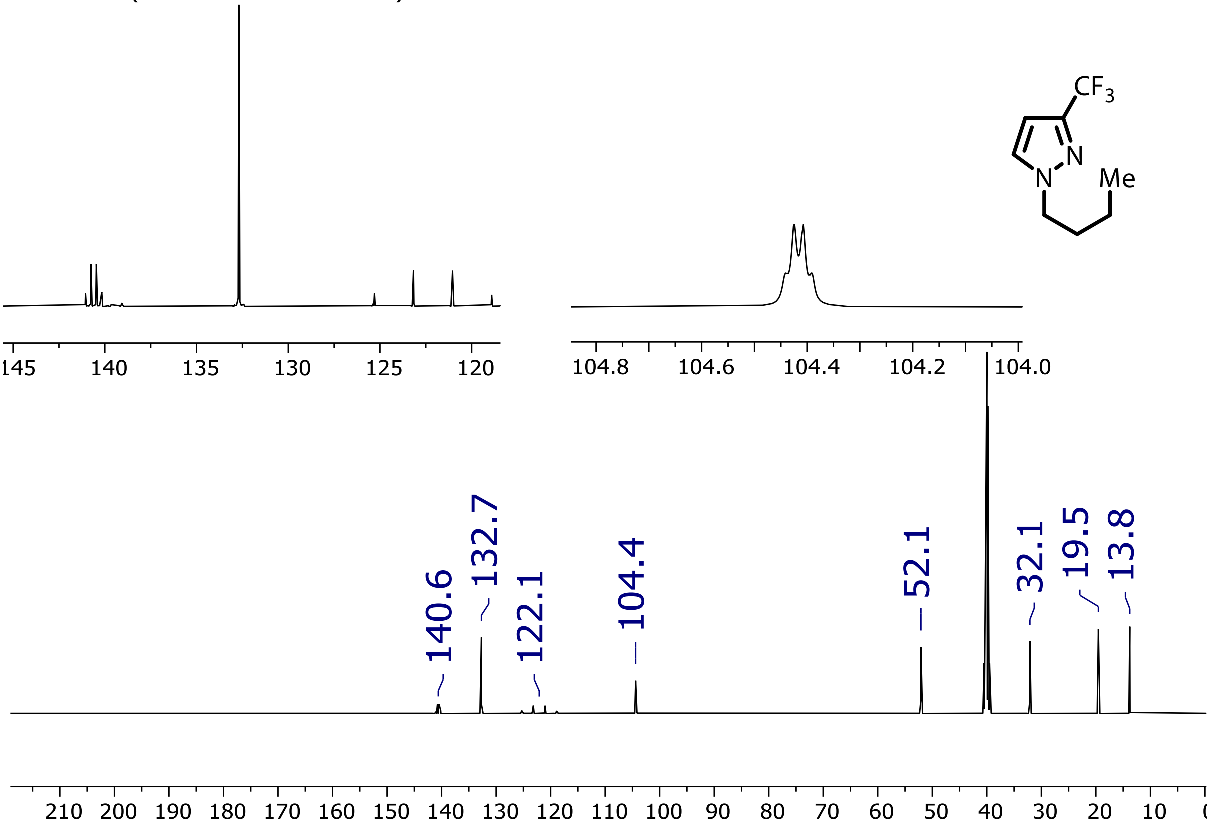

^{19}F NMR (376 MHz, DMSO-d_6):

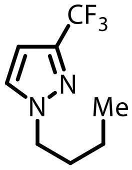

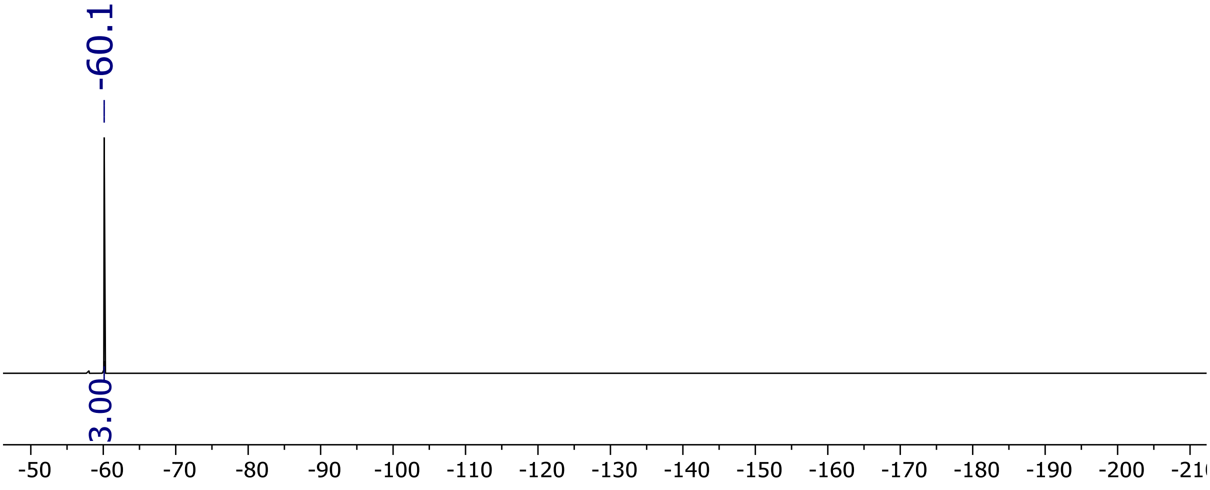

2-Allyl-4-fluoro-1-(trifluoromethyl)benzene, ***17a***

^{1}H NMR (400 MHz, $CDCl_3$):

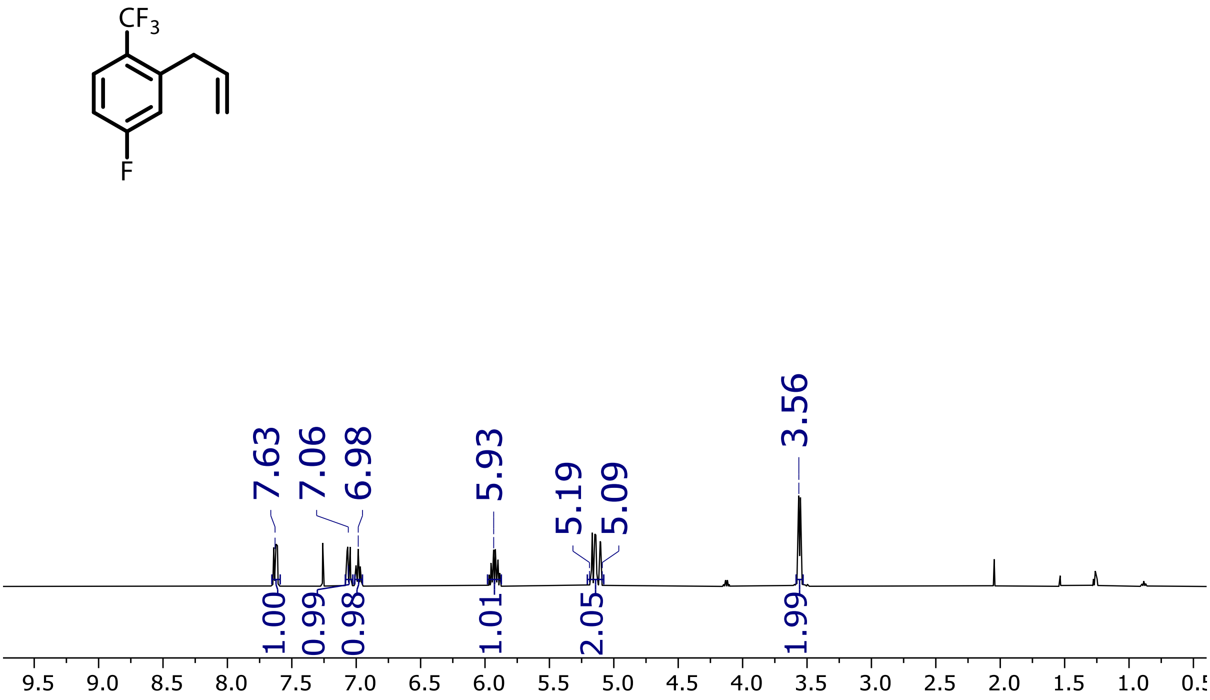

^{13}C NMR (151 MHz, $CDCl_3$):

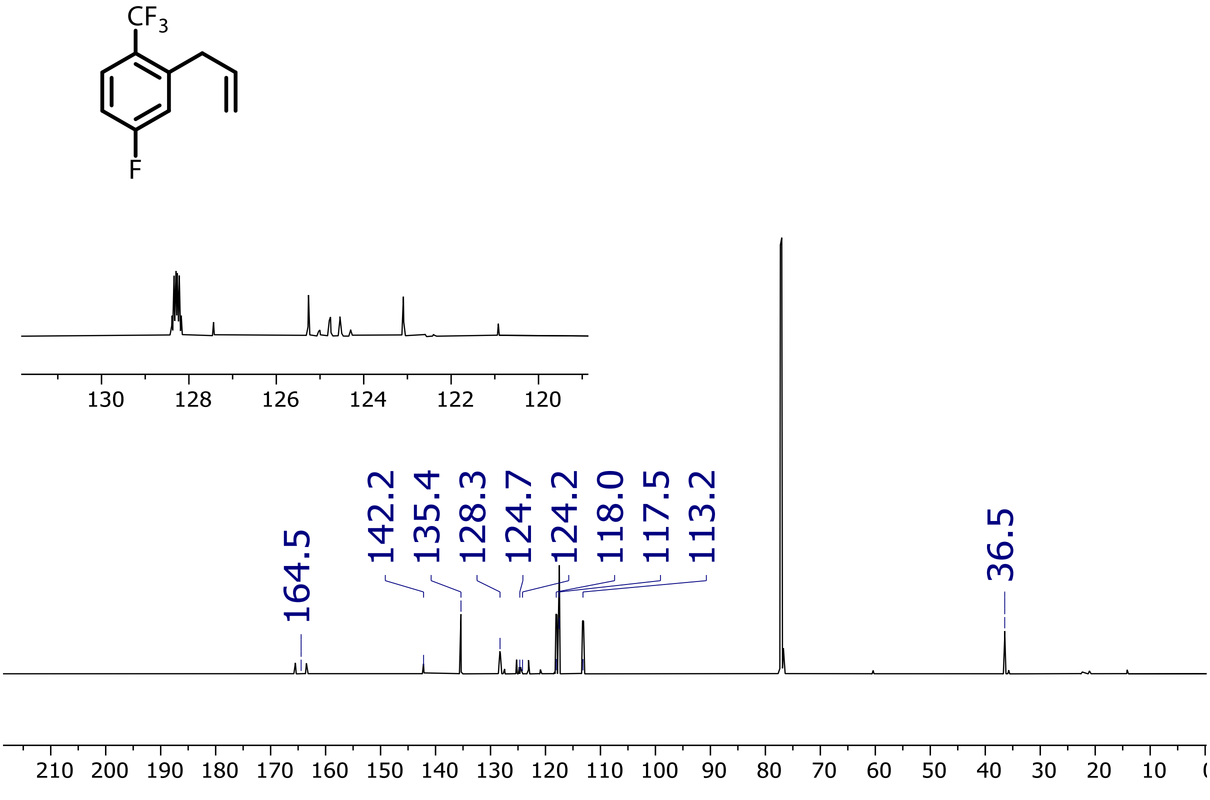

^{19}F NMR (376 MHz, $CDCl_3$):

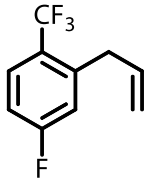

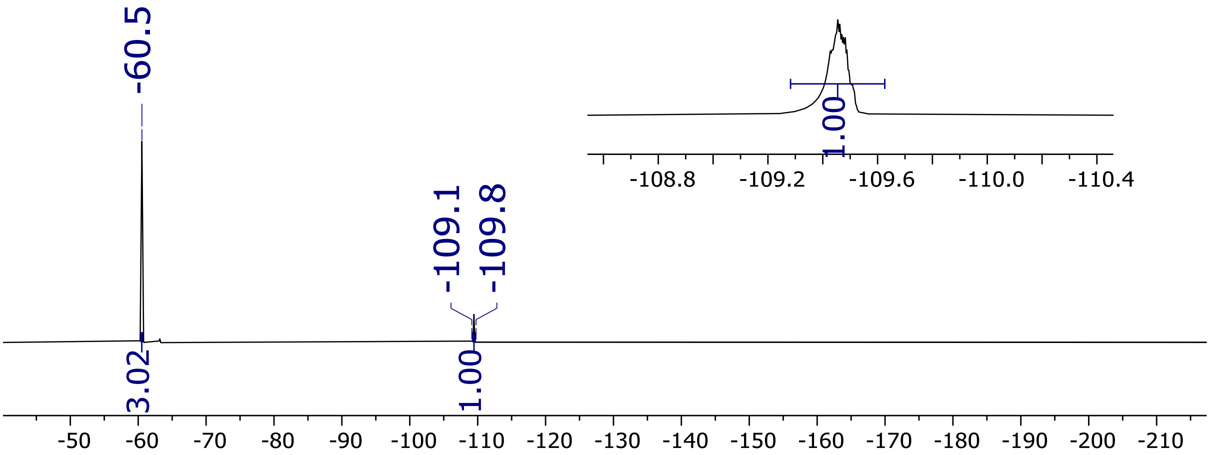

Diethyl 4-(trifluoromethyl)isophthalate, ***25b***

^{1}H NMR (400 MHz, $CDCl_3$):

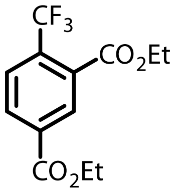

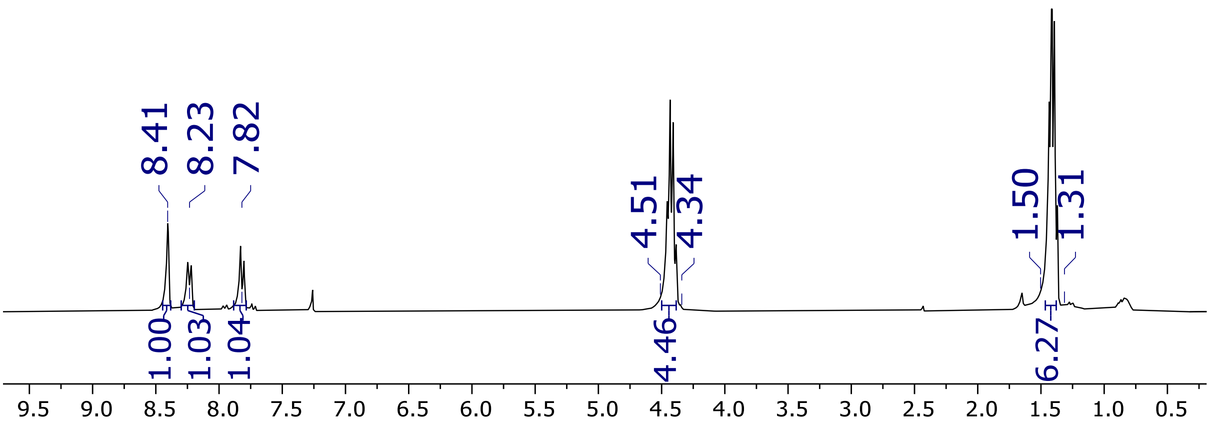

^{13}C NMR (151 MHz, $CDCl_3$):

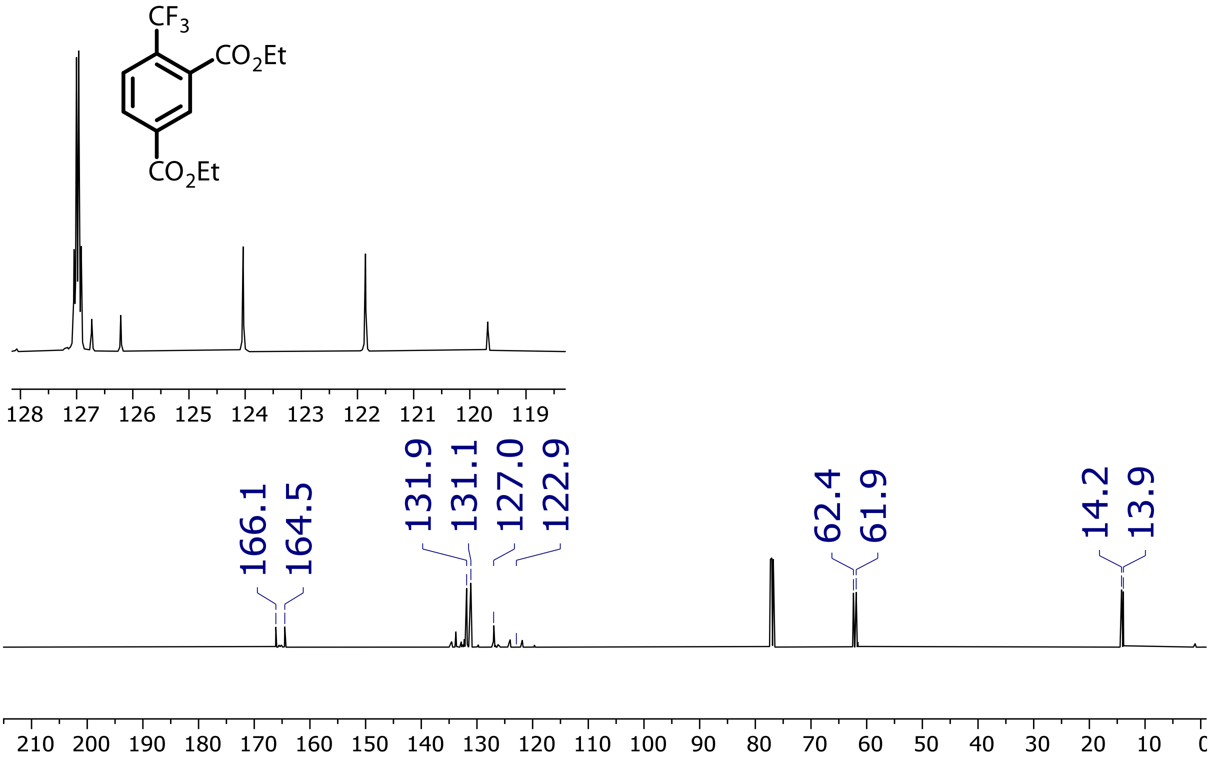

^{19}F NMR (376 MHz, $CDCl_3$):

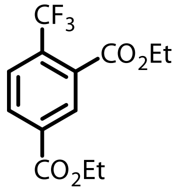

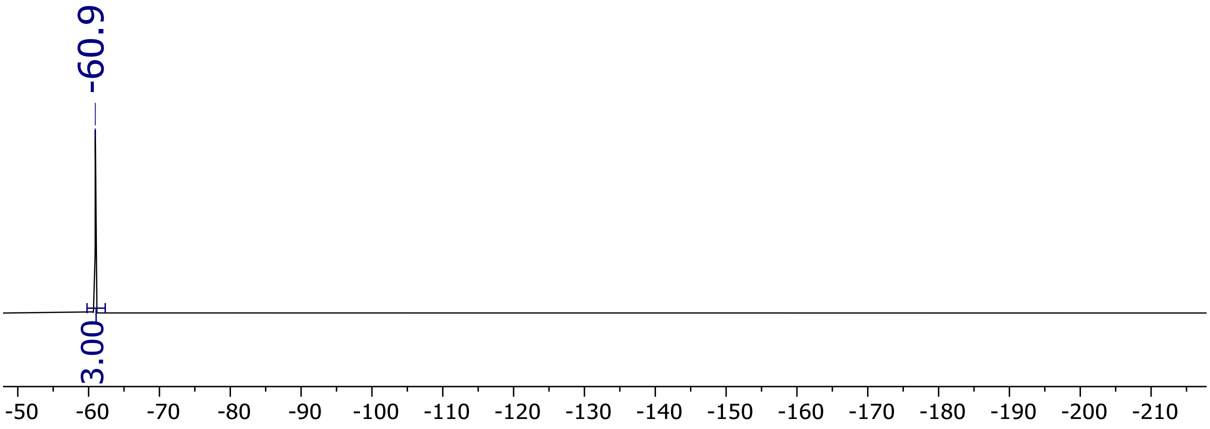

5-Methoxy-1,3-dimethyl-2-(trifluoromethyl)-1H-indole, ***26a***

^{1}H NMR (400 MHz, $CDCl_3$):

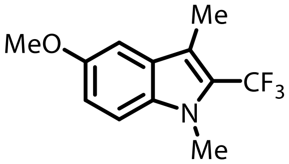

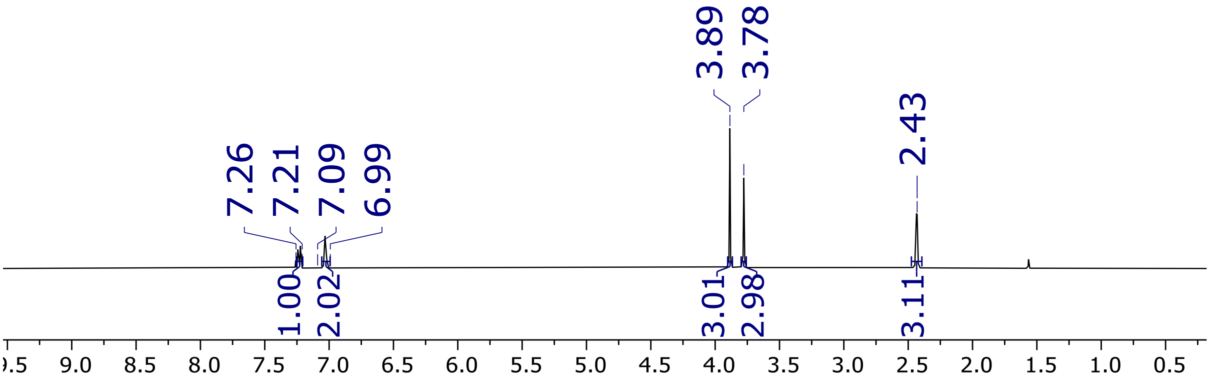

^{13}C NMR (151 MHz, $CDCl_3$):

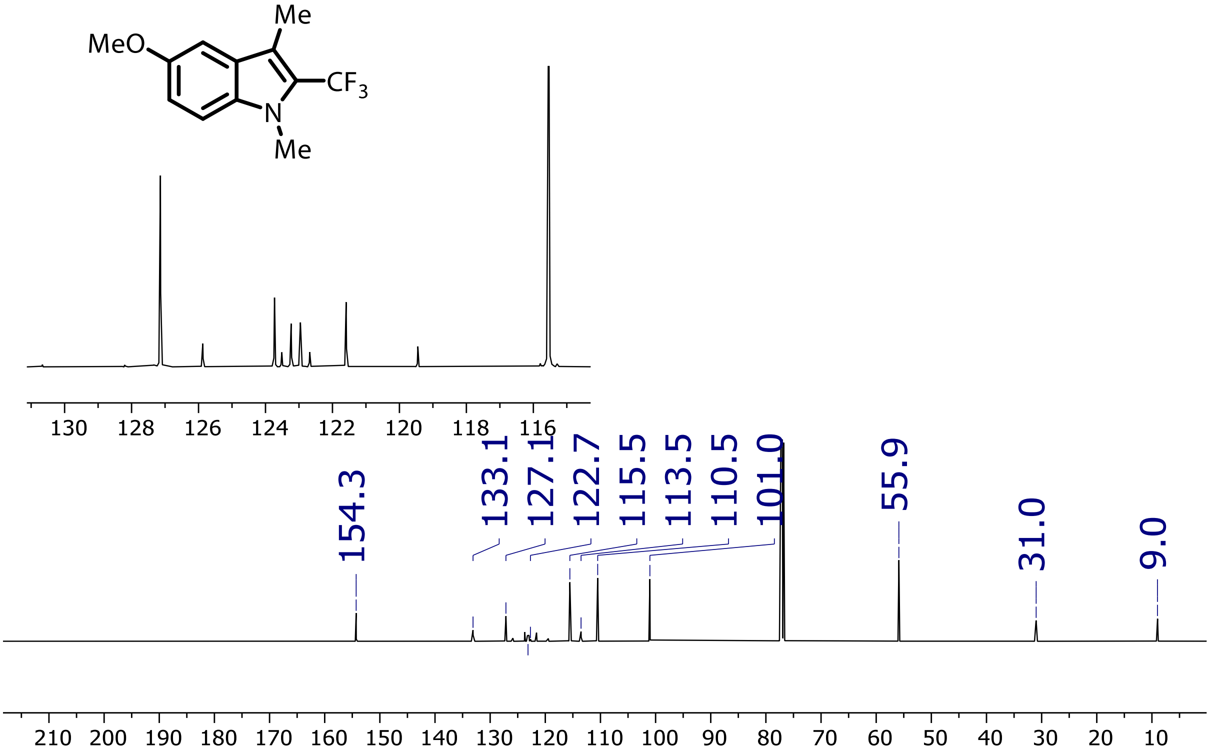

^{19}F NMR (376 MHz, $CDCl_3$):

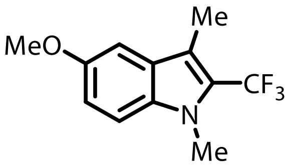

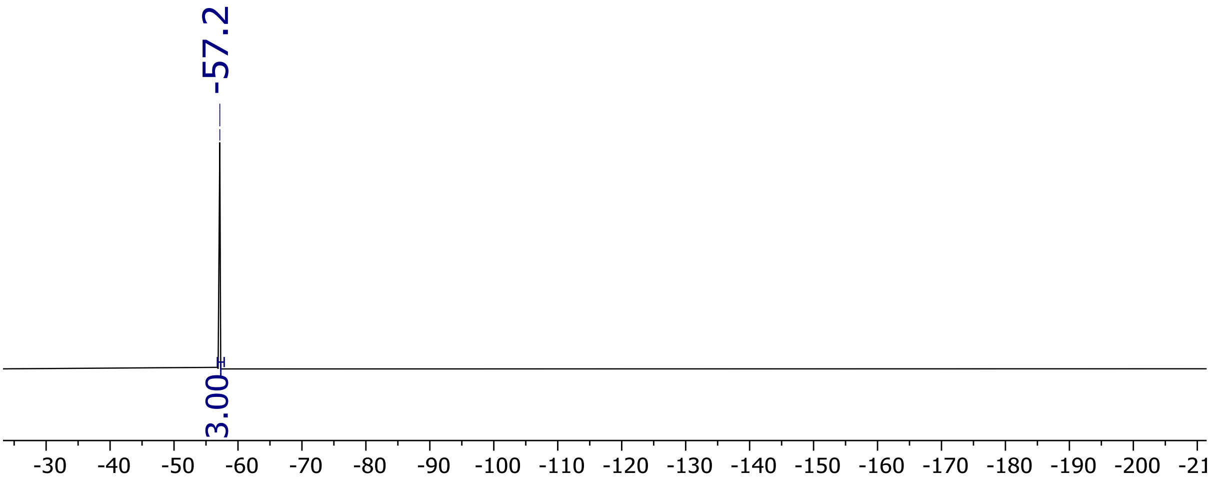

Ethyl-(R)-2-((tert-butoxycarbonyl)amino)-3-(4-(trifluoromethyl)phenyl)propanoate, ***29a***
^{1}H NMR (400 MHz, $CDCl_3$):

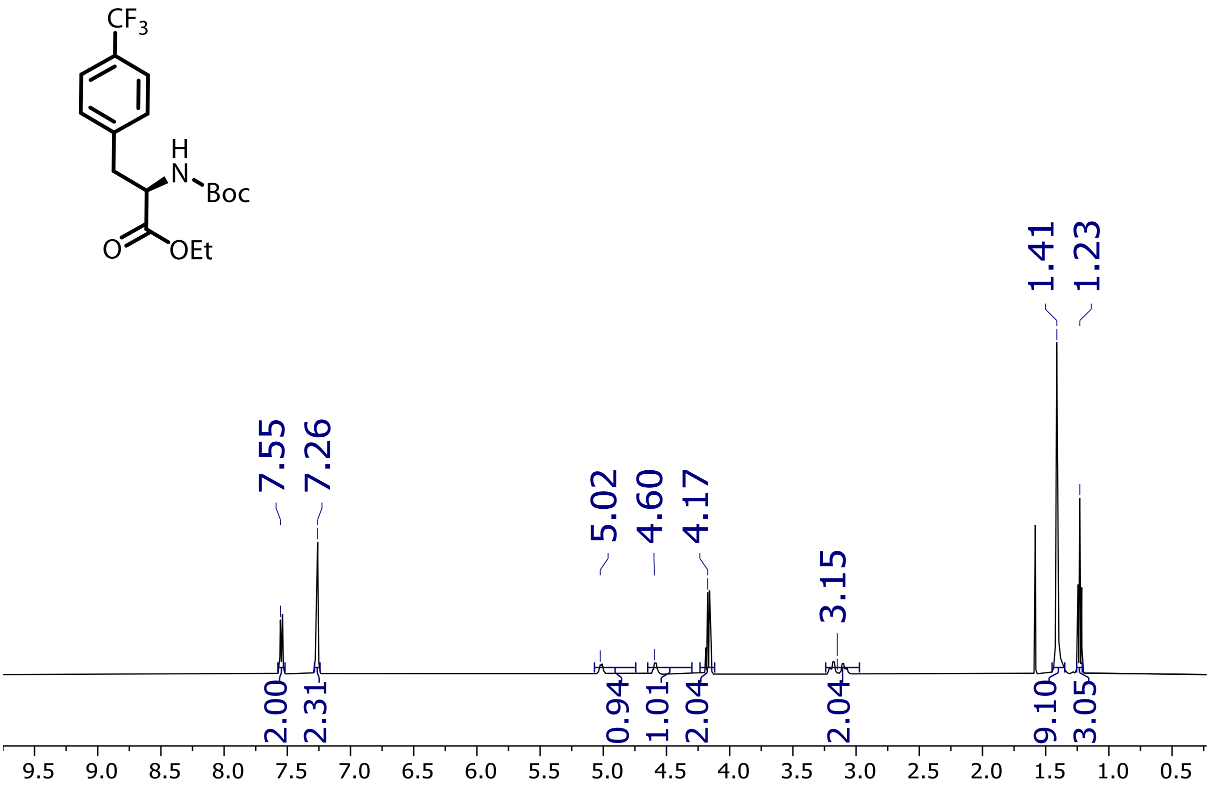

^{13}C NMR (151 MHz, $CDCl_3$):

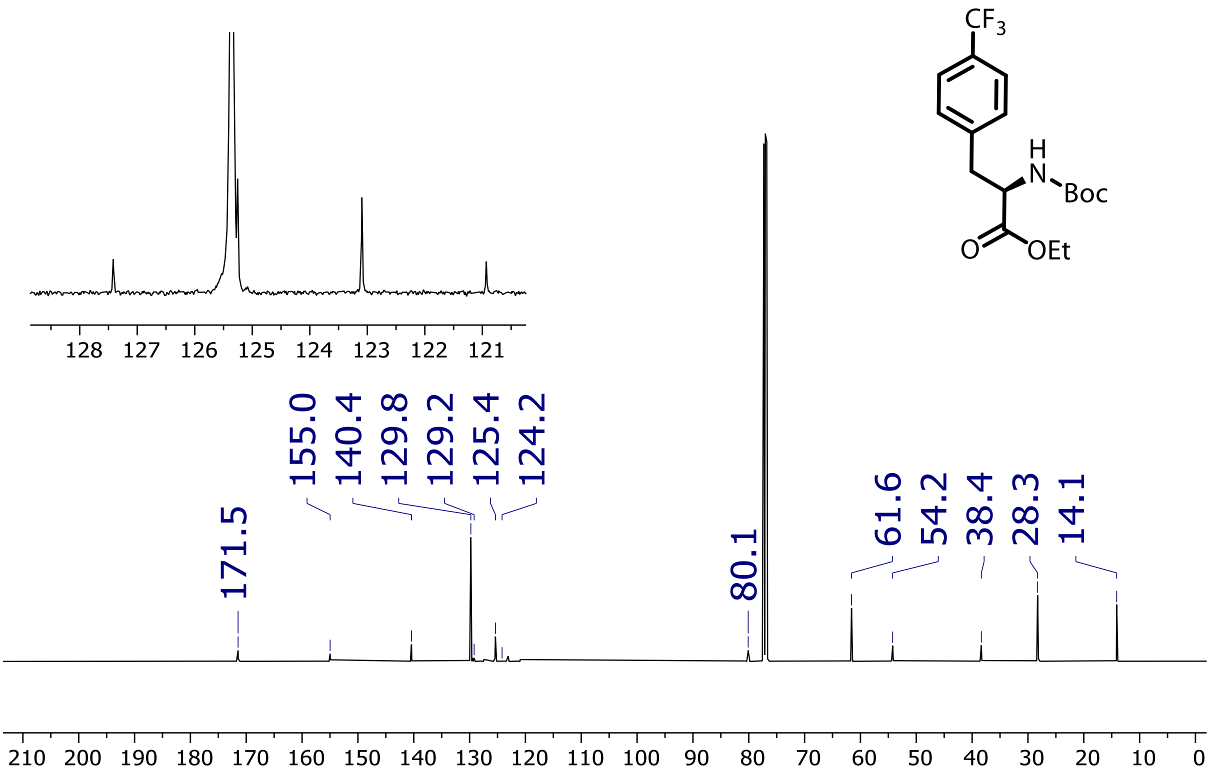

^{19}F NMR (376 MHz, $CDCl_3$):

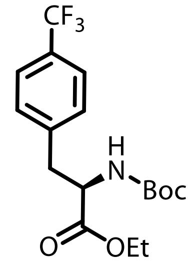

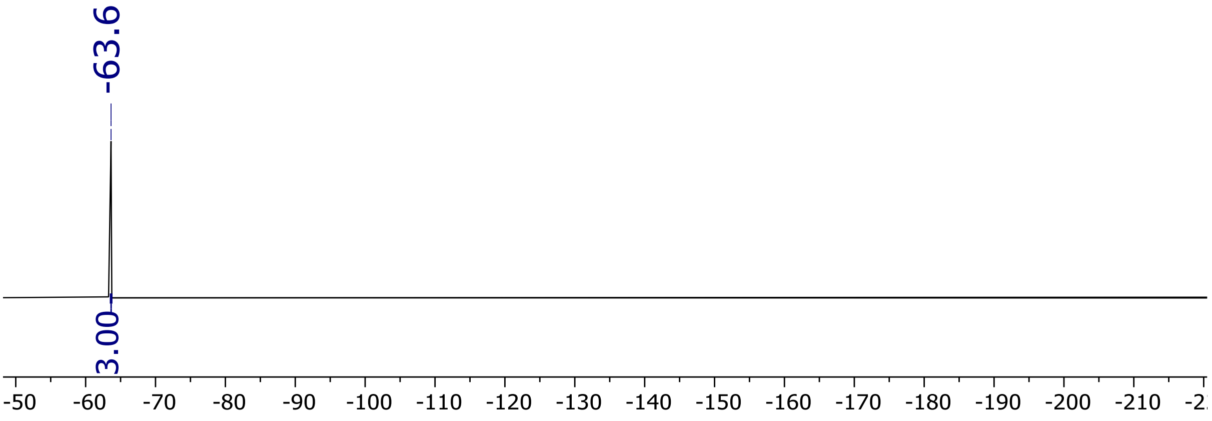

(1R,5S)-3-Butyl-1-(4-(trifluoromethyl)phenyl)-3-azabicyclo[3.1.0]hexane, ***31a***

^{1}H NMR (400 MHz, $CDCl_3$):

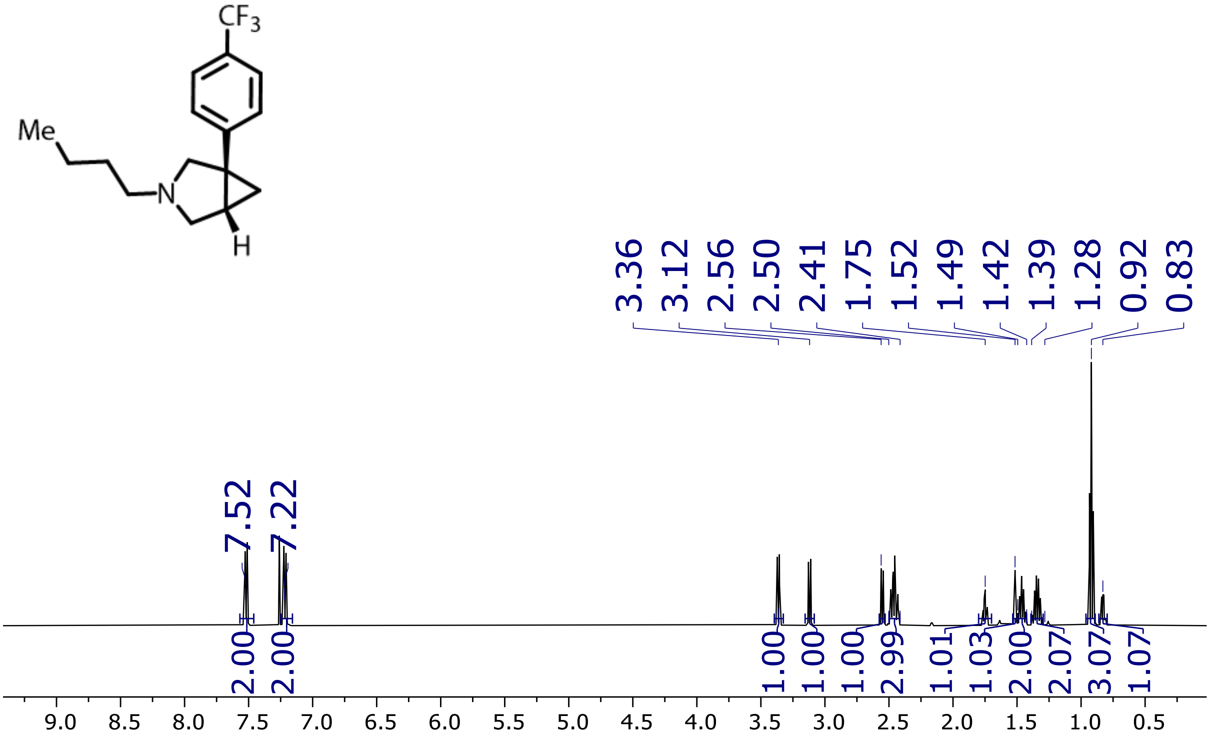

^{13}C NMR (151 MHz, $CDCl_3$):

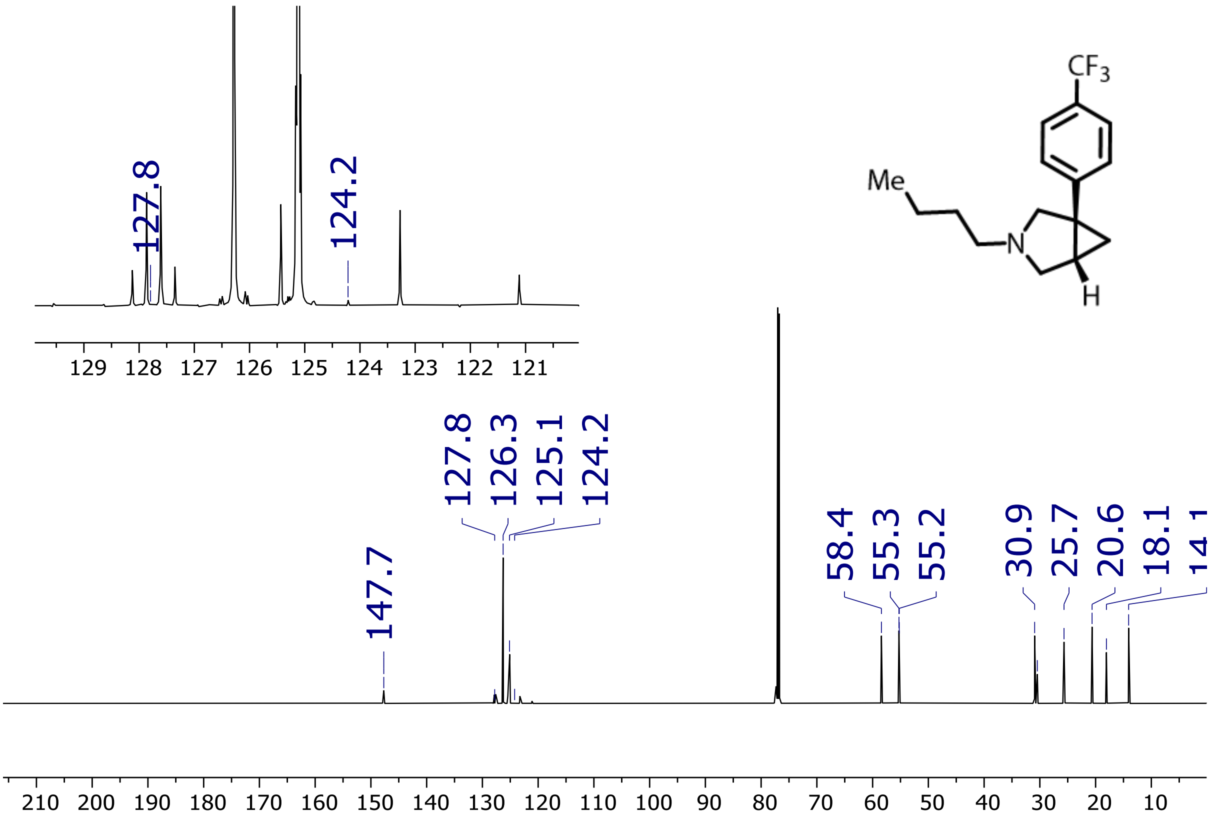

^{19}F NMR (376 MHz, $CDCl_3$):

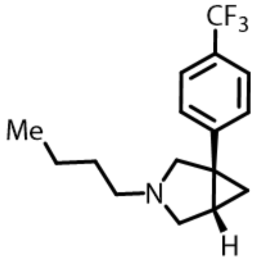

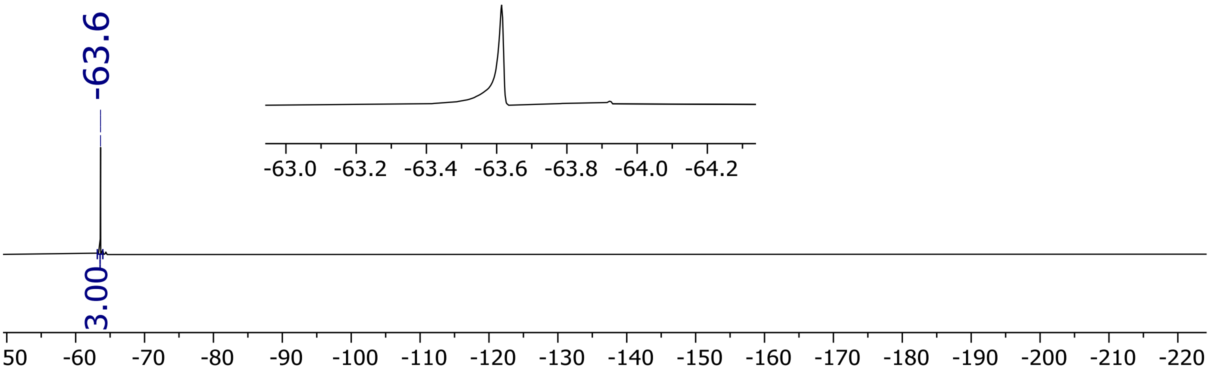

NMR Spectra of Novel Ar-CF_2H Products

4'-(Difluoromethyl)-2,6-dimethyl-1,1'-biphenyl, ***4b***

^{1}H NMR (400 MHz, $CDCl_3$):

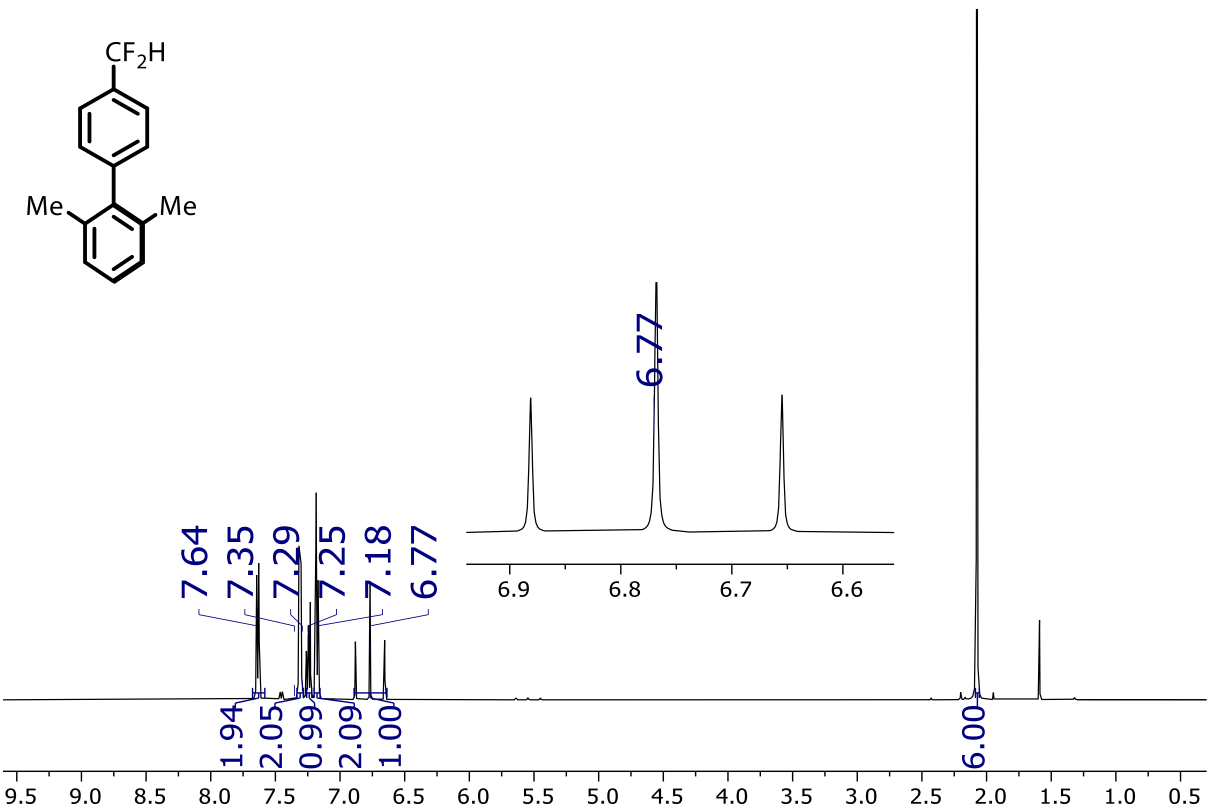

^{13}C NMR (151 MHz, $CDCl_3$):

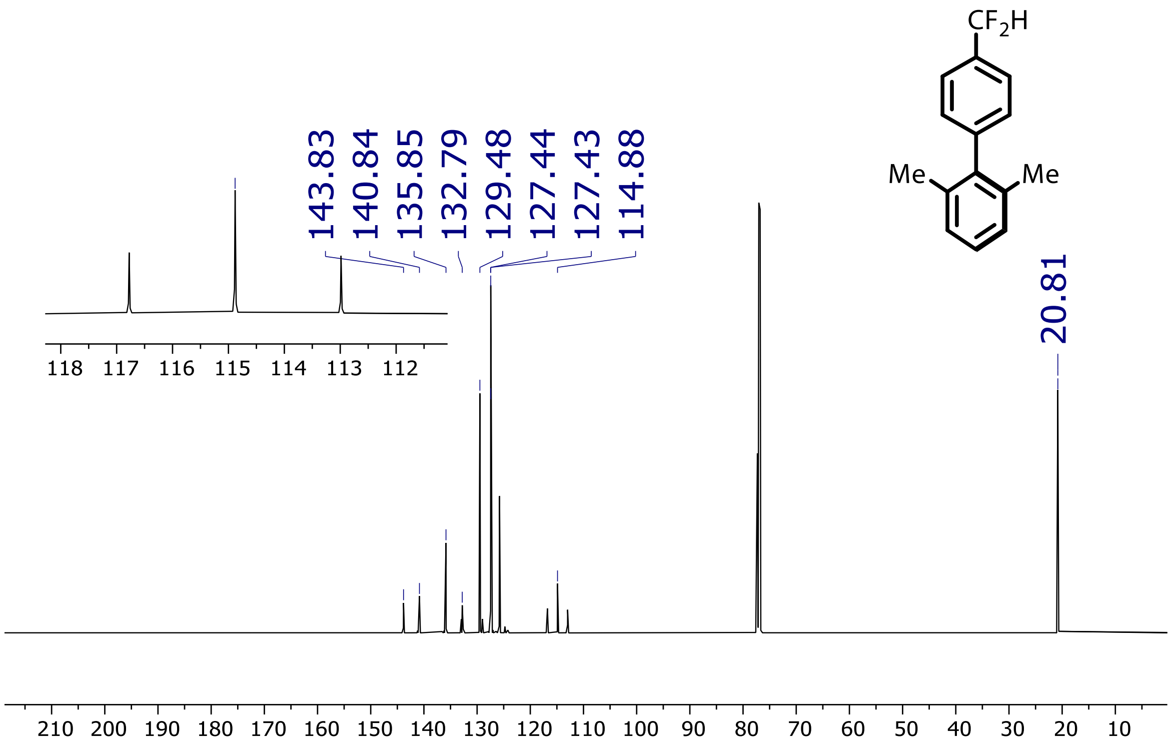

19F NMR (376 MHz, $CDCl_3$):

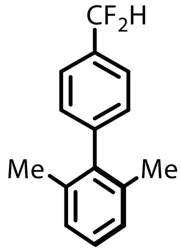

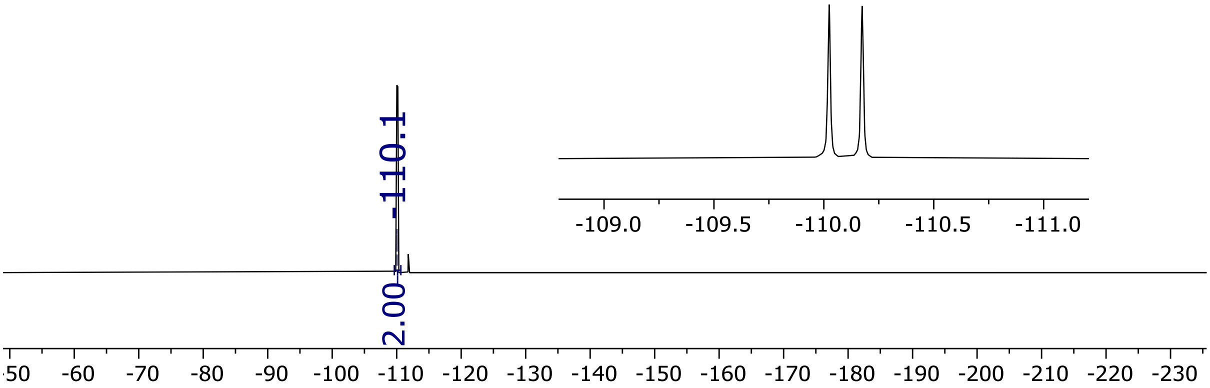

10-(4-(Difluoromethyl)phenyl)-10H-phenothiazine, ***7b***

^{1}H NMR (400 MHz, $CDCl_3$):

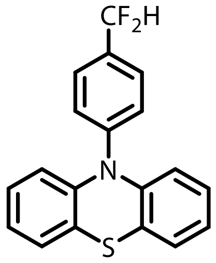

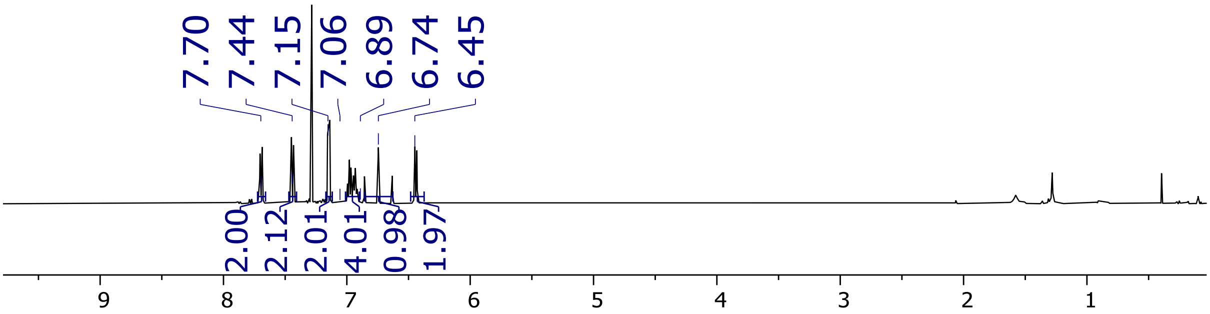

^{13}C NMR (151 MHz, $CDCl_3$):

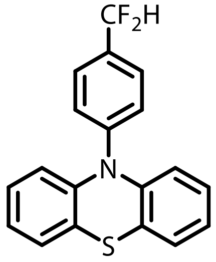

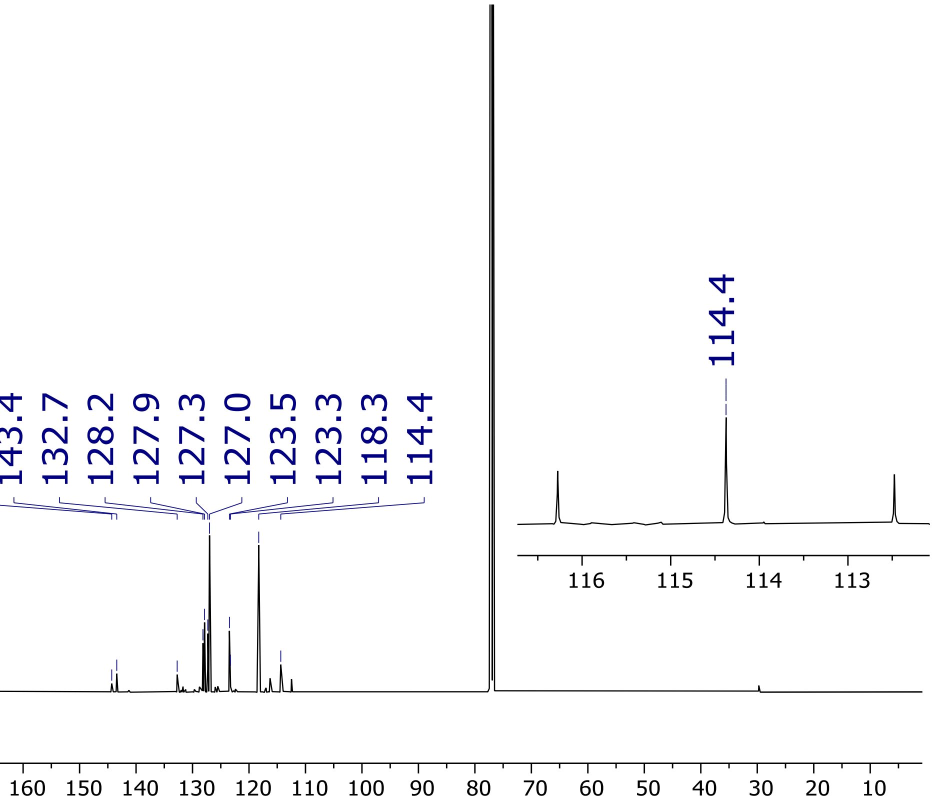

^{19}F NMR (376 MHz, $CDCl_3$):

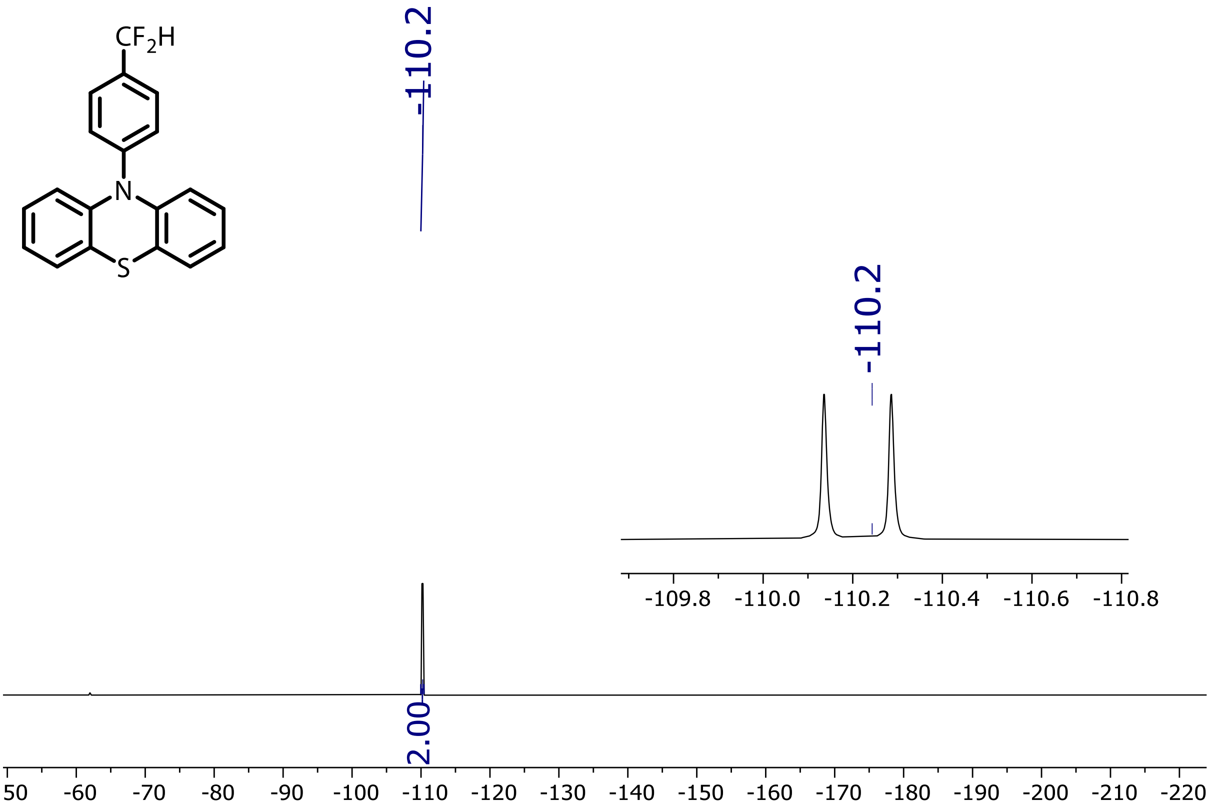

1-Butyl-3-(difluoromethyl)-1H-pyrazole, ***9b***

^{1}H NMR (400 MHz, $CDCl_3$):

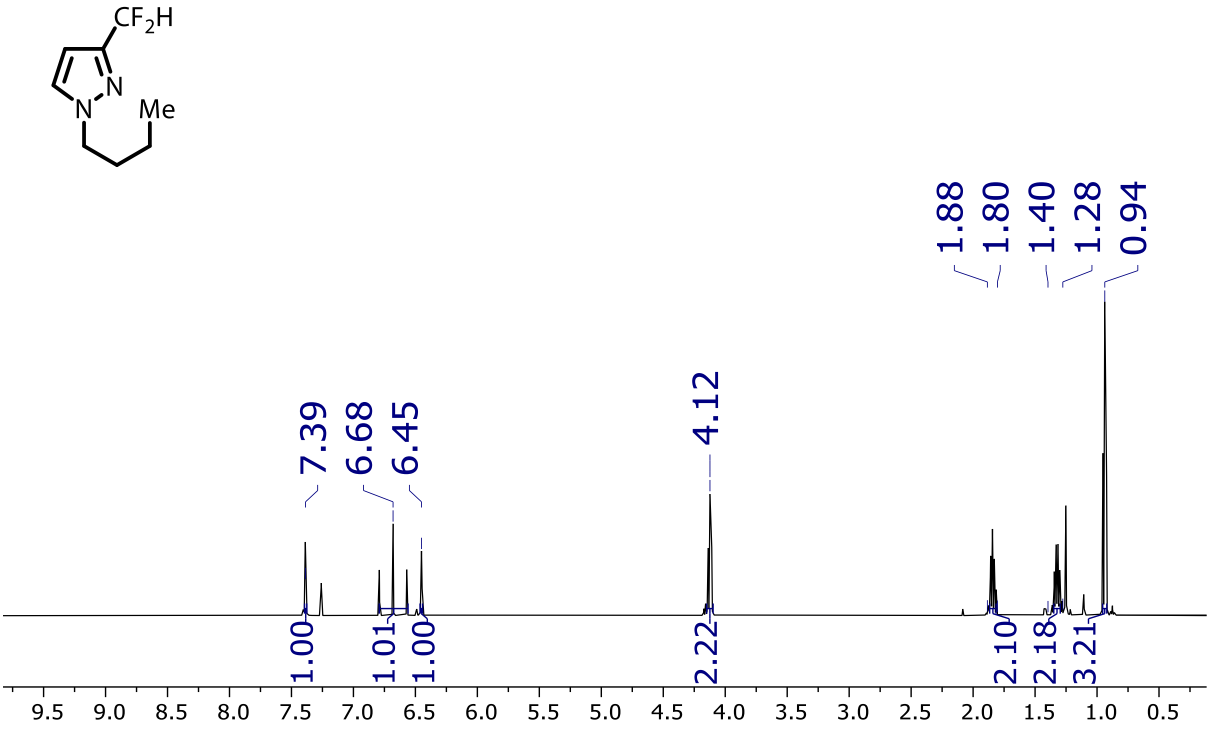

^{13}C NMR (151 MHz, $CDCl_3$):

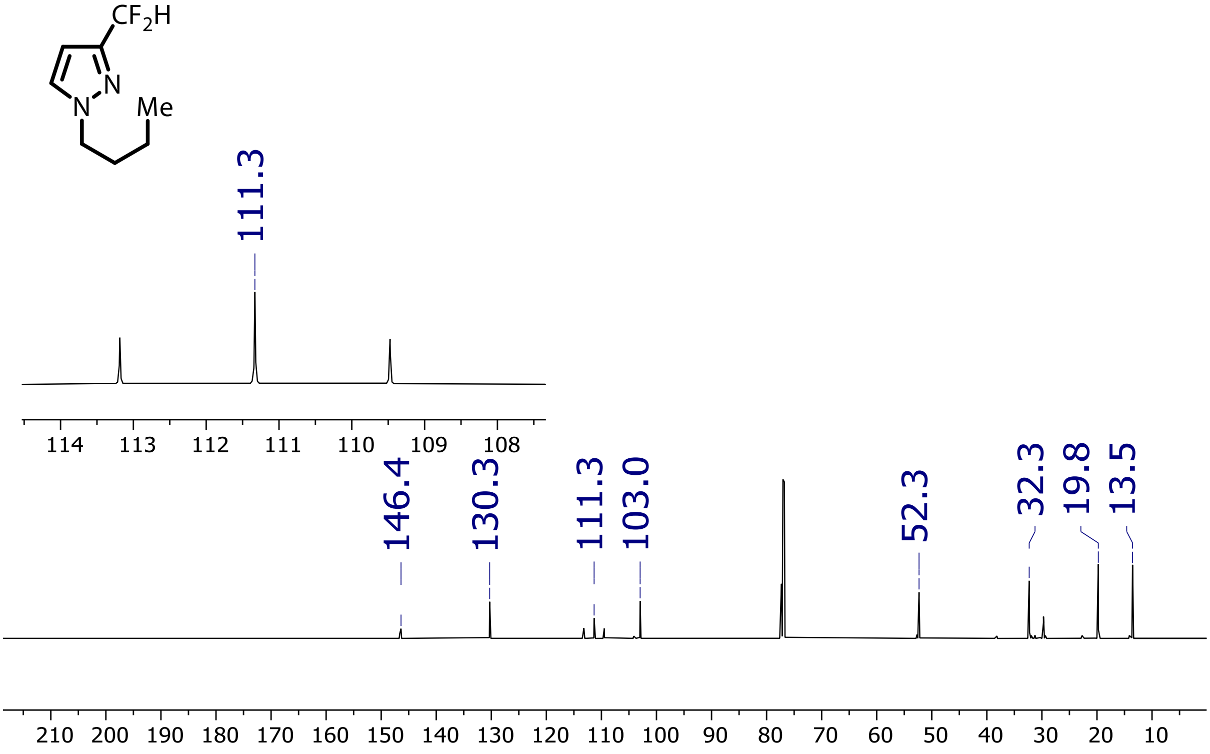

^{19}F NMR (376 MHz, $CDCl_3$):

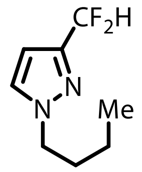

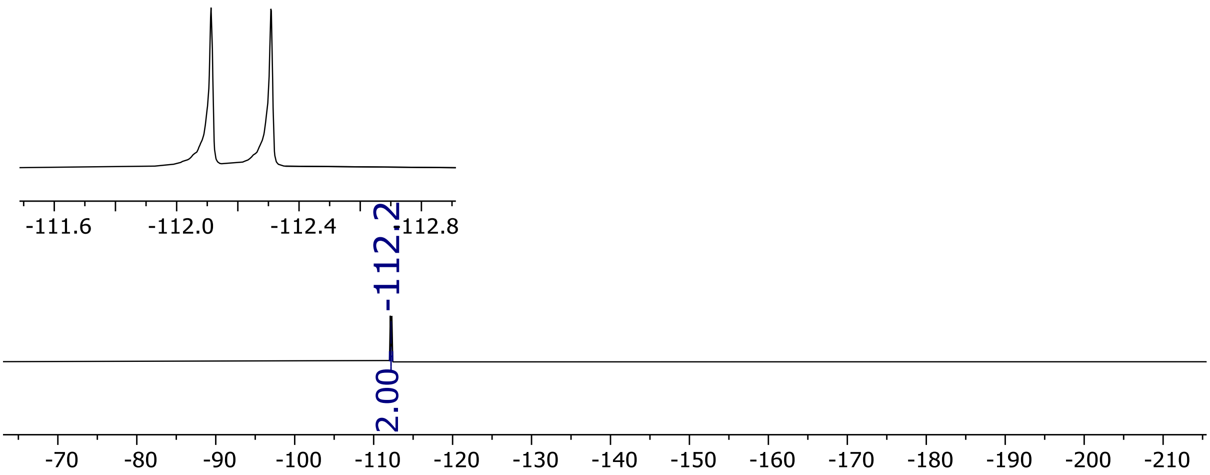

8-(4-(Difluoromethyl)phenyl)-1,4-dioxa-8-azaspiro[4.5]decane, ***11b***

^{1}H NMR (400 MHz, $CDCl_3$):

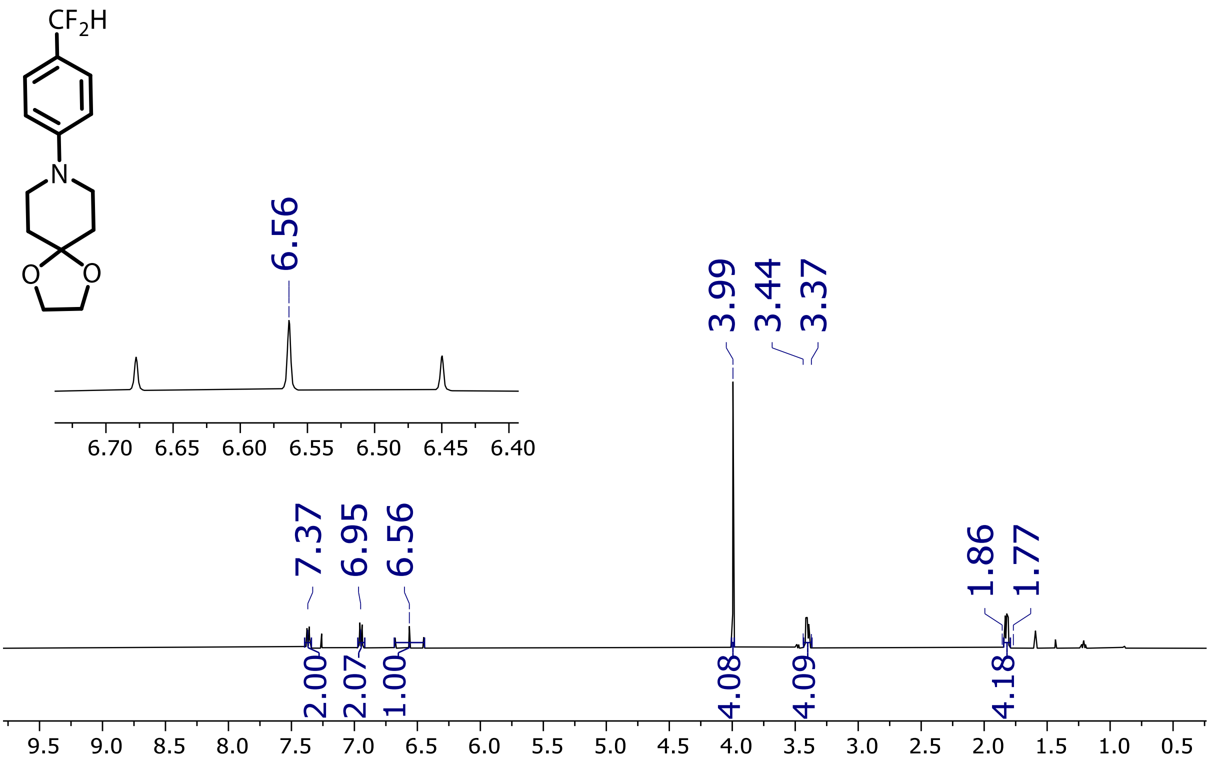

^{13}C NMR (151 MHz, $CDCl_3$):

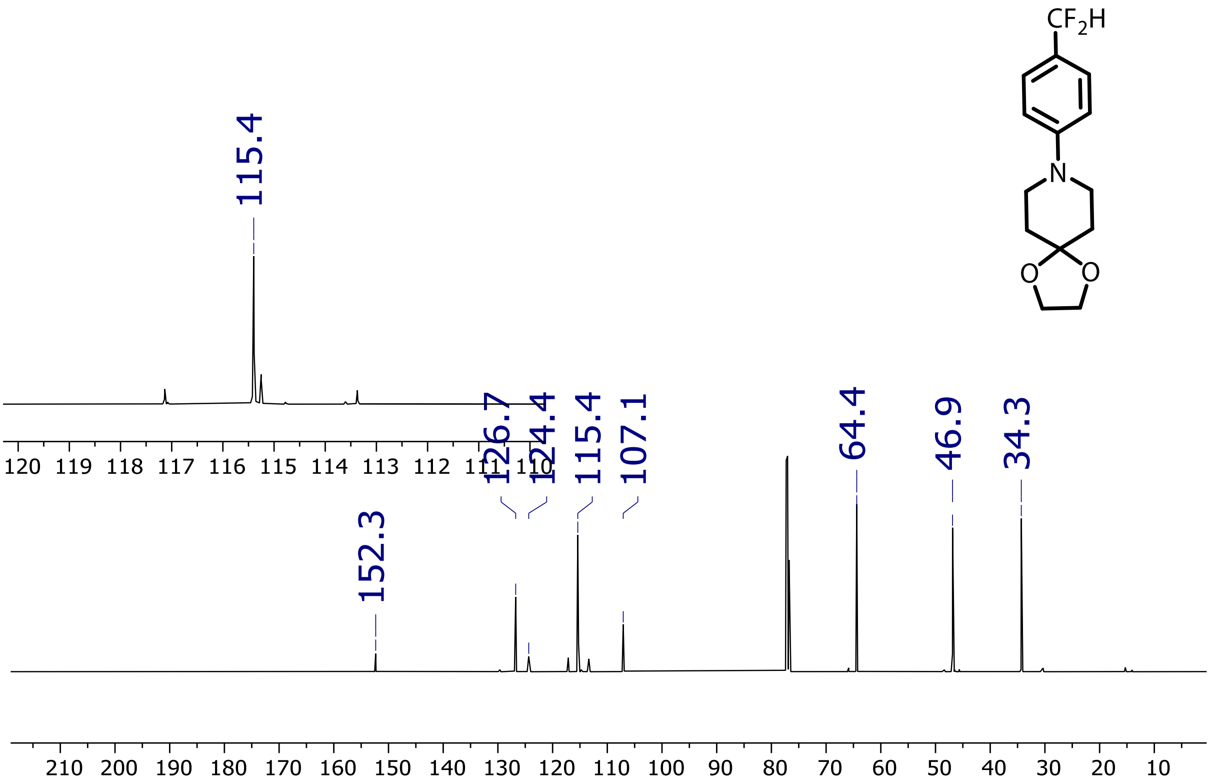

^{19}F NMR (376 MHz, $CDCl_3$):

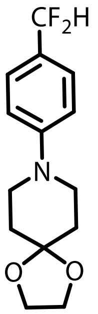

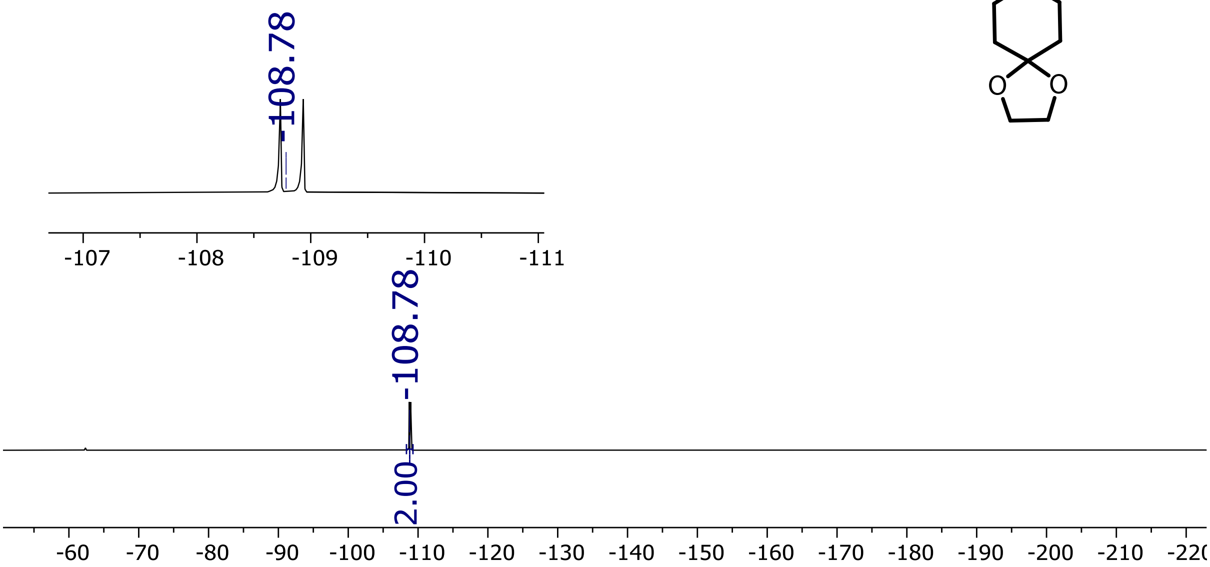

Tert-butyl 4-(4-(difluoromethyl)phenyl)piperazine-1-carboxylate, ***12b***

^{1}H NMR (400 MHz, $CDCl_3$):

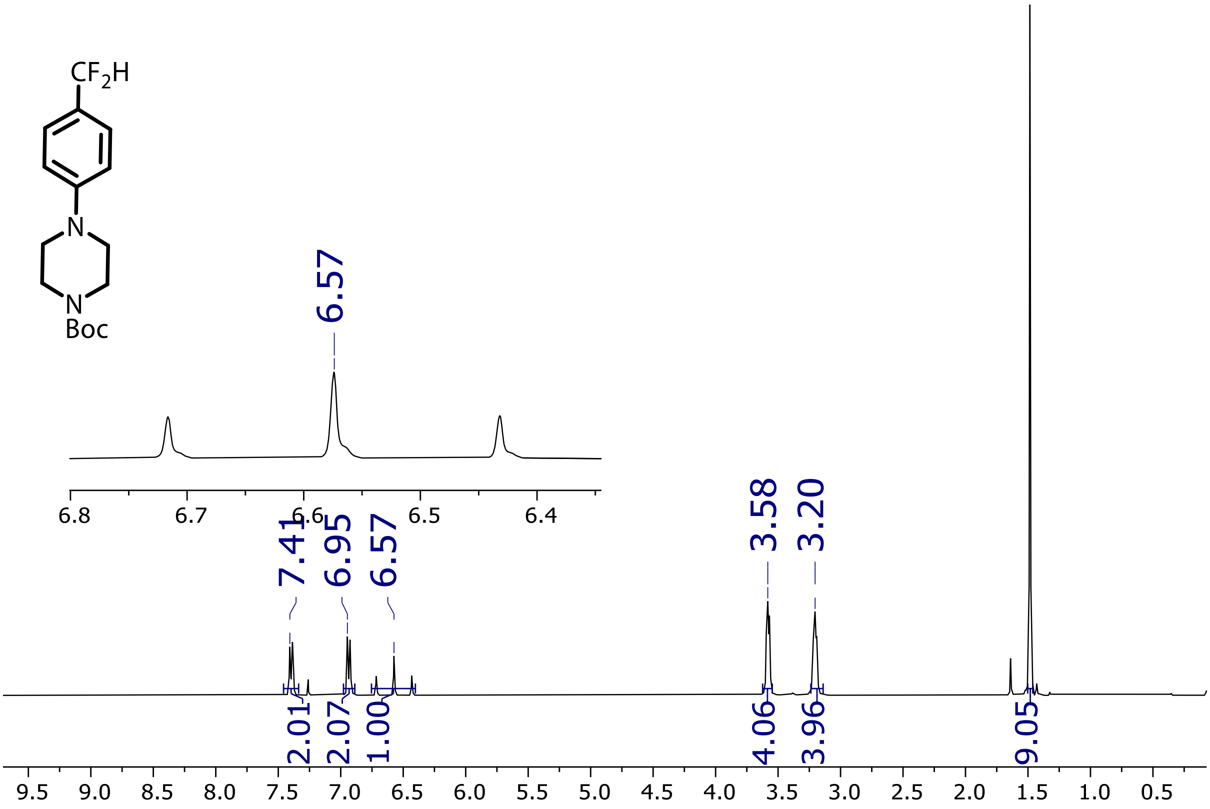

^{13}C NMR (151 MHz, $CDCl_3$):

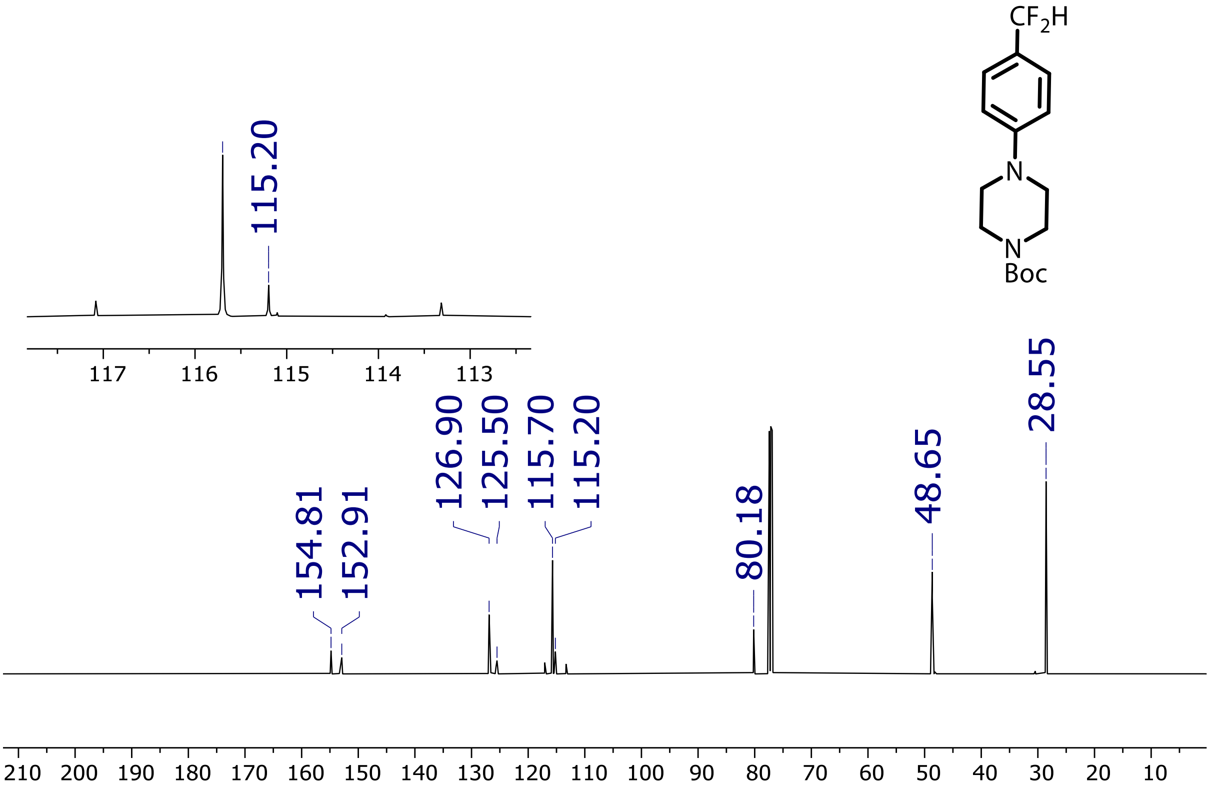

^{19}F NMR (376 MHz, $CDCl_3$):

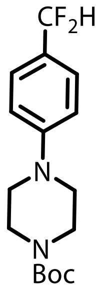

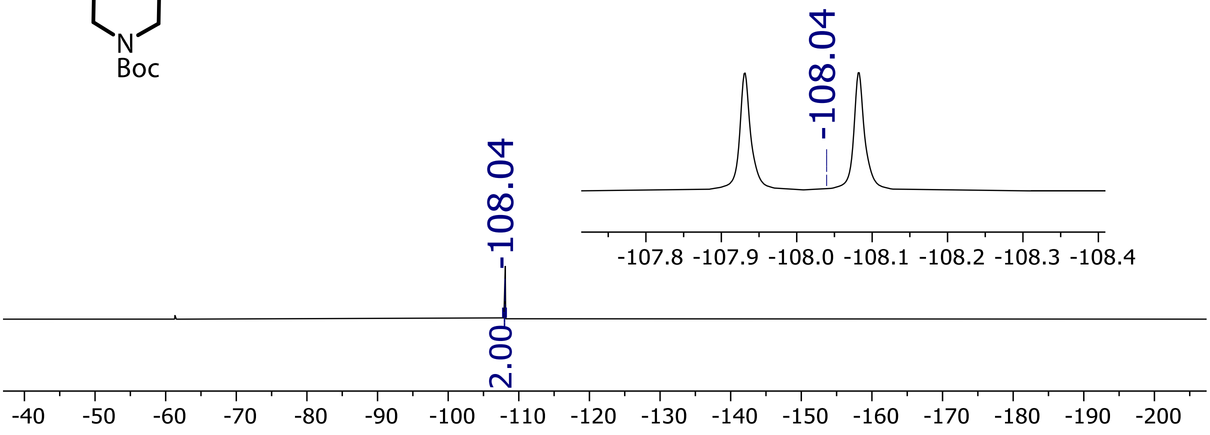

Diethyl (4-(difluoromethyl)benzyl)phosphonate, ***15b***

^{1}H NMR (400 MHz, $CDCl_3$):

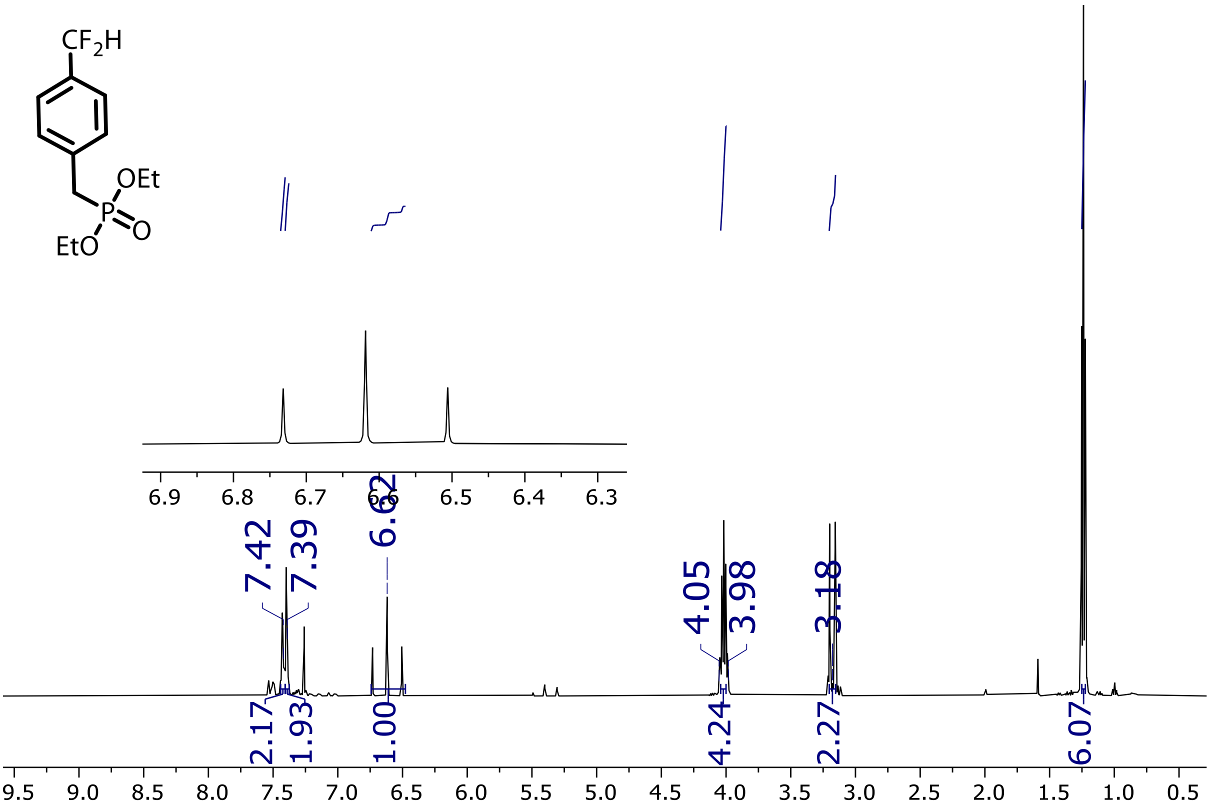

^{13}C NMR (151 MHz, $CDCl_3$):

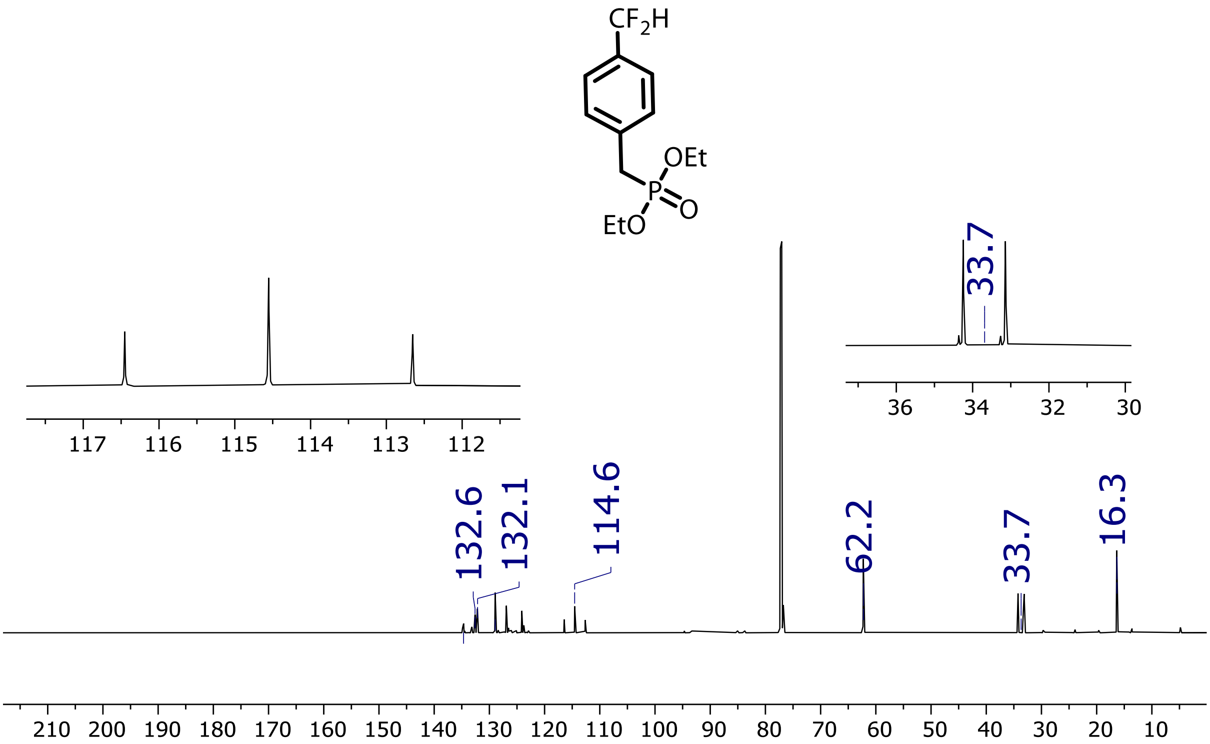

^{19}F NMR (376 MHz, $CDCl_3$):

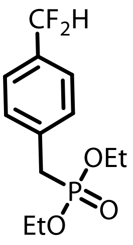

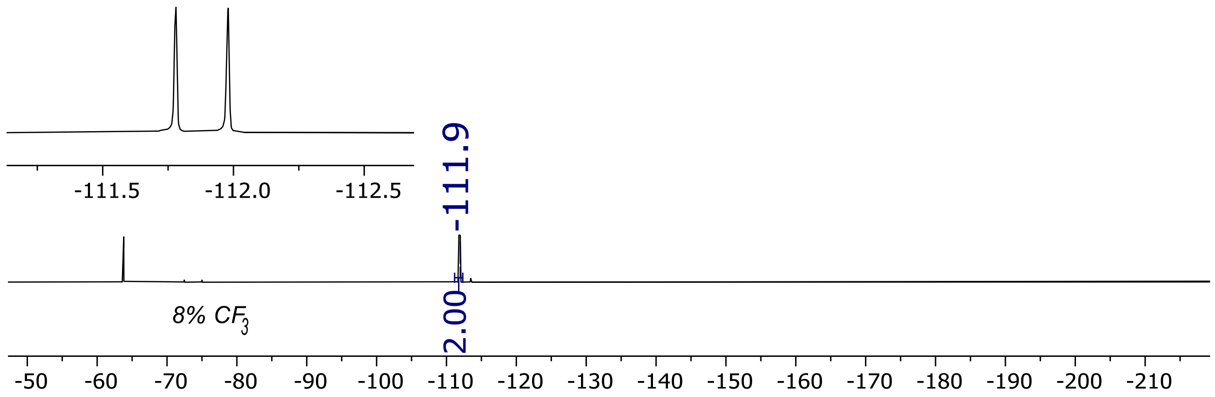

*1-Allyl-4-(difluoromethyl)benzene, **16b***
^{1}H NMR (400 MHz, $CDCl_3$):

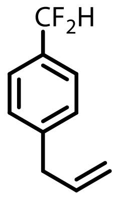

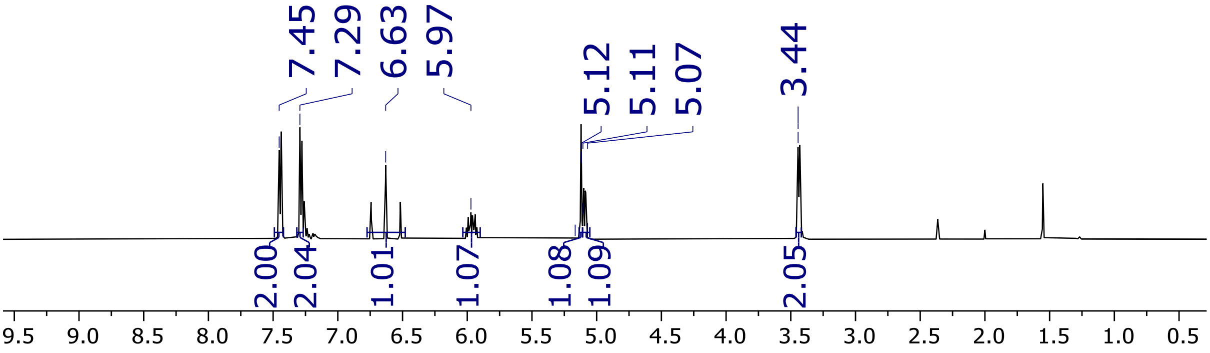

^{13}C NMR (151 MHz, $CDCl_3$):

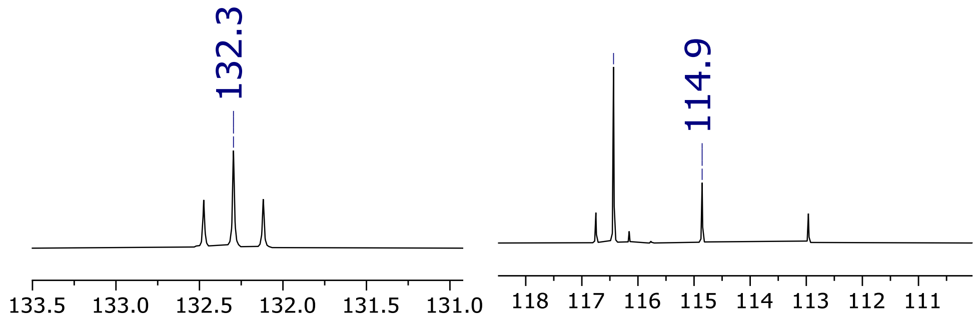

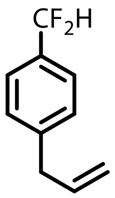

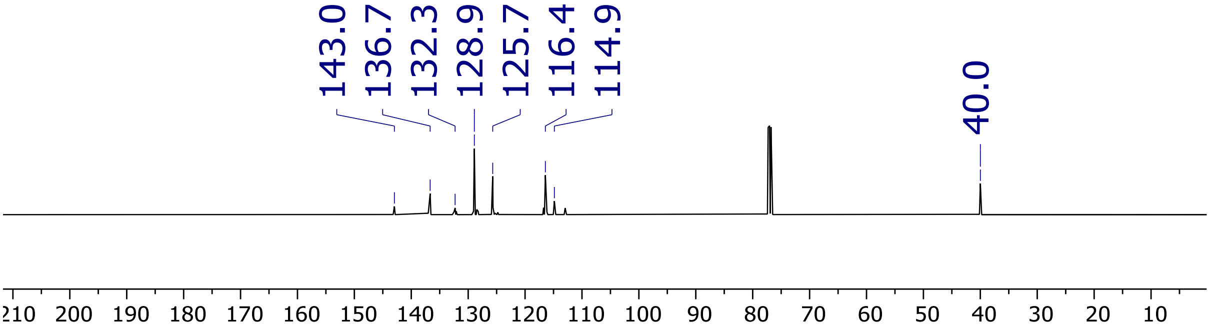

^{19}F NMR (376 MHz, $CDCl_3$):

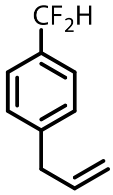

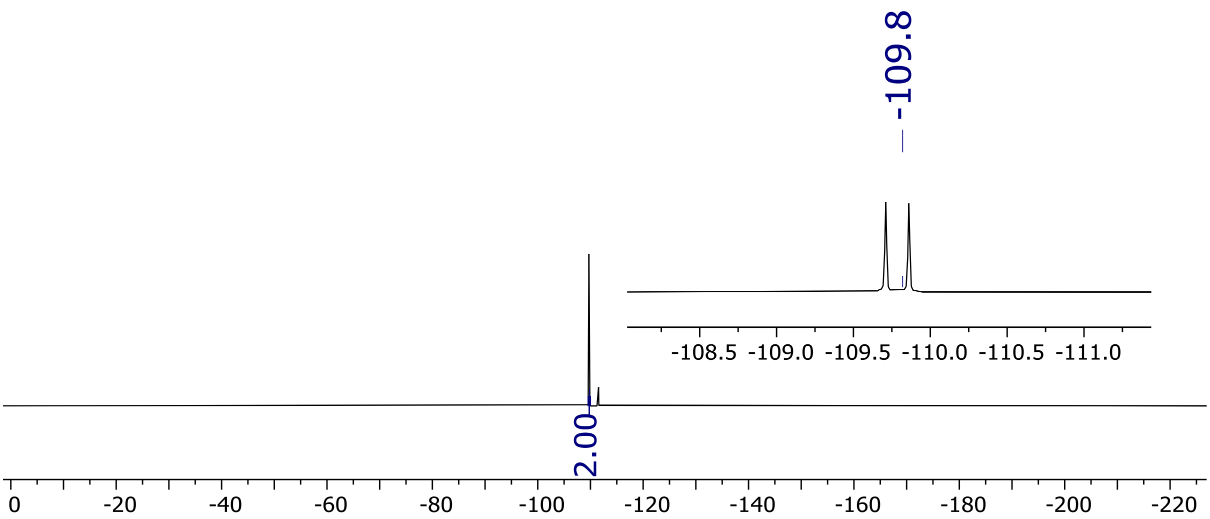

*2-Allyl-1-(difluoromethyl)-4-fluorobenzene, **17b***

^{1}H NMR (400 MHz, $CDCl_3$):

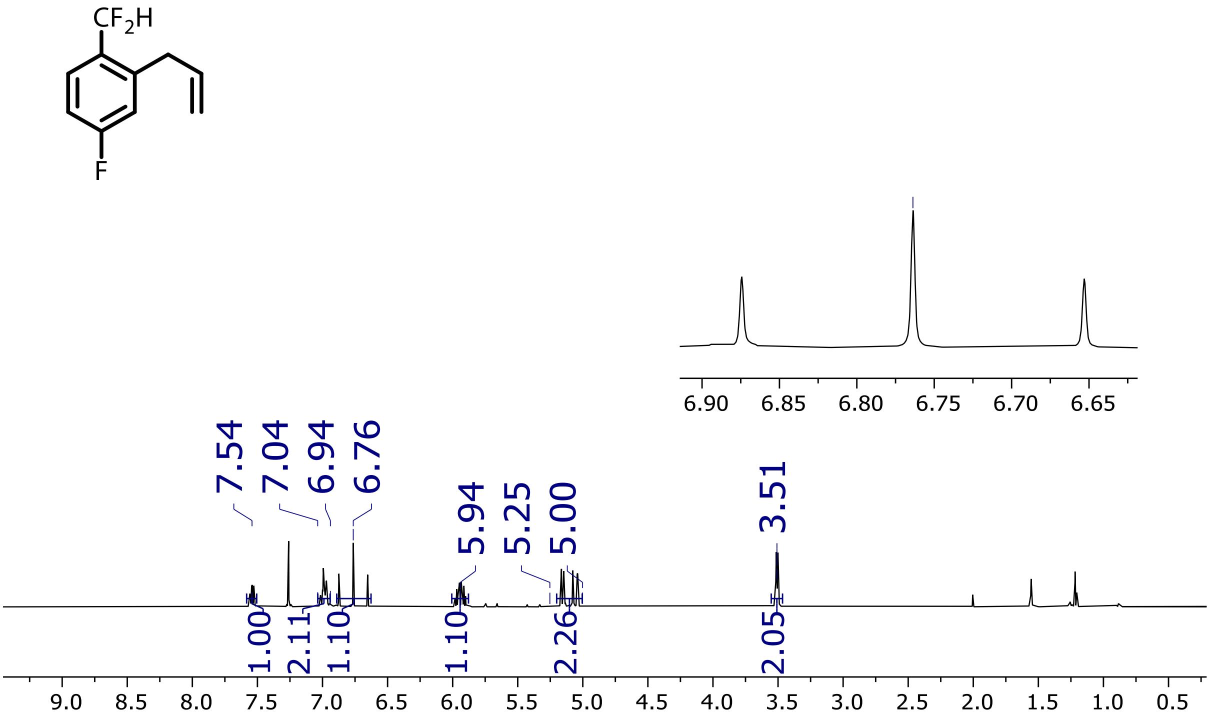

^{13}C NMR (151 MHz, $CDCl_3$):

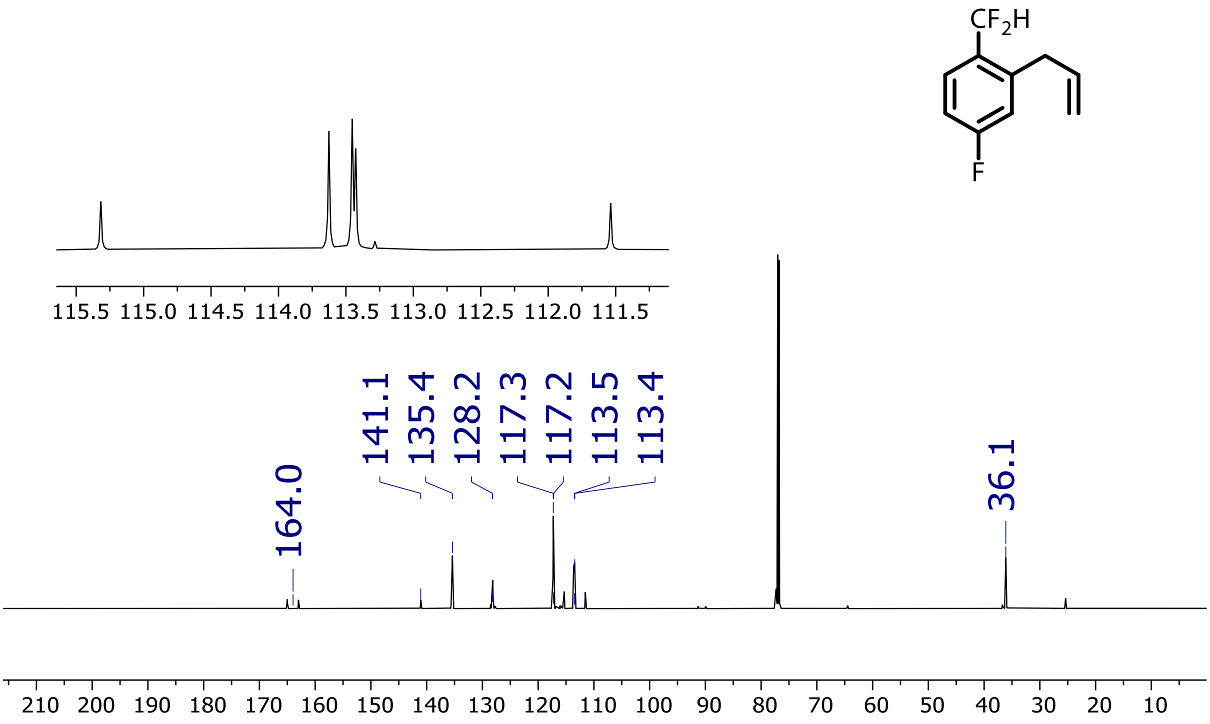

^{19}F NMR (376 MHz, $CDCl_3$):

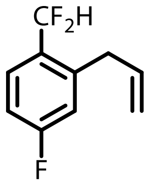

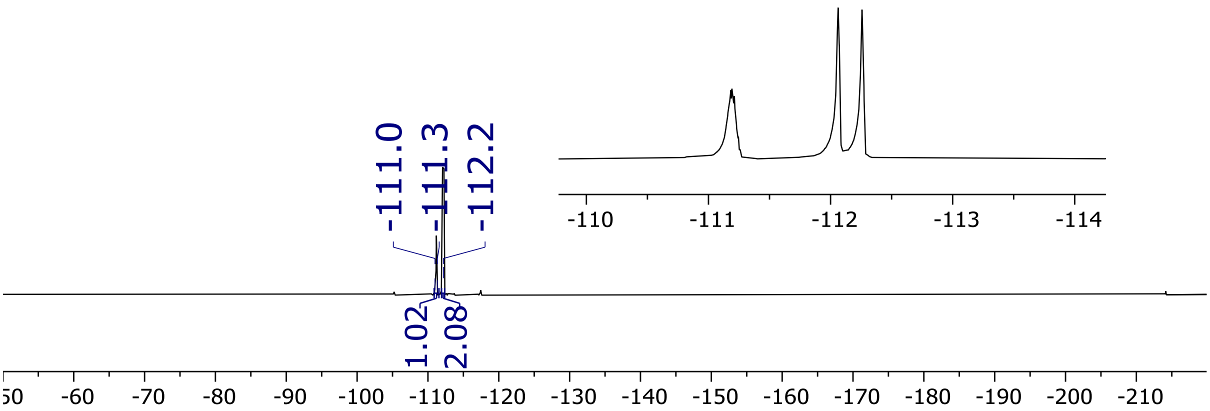

N-(4-(Difluoromethyl)benzyl)prop-2-en-1-amine, ***18b***

^{1}H NMR (400 MHz, $CDCl_3$):

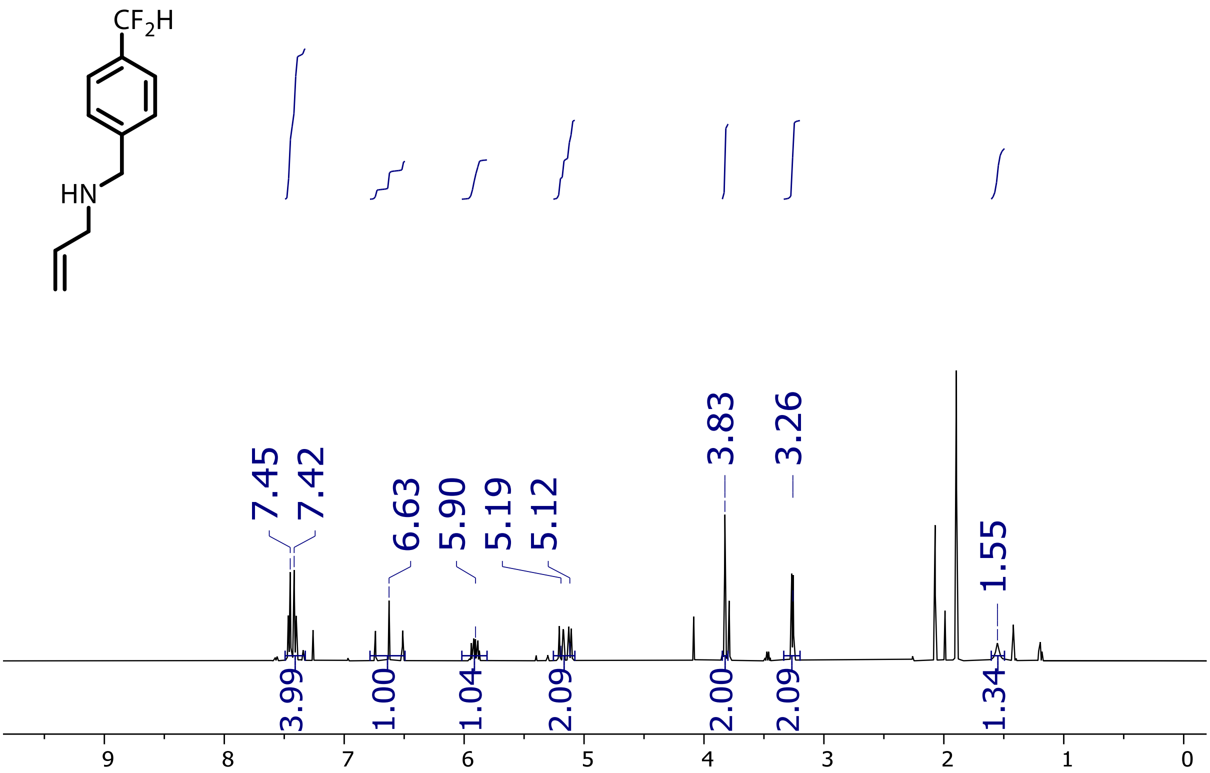

^{13}C NMR (151 MHz, $CDCl_3$):

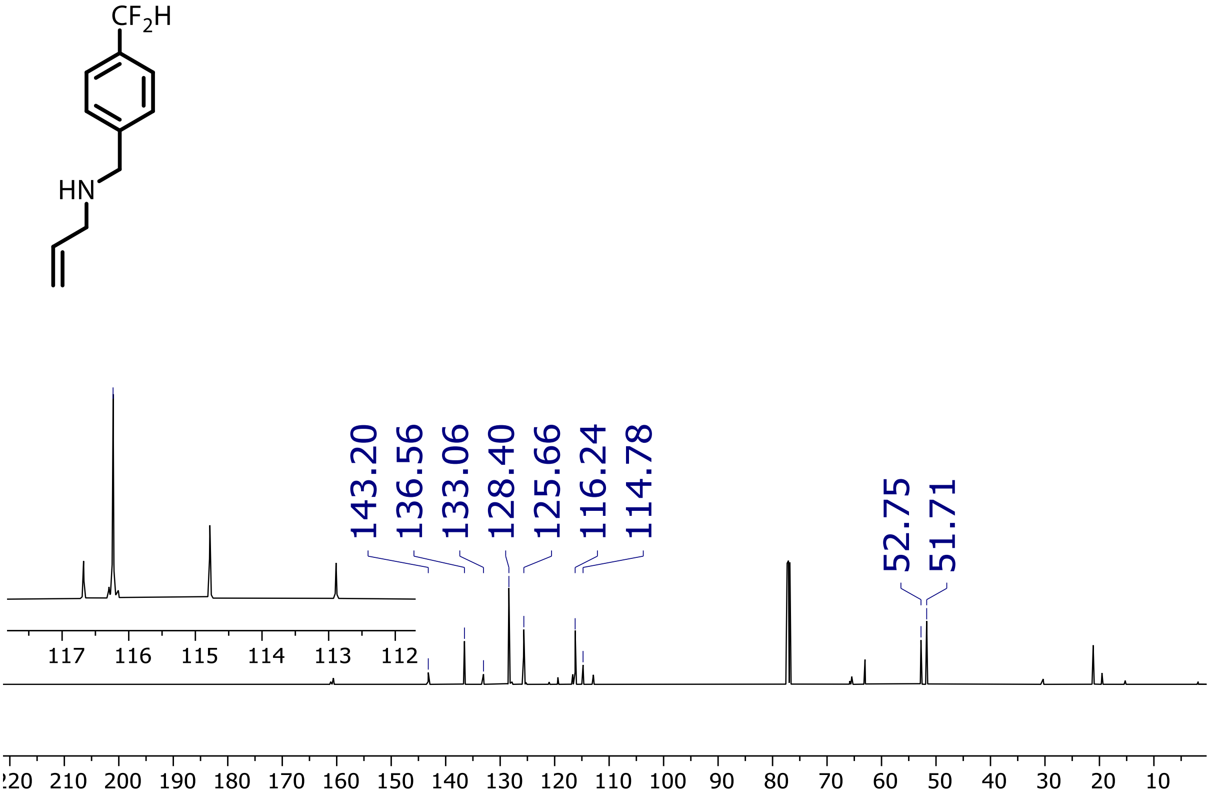

^{19}F NMR (376 MHz, $CDCl_3$):

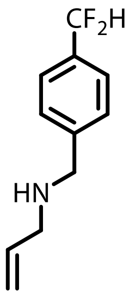

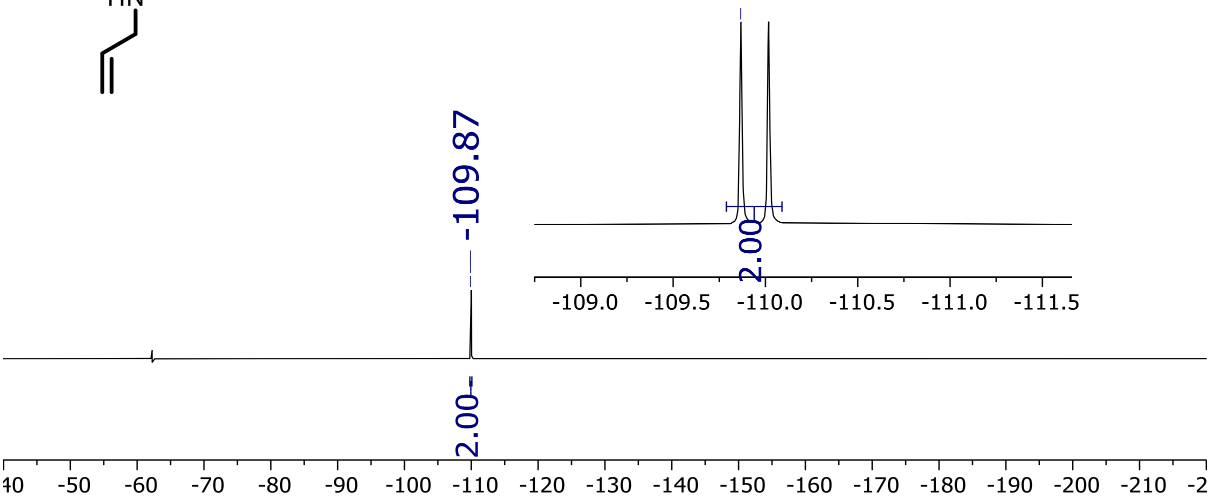

*Diethyl 4-(difluoromethyl)isophthalate, **25b***

^{1}H NMR (400 MHz, $CDCl_3$):

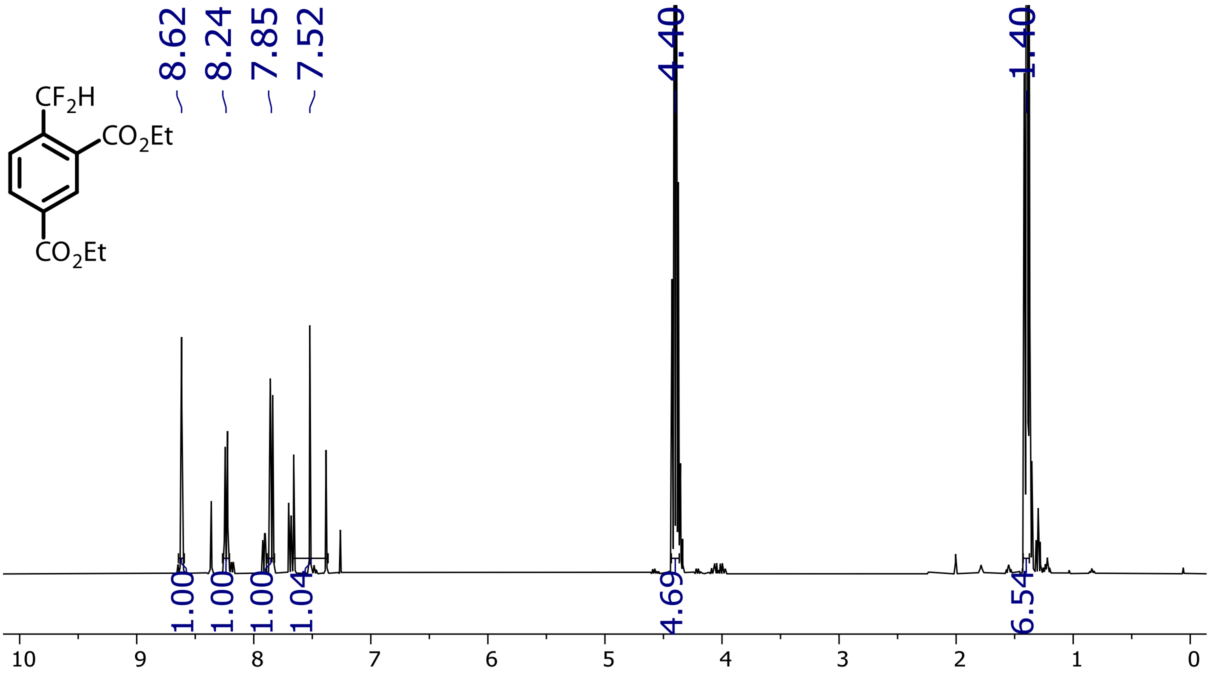

^{13}C NMR (151 MHz, $CDCl_3$):

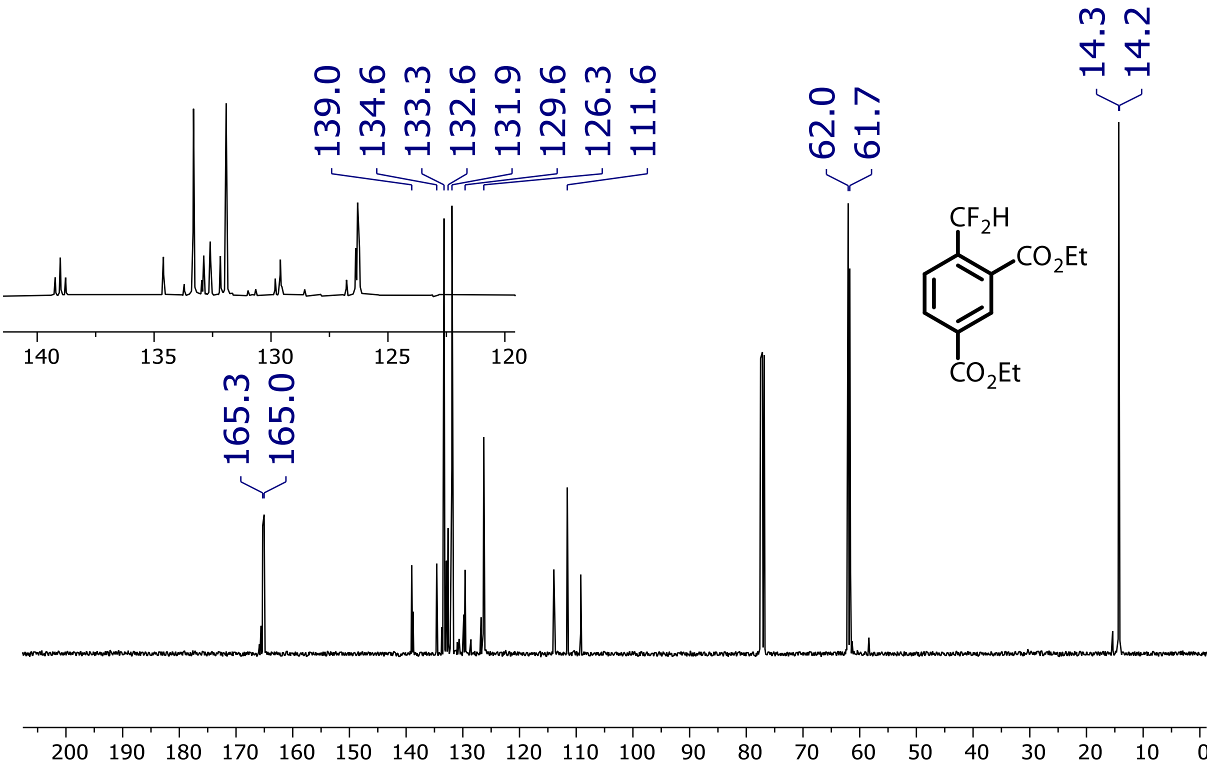

^{19}F NMR (376 MHz, $CDCl_3$):

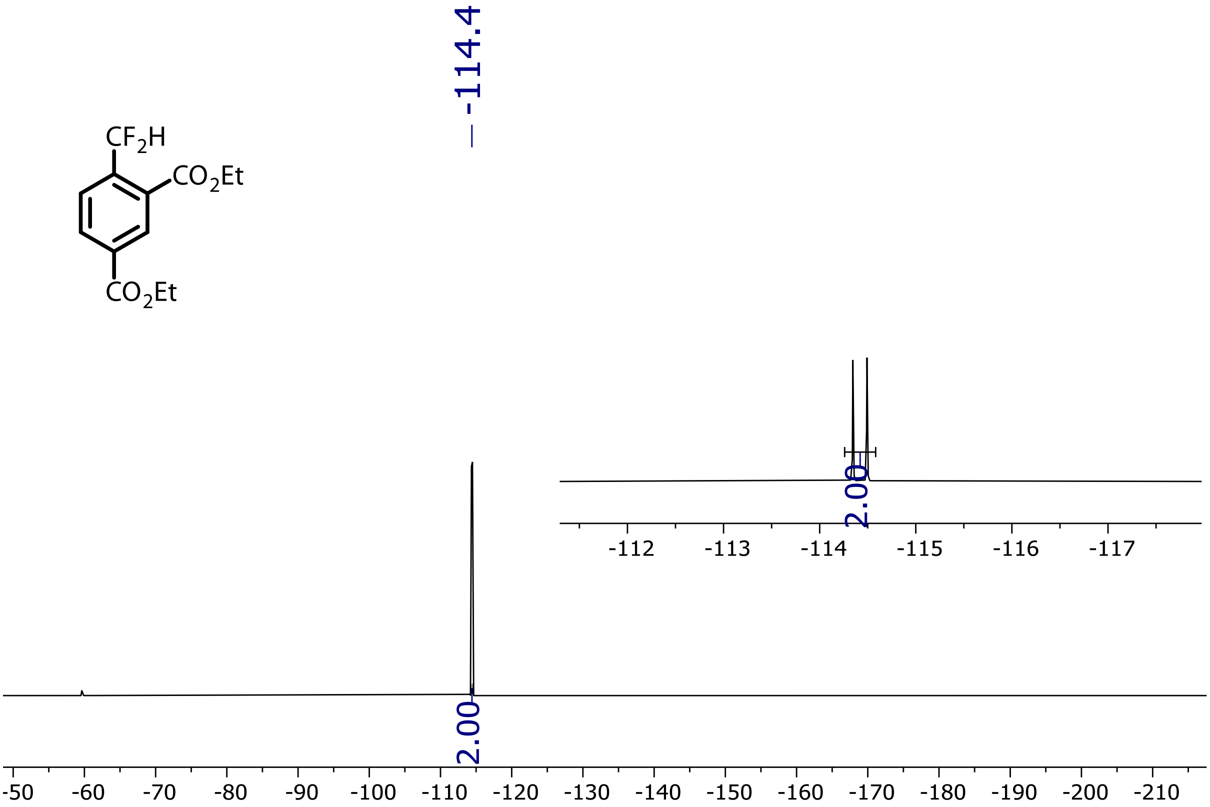

2-(Difluoromethyl)-5-methoxy-1,3-dimethyl-1H-indole, ***26b***

^{1}H NMR (400 MHz, $CDCl_3$):

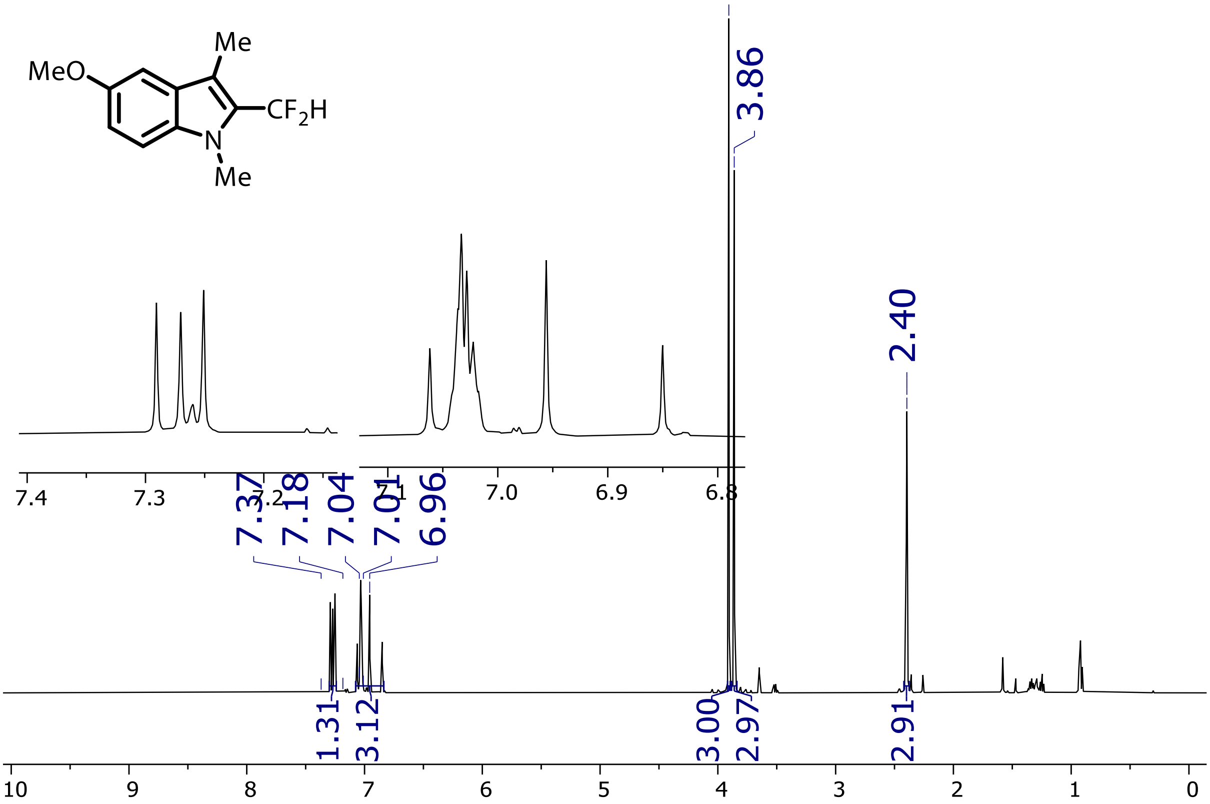

^{13}C NMR (151 MHz, $CDCl_3$):

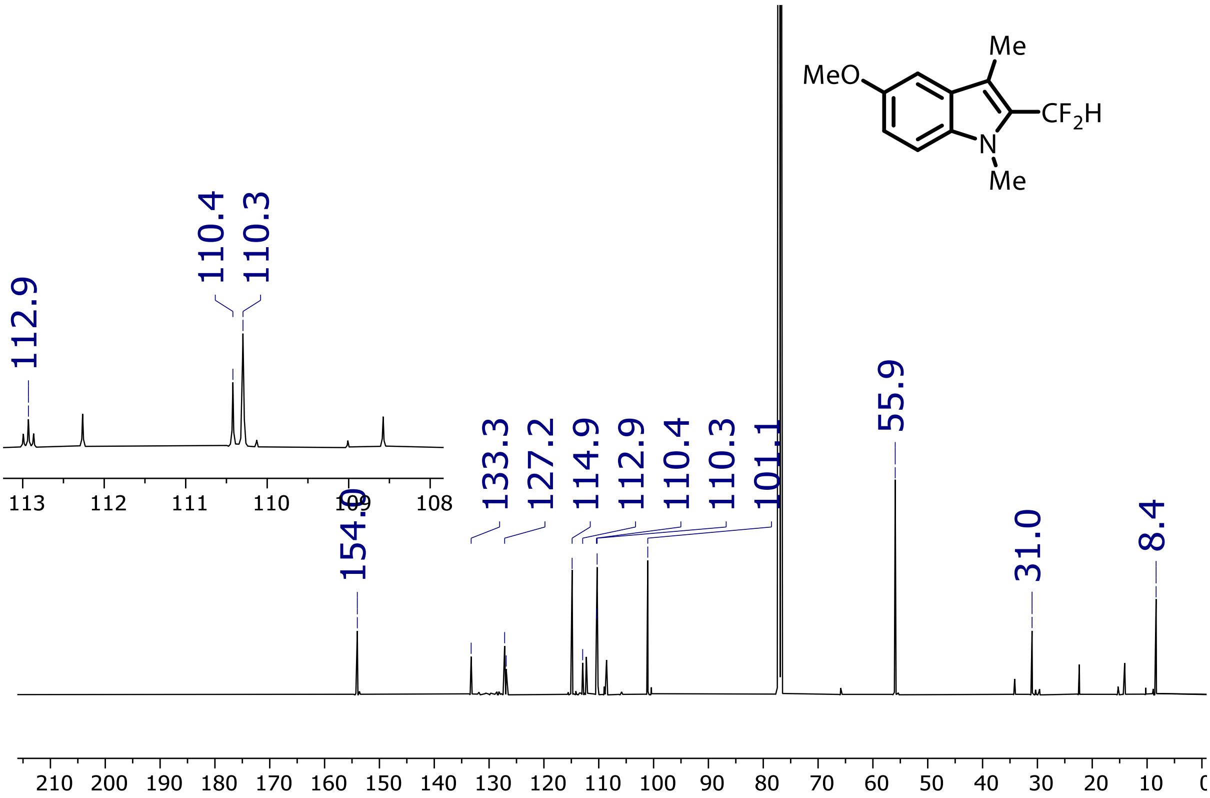

^{19}F NMR (376 MHz, $CDCl_3$):

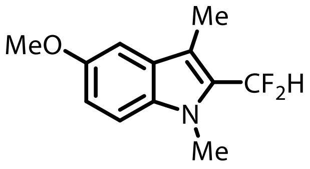

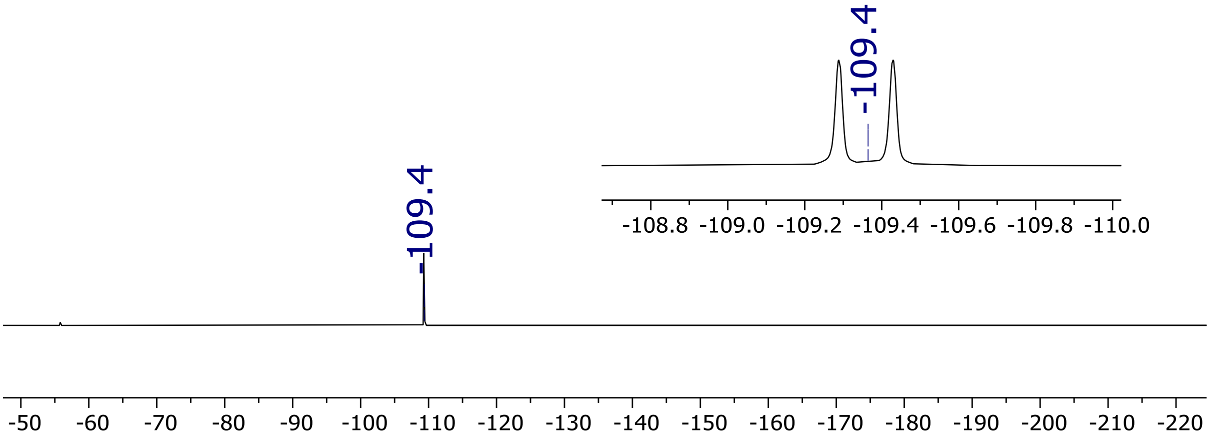

3-(4-(Difluoromethyl)phenoxy)-N-methyl-3-phenylpropan-1-amine, ***27b***

^{1}H NMR (400 MHz, $CDCl_3$):

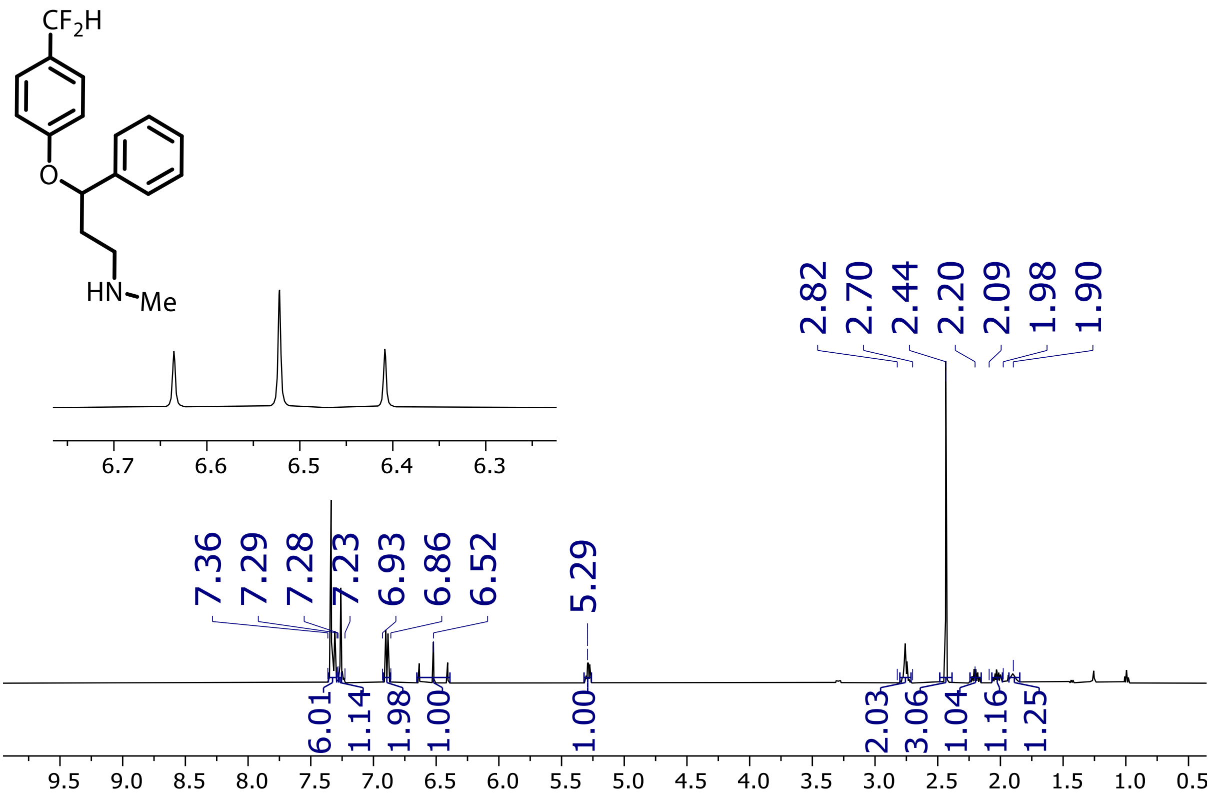

^{13}C NMR (151 MHz, $CDCl_3$):

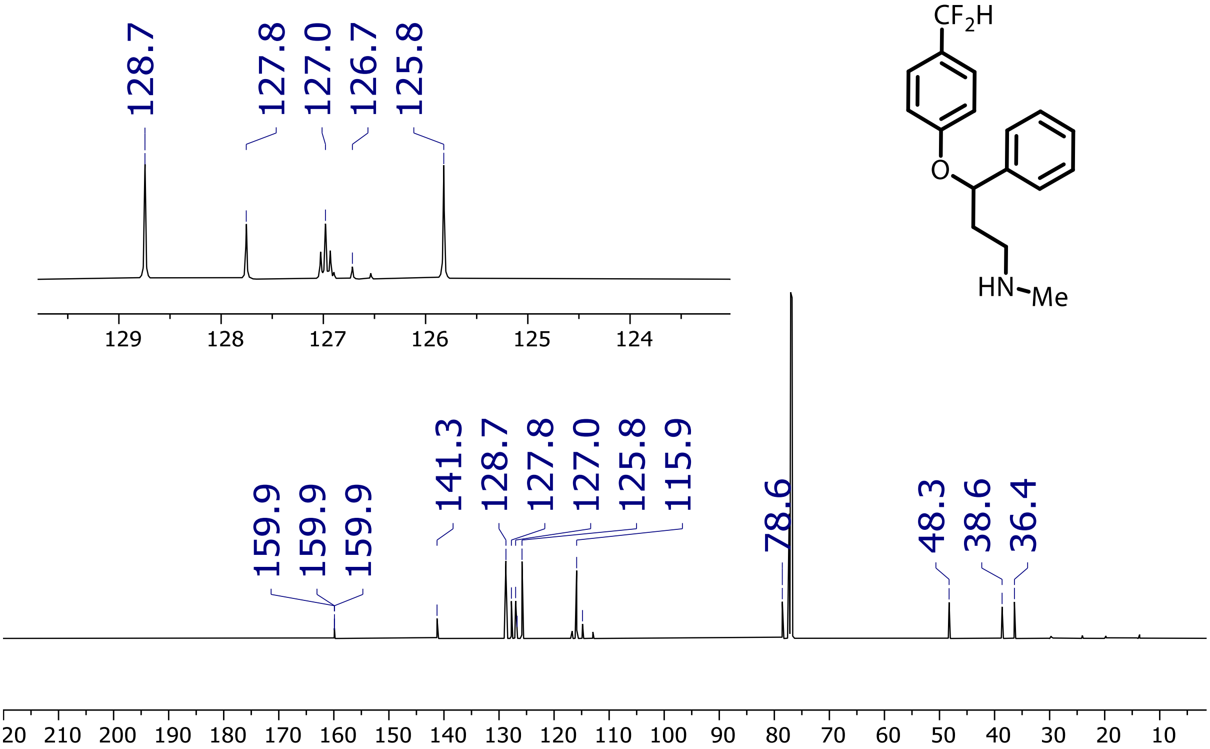

^{19}F NMR (376 MHz, $CDCl_3$):

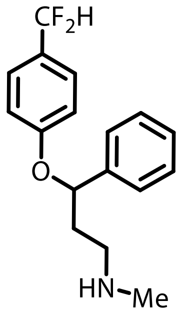

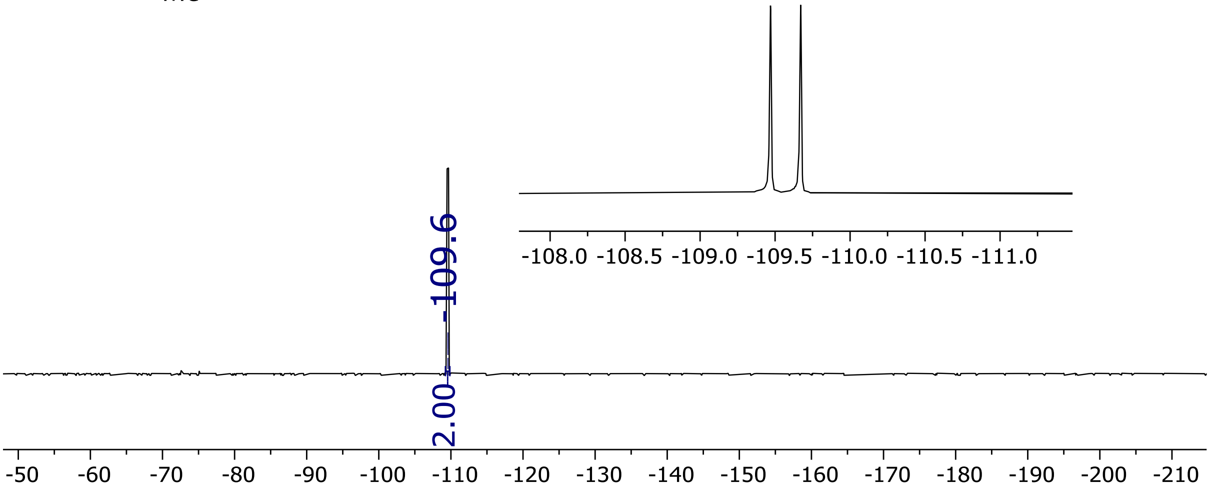

Ethyl-(R)-2-((tert-butoxycarbonyl)amino)-3-(4-(difluoromethyl)phenyl)propanoate, ***29b***

^{1}H NMR (400 MHz, $CDCl_3$):

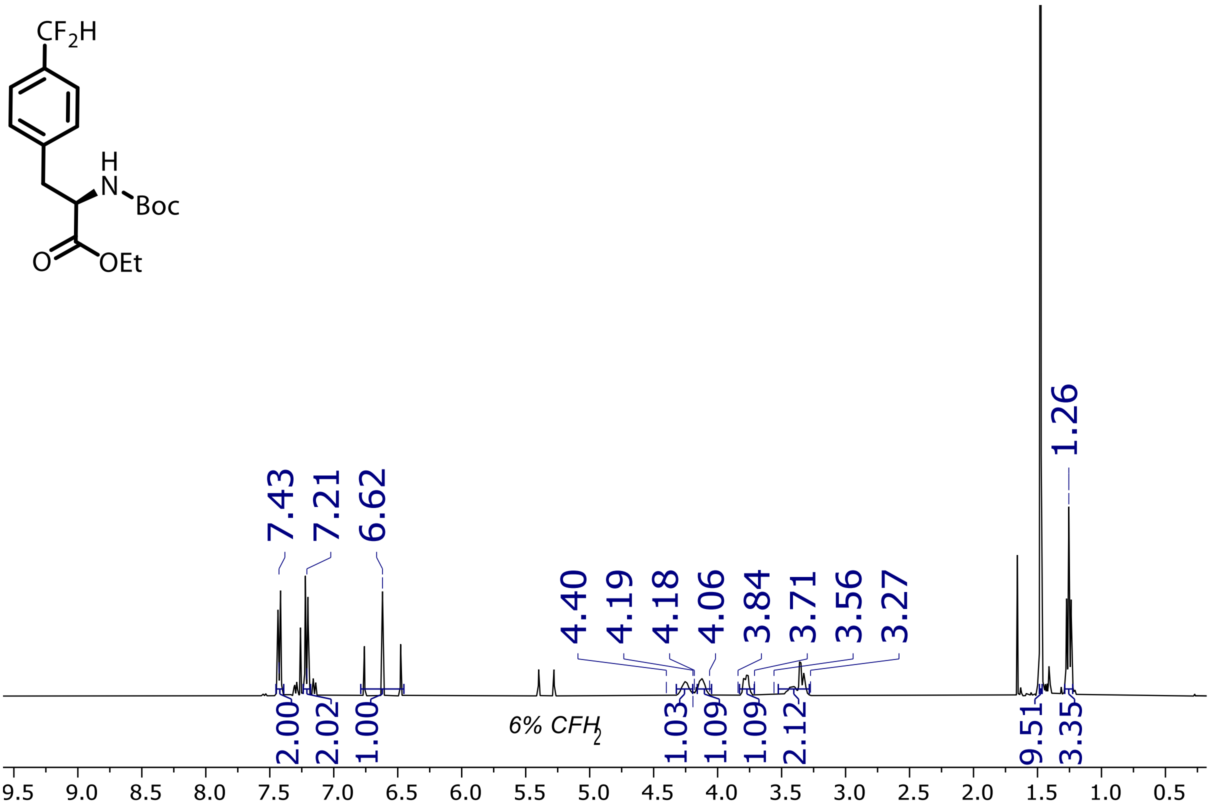

^{13}C NMR (151 MHz, $CDCl_3$):

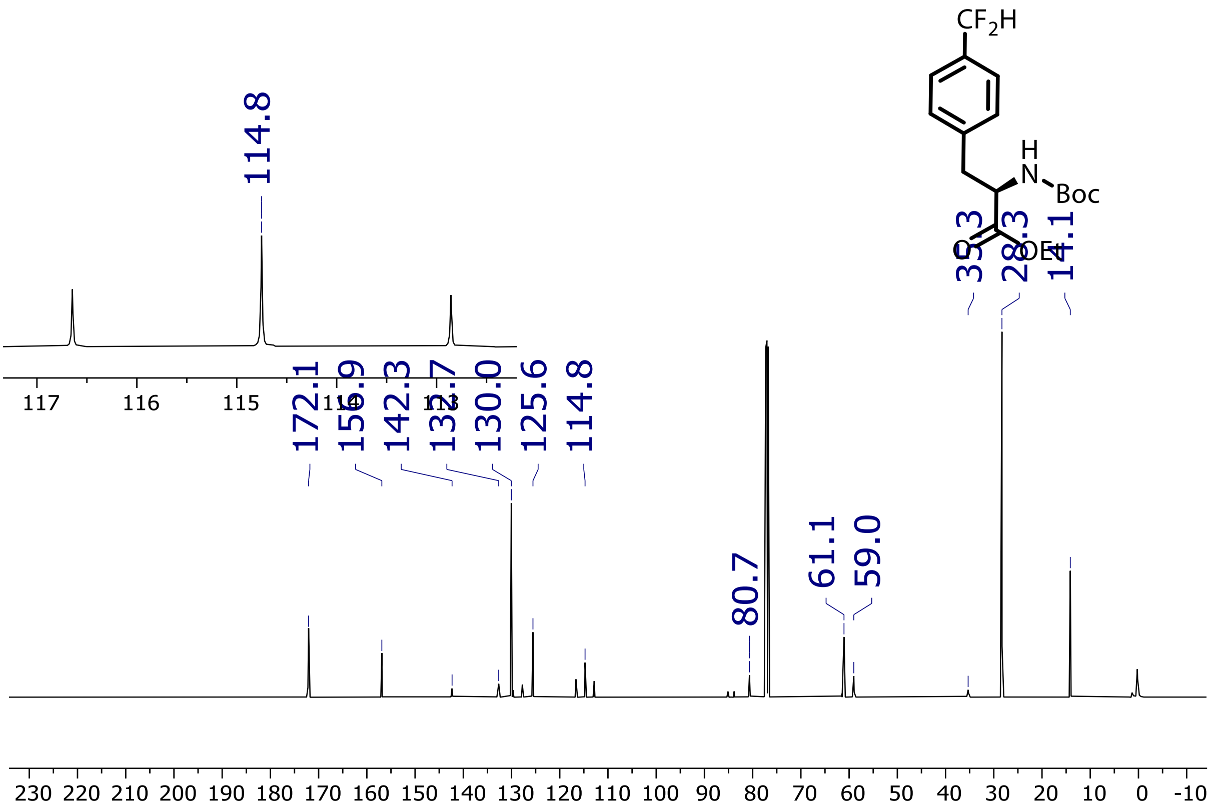

^{19}F NMR (376 MHz, $CDCl_3$):

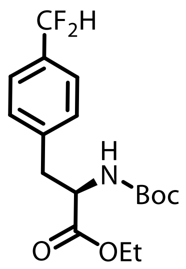

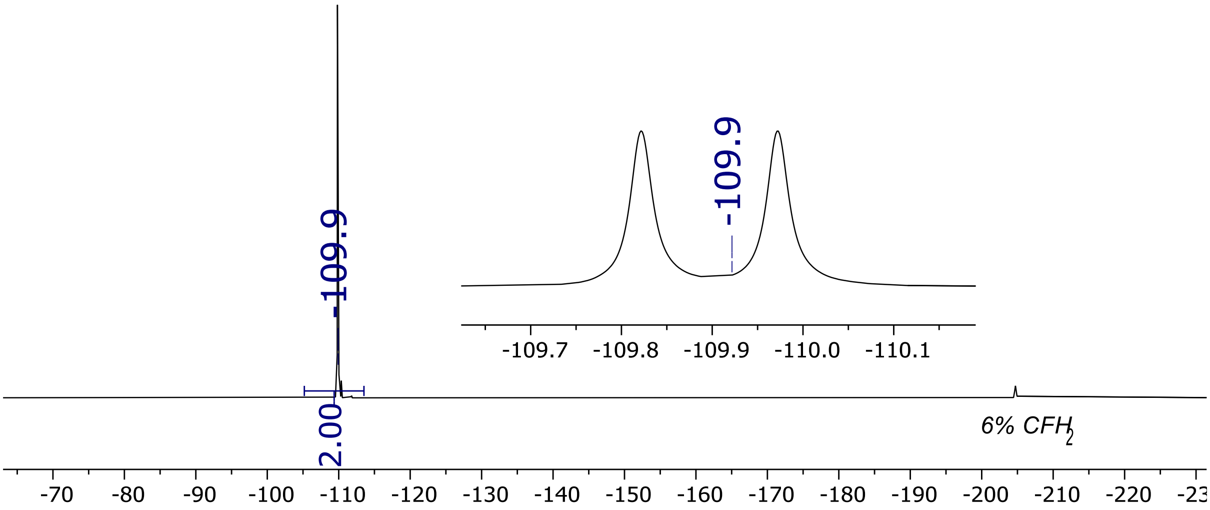

(S)-3-(3-(Difluoromethyl)phenyl)-N-(1-(naphthalen-1-yl)ethyl)propan-1-amine, ***30b***

^{1}H NMR (400 MHz, $CDCl_3$):

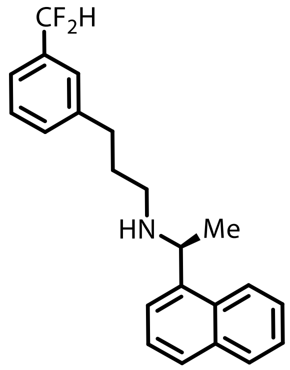

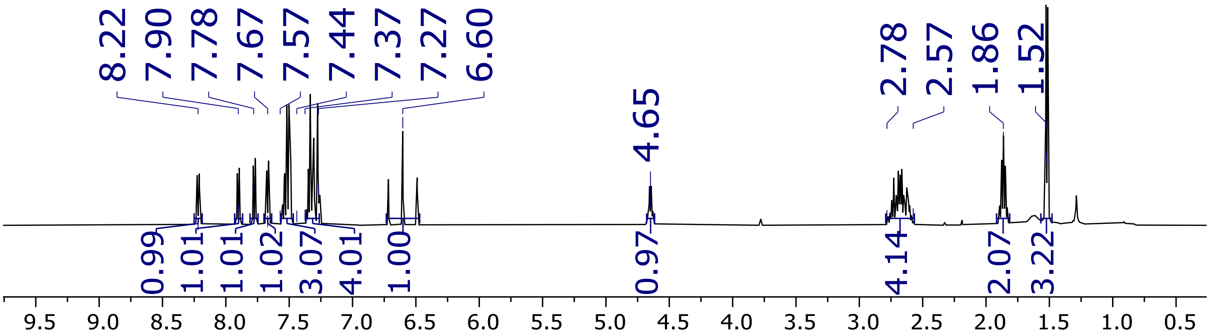

^{13}C NMR (151 MHz, $CDCl_3$):

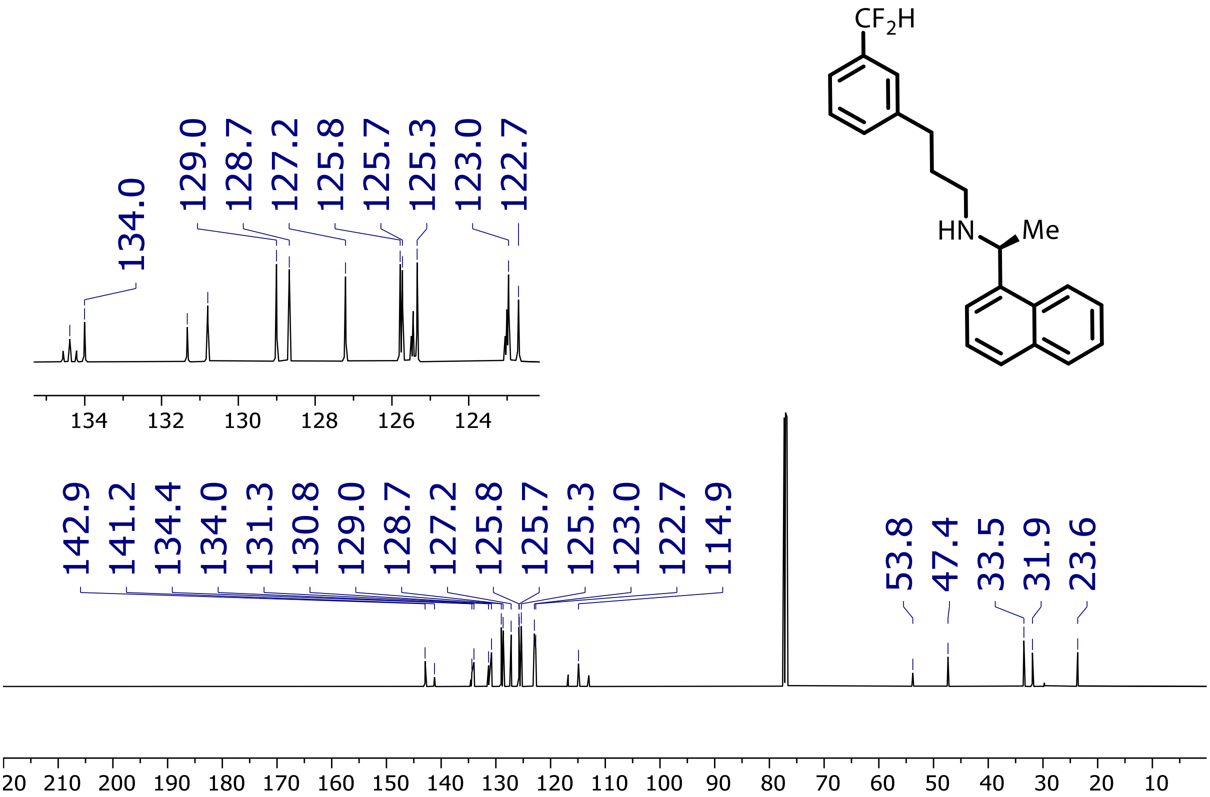

^{19}F NMR (376 MHz, $CDCl_3$):

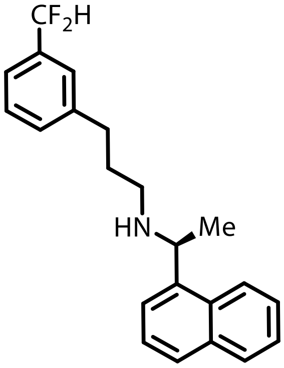

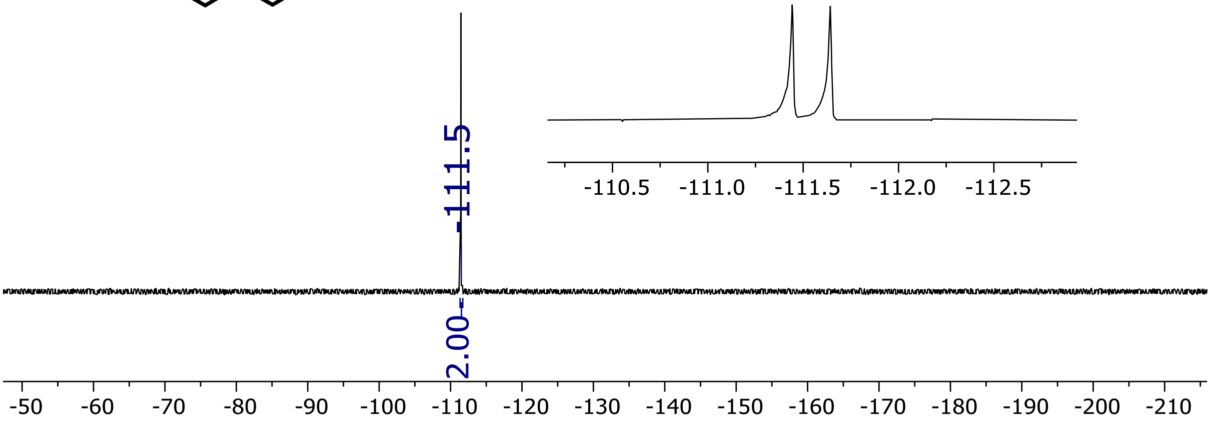

*(1R,5S)-3-Butyl-1-(4-(diifluoromethyl)phenyl)-3-azabicyclo[3.1.0]hexane, **31b***

^{1}H NMR (400 MHz, $CDCl_3$):

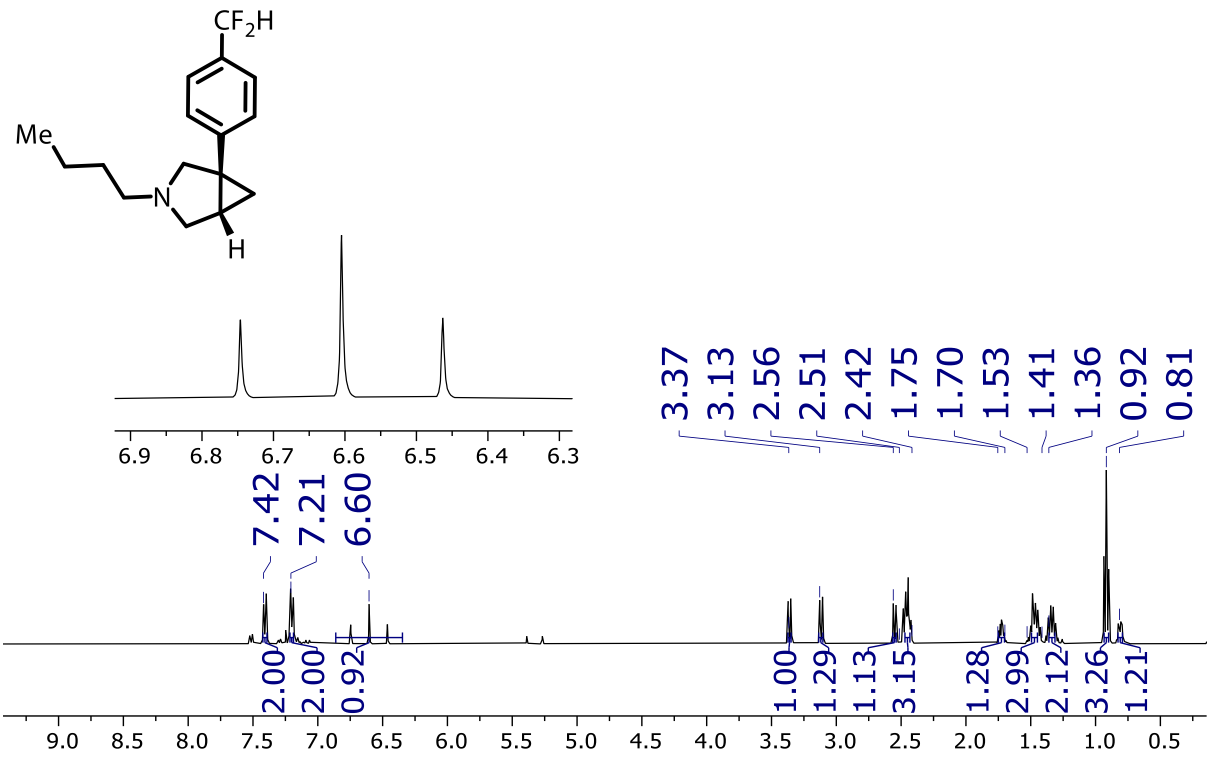

^{13}C NMR (151 MHz, $CDCl_3$):

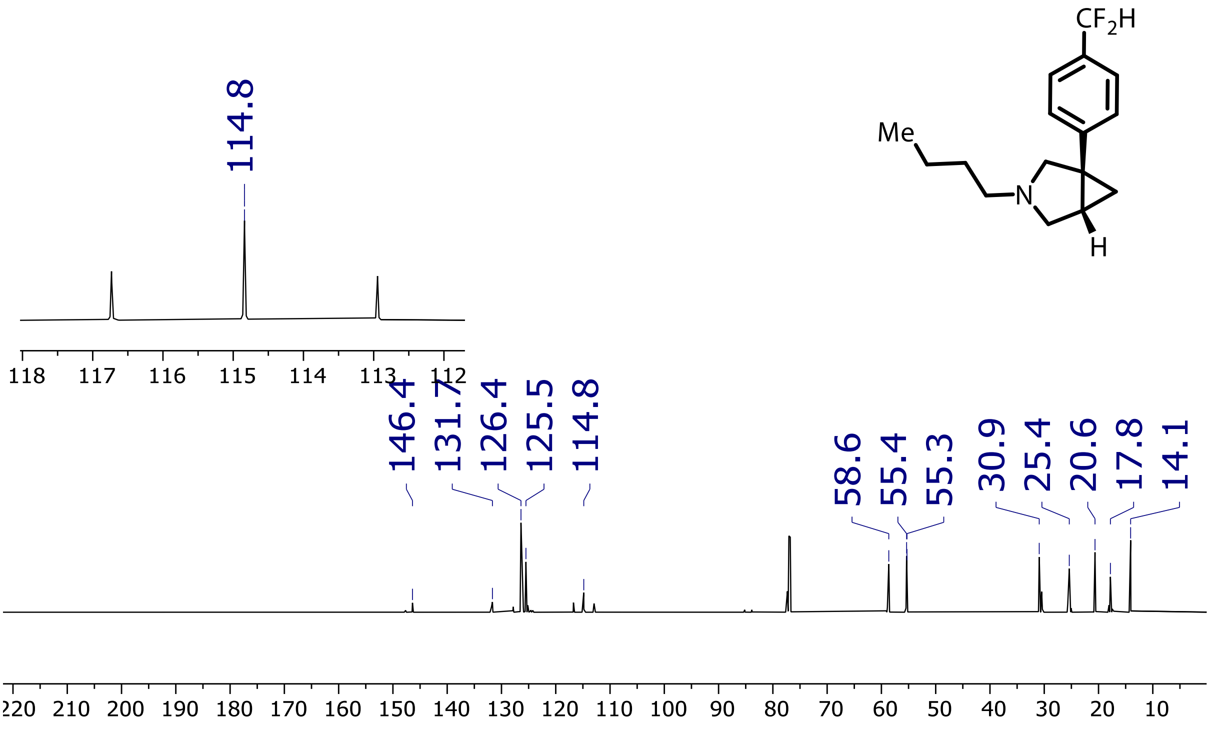

^{19}F NMR (376 MHz, $CDCl_3$):

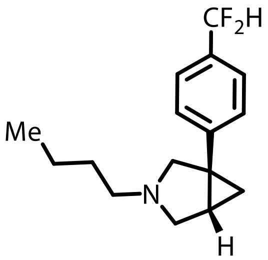

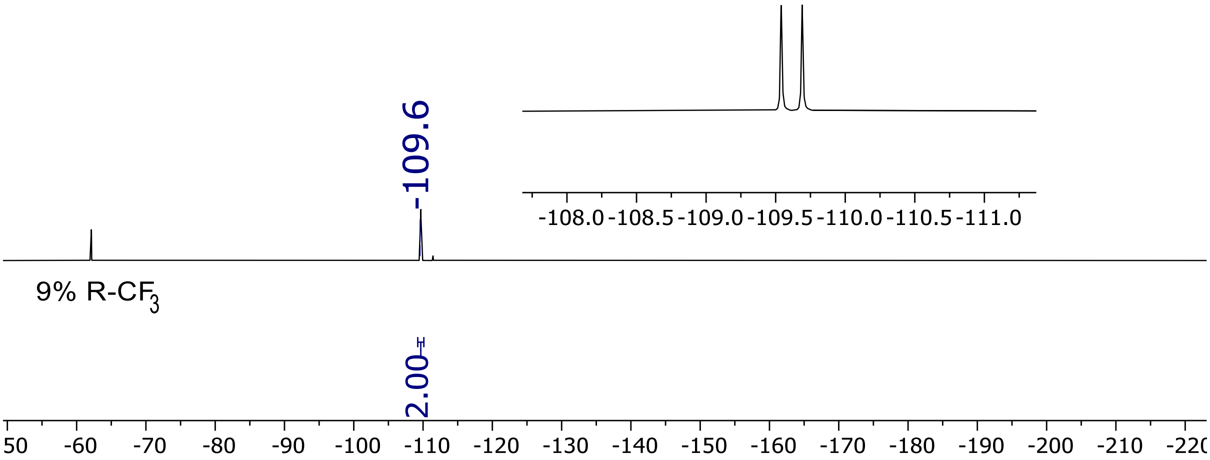

NMR Spectra of Novel Ar-CFH$_2$ Products

4'-(Fluoromethyl)-2,6-dimethyl-1,1'-biphenyl, ***4c***

^{1}H NMR (400 MHz, $CDCl_3$):

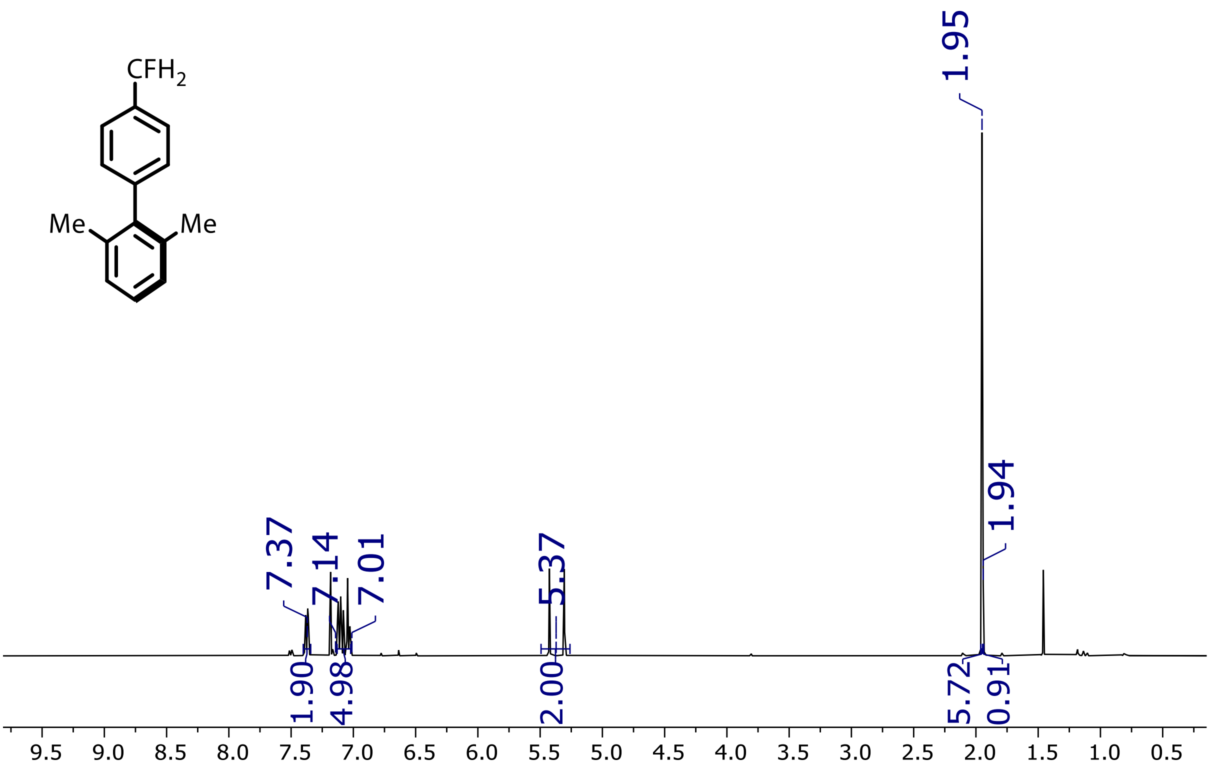

^{13}C NMR (151 MHz, $CDCl_3$):

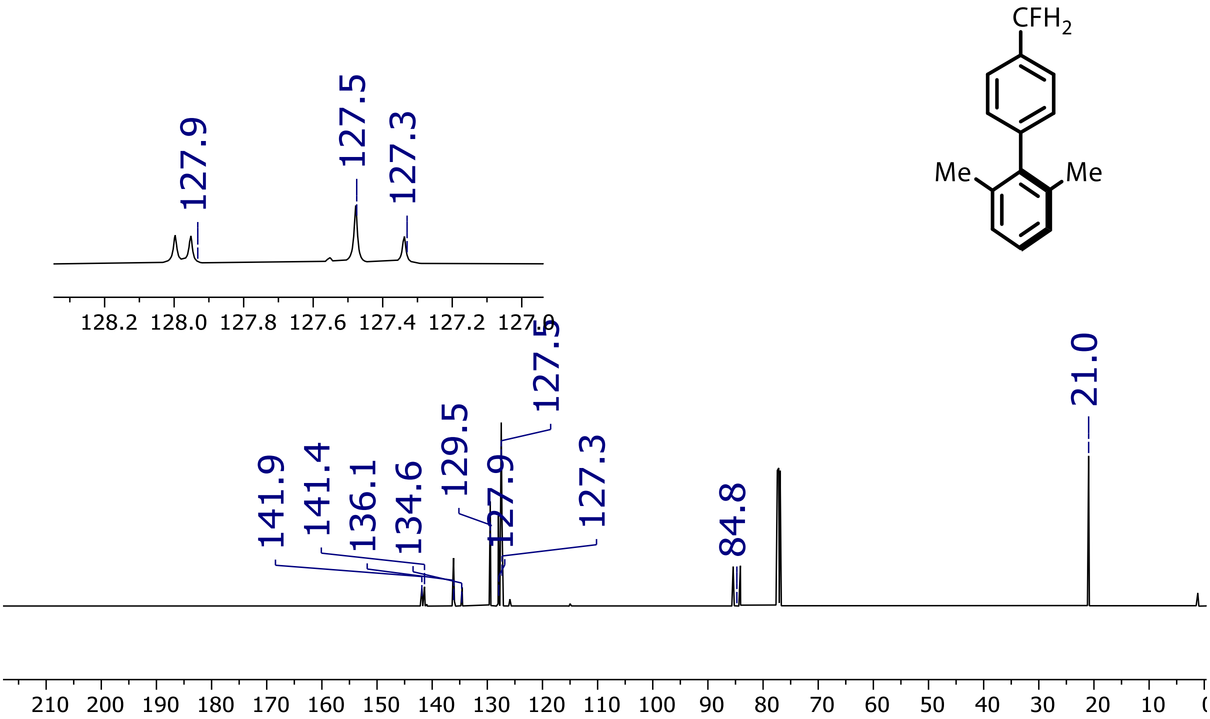

^{19}F NMR (376 MHz, $CDCl_3$):

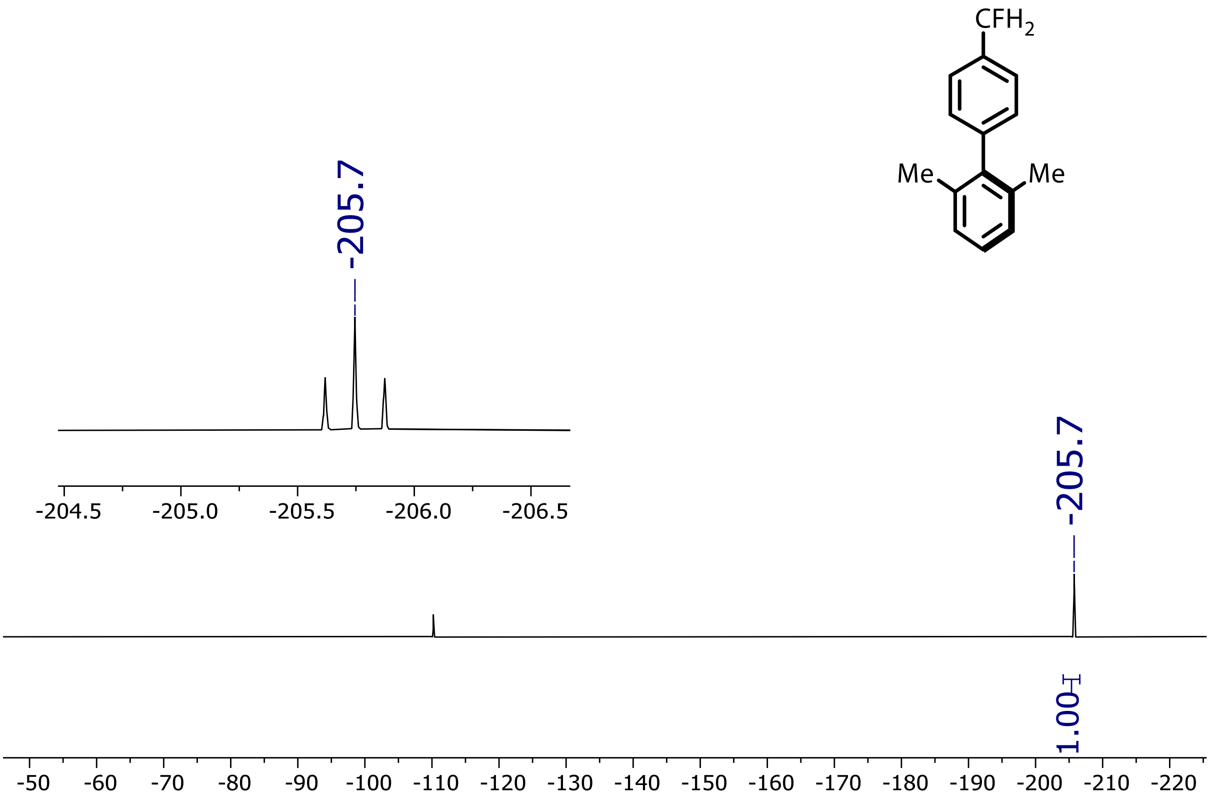

(R)-3-(3-(Fluoromethyl)phenyl)-N-(1-(naphthalen-1-yl)ethyl)propan-1-amine, ***30c***
^{1}H NMR (400 MHz, $CDCl_3$):

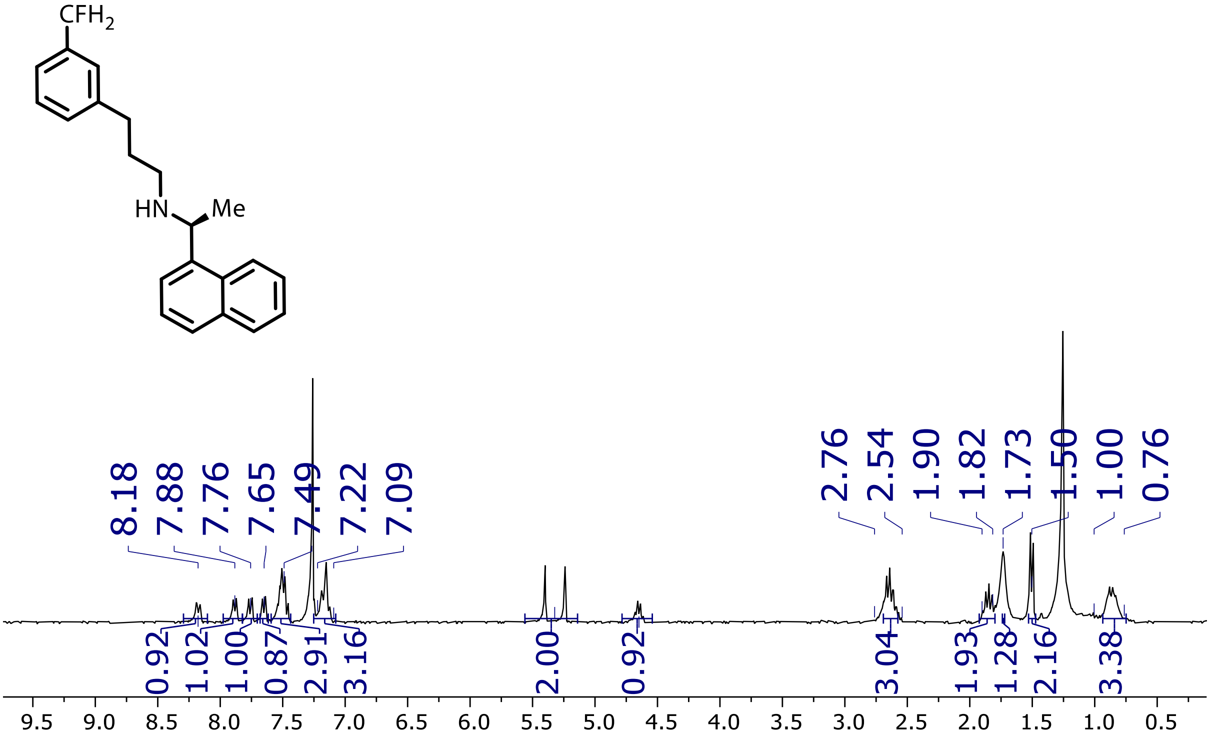

^{13}C NMR (151 MHz, $CDCl_3$):

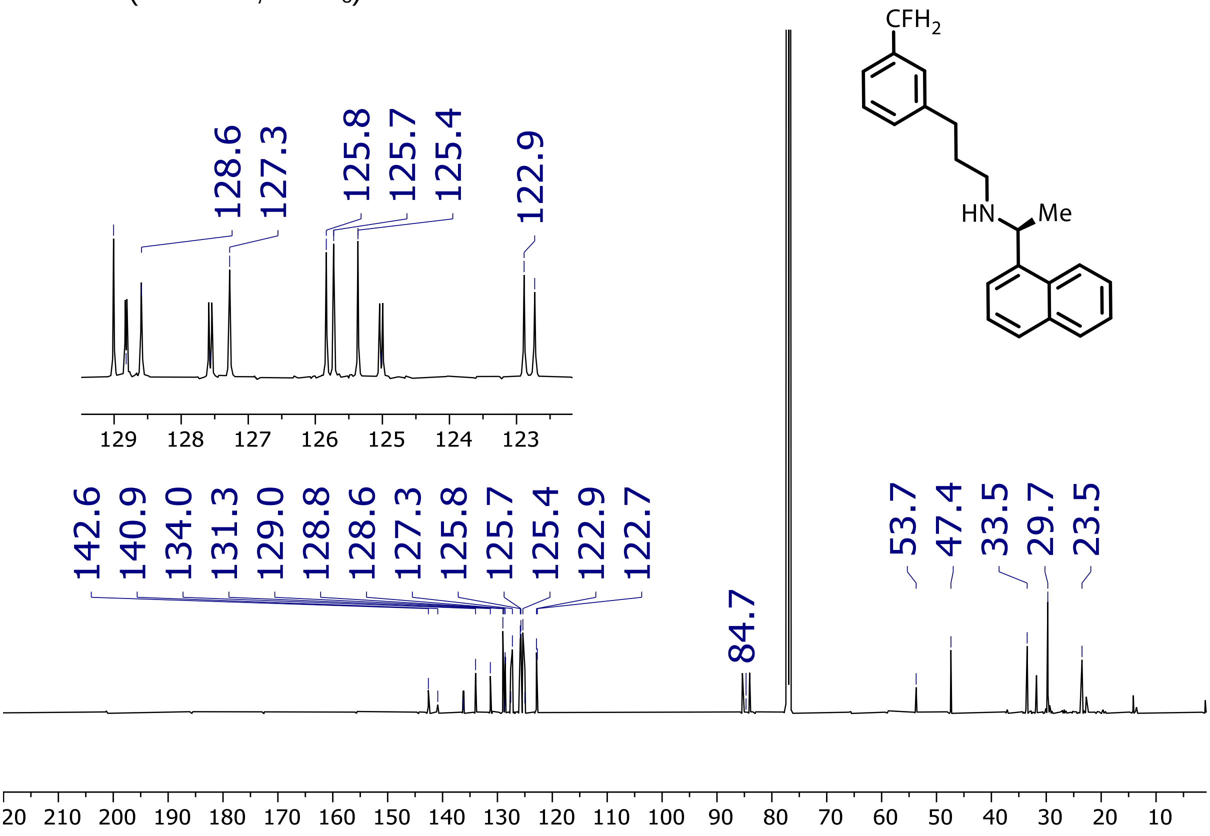

^{19}F NMR (376 MHz, $CDCl_3$):

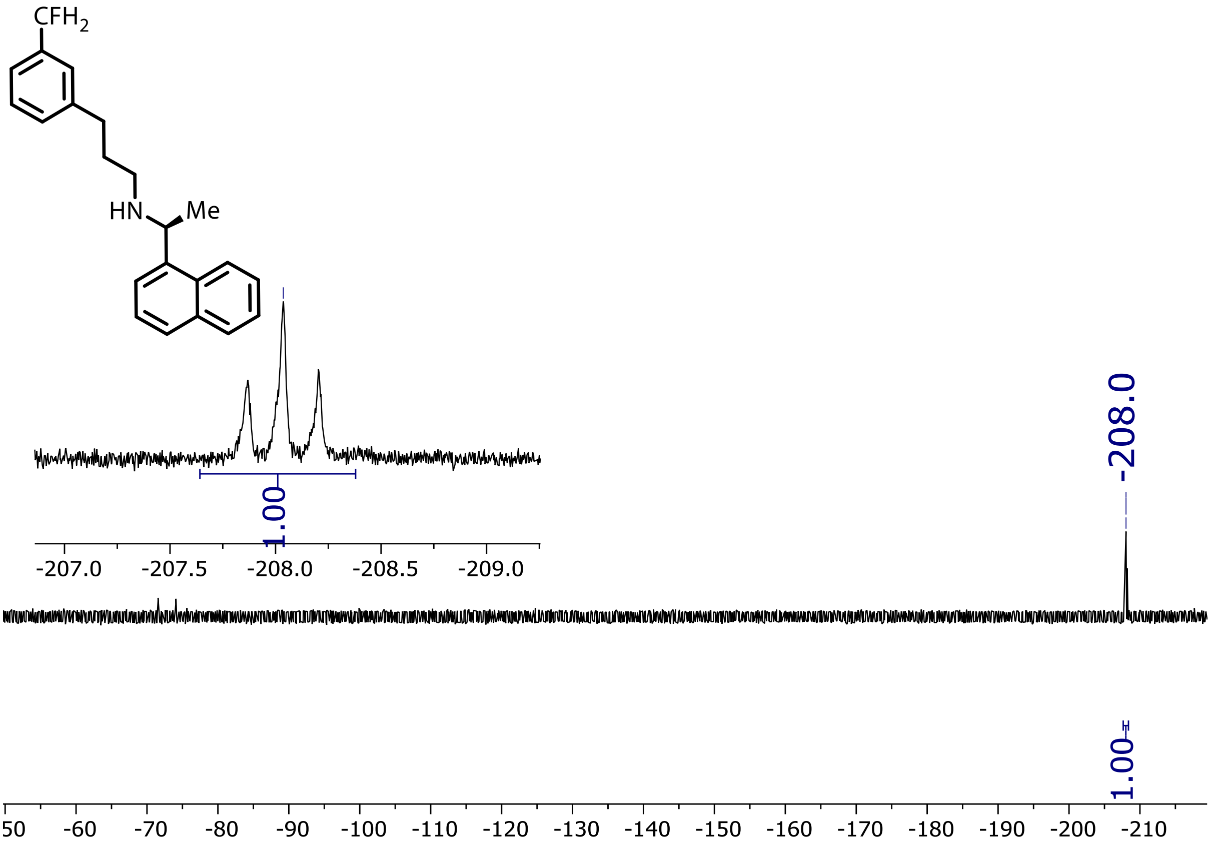

3-(2-(Fluoromethyl)-10H-phenothiazin-10-yl)-N,N-dimethylpropan-1-amine, ***33c***

^{1}H NMR (400 MHz, $CDCl_3$):

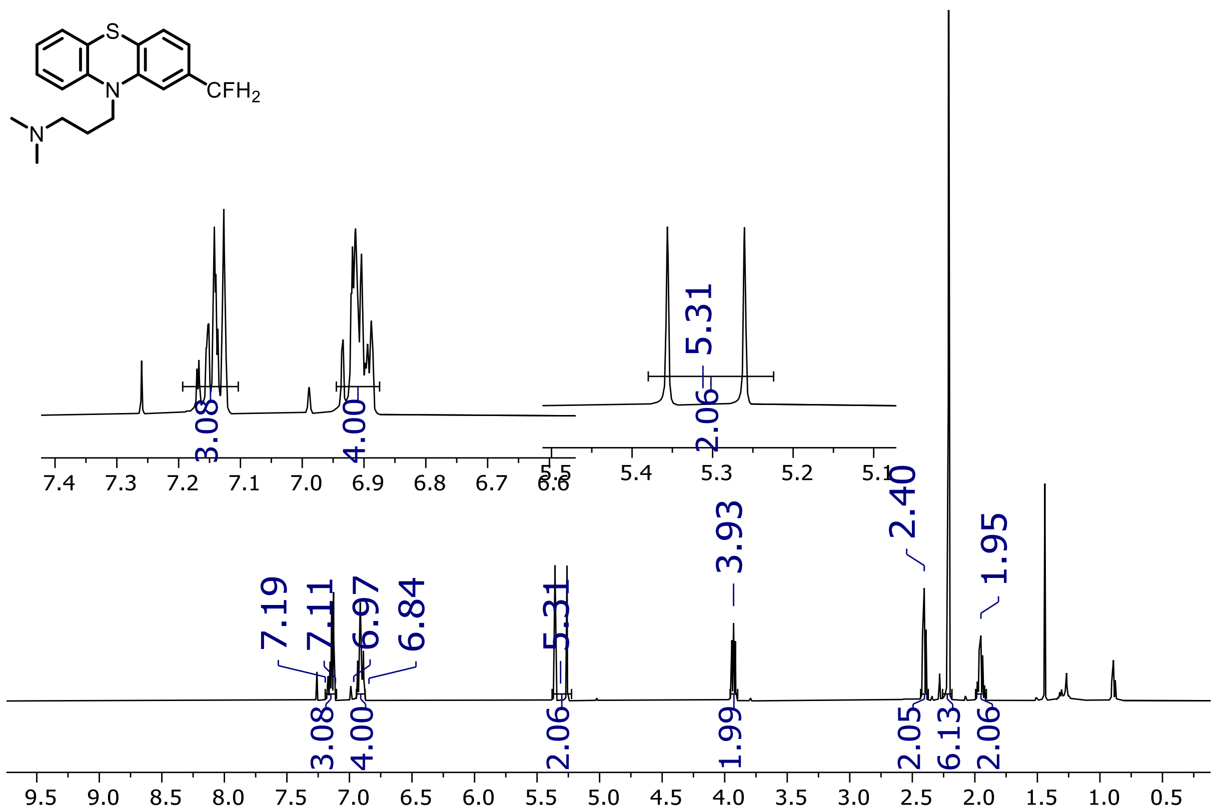

^{13}C NMR (151 MHz, $CDCl_3$):

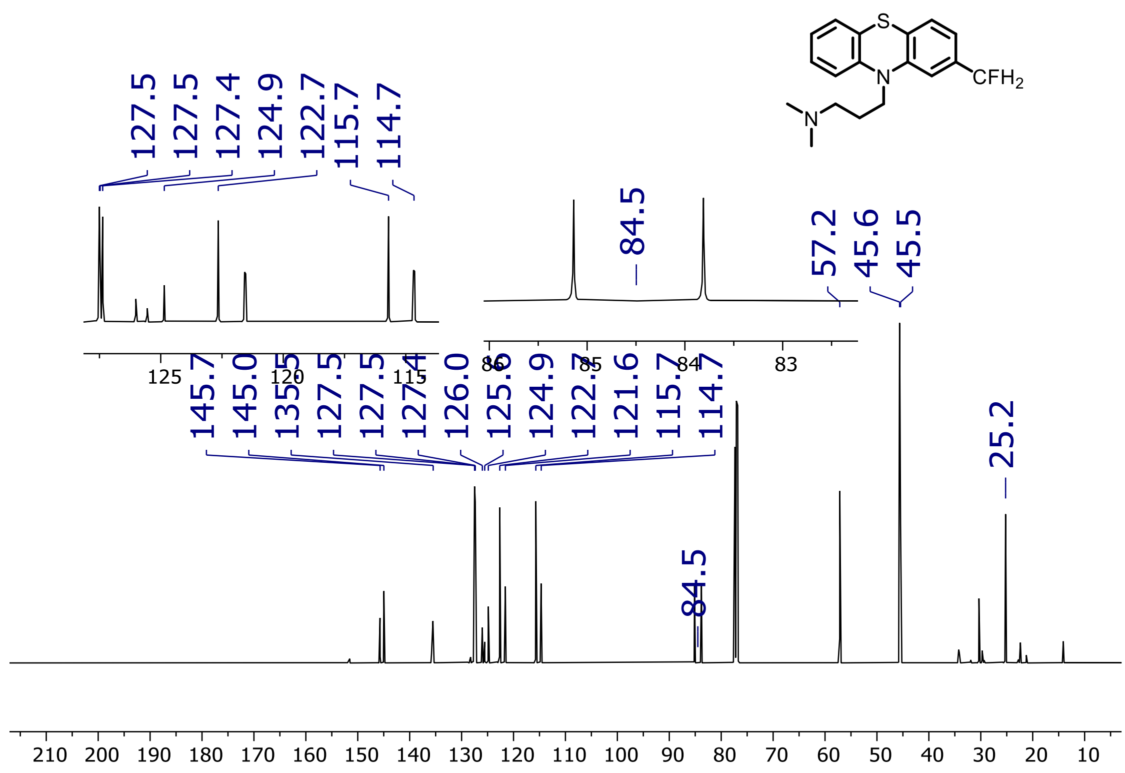

19F NMR (376 MHz, $CDCl_3$):

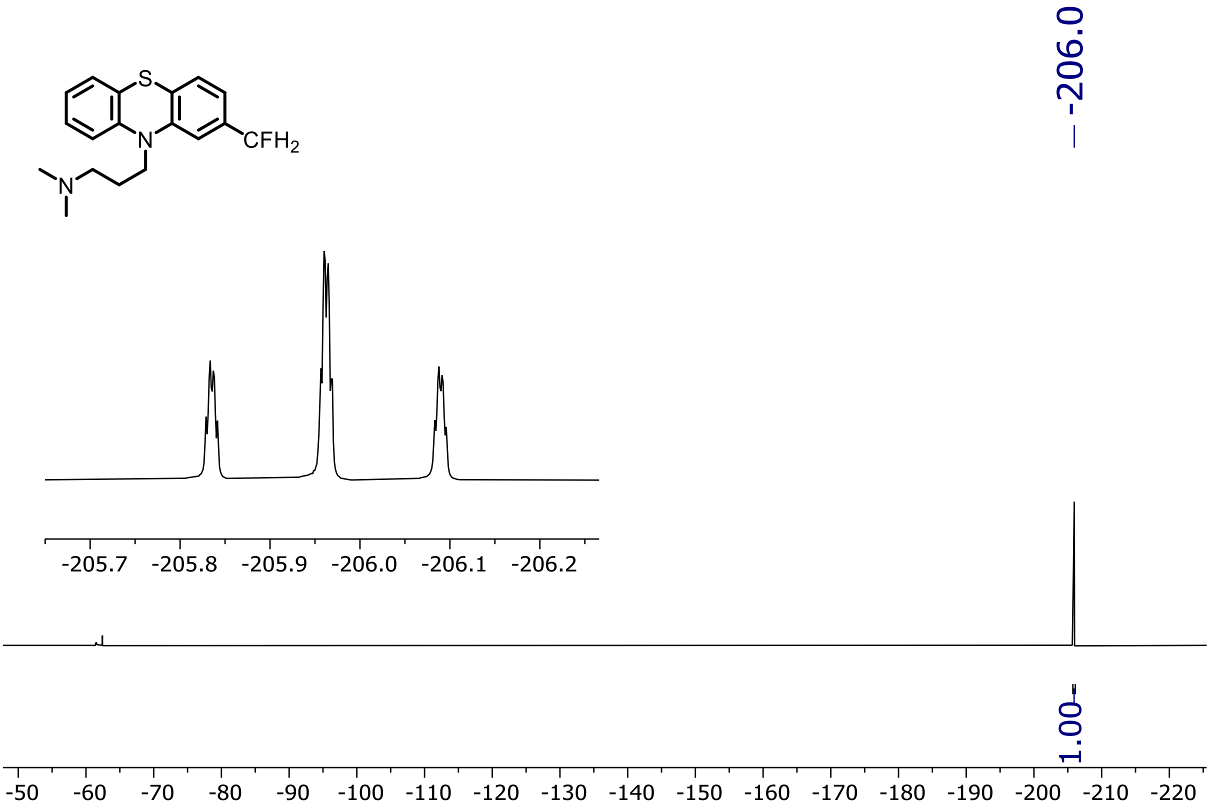

References

[1] C. Liu, K. Li, R. Shang, *ACS Catal.* **2022**, *12*, 4103–4109.

[2] D. B. Vogt, C. P. Seath, H. Wang, N. T. Jui, *J. Am. Chem. Soc.* **2019**, *141*, 13203–13211.

[3] J. B. I. Sap, N. J. W. Straathof, T. Knauber, C. F. Meyer, M. M. Médebielle, L. Buglioni, C. Genicot, A. Andrés, A. Trabanco, T. Noë, C. W. Am Ende, V. V. Gouverneur, *J. Am. Chem. Soc* **2020**, *142*, 53.

[4] S. B. Munoz, C. Ni, Z. Zhang, F. Wang, N. Shao, T. Mathew, G. A. Olah, G. K. S. Prakash, *European J. Org. Chem.* **2017**, *2017*, 2322–2326.

[5] H. Minami, T. Saito, C. Wang, M. Uchiyama, *Angew. Chemie Int. Ed.* **2015**, *54*, 4665–4668.

[6] Y. Deng, F. Lu, S. You, T. Xia, Y. Zheng, C. Lu, G. Yang, Z. Chen, M. Gao, A. Lei, *Chinese J. Chem.* **2019**, *37*, 817–820.

[7] U. Schön, J. Messinger, M. Buckendahl, M. S. Prabhu, A. Konda, *Tetrahedron Lett.* **2007**, *48*, 2519–2525.

[8] G. Manolikakes, A. Gavryushin, P. Knochel, *J. Org. Chem.* **2008**, *73*, 1429–1434.

[9] U. Orcel, J. Waser, *Angew. Chemie Int. Ed.* **2016**, *55*, 12881–12885.

[10] M.-Y. Xu, W.-T. Jiang, Y. Li, Q.-H. Xu, Q.-L. Zhou, S. Yang, B. Xiao, *J. Am. Chem. Soc.* **2019**, *141*, 7582–7588.

[11] M. Huiban, M. Tredwell, S. Mizuta, Z. Wan, X. Zhang, T. L. Collier, V. Gouverneur, J. Passchier, *Nat. Chem.* **2013**, *5*, 941–944.

[12] Y. Huang, Z. Zheng, T. Liu, J. Lü, Z. Lin, H. Li, R. Cao, *Catal. Commun.* **2011**, *14*, 27–31.

[13] Z. Wei, W. Miao, C. Ni, J. Hu, *Angew. Chemie Int. Ed.* **2021**, *60*, 13597–13602.

[14] H. Motohashi, M. Kato, K. Mikami, *J. Org. Chem.* **2019**, *84*, 6483–6490.

[15] K. Hori, H. Motohashi, D. Saito, K. Mikami, *ACS Catal.* **2019**, *9*, 417–421.

[16] N. Betterley, S. Kongsriprapan, S. Chaturonrutsamee, P. Deelertpaiboon, P. Surawatanawong, M. Pohmakotr, D. Soorukram, V. Reutrakul, C. Kuhakarn, *Synthesis (Stuttg).* **2018**, *50*, 2033–2040.

[17] C. Cheng, M. Brookhart, *Angew. Chemie Int. Ed.* **2012**, *51*, 9422–9424.

[18] Y. Gu, X. Leng, Q. Shen, *Nat. Commun.* **2014**, *5*, 5405.

[19] P. S. Fier, J. F. Hartwig, *J. Am. Chem. Soc.* **2012**, *134*, 5524–5527.

[20] Z. Feng, Q.-Q. Min, X.-P. Fu, L. An, X. Zhang, *Nat. Chem.* **2017**, *9*, 918–923.

[21] C. Xu, W. H. Guo, X. He, Y. L. Guo, X. Y. Zhang, X. Zhang, *Nat. Commun. 2018 91* **2018**, *9*, 1–10.

[22] J. Ma, W. Yi, G. Lu, C. Cai, *Org. Biomol. Chem.* **2015**, *13*, 2890–2894.

[23] T. Shishimi, S. Hara, *J. Fluor. Chem.* **2014**, *168*, 55–60.

[24] O. R. Gedde, A. Bonde, P. I. Golbækdal, T. Skrydstrup, *Chem. – A Eur. J.* **2022**, *28*, e202200997.

[25] J. Guo, K. L. Bamford, D. W. Stephan, *Org. Biomol. Chem.* **2019**, *17*, 5258–5261.

[26] K. Dempsey, R. Mir, I. Smajlagic, T. Dudding, *Tetrahedron* **2018**, *74*, 3507–3511.

[27] Z. Zou, H. Li, M. Huang, W. Zhang, S. Zhi, Y. Wang, Y. Pan, *Org. Lett.* **2021**, *23*, 8252–8256.

[28] Y.-M. Su, G.-S. Feng, Z.-Y. Wang, Q. Lan, X.-S. Wang, *Angew. Chemie Int. Ed.* **2015**, *54*, 6003–6007.

[29] H. Yin, J. Sheng, K.-F. Zhang, Z.-Q. Zhang, K.-J. Bian, X.-S. Wang, *Chem. Commun.* **2019**, *55*, 7635–7638.

[30] L. An, Y.-L. Xiao, Q.-Q. Min, X. Zhang, *Angew. Chemie Int. Ed.* **2015**, *54*, 9079–9083.

[31] G. Blessley, P. Holden, M. Walker, J. M. Brown, V. Gouverneur, *Org. Lett.* **2012**, *14*, 2754–2757.

[32] J. R. Box, A. P. Atkins, A. J. J. Lennox, *Chem. Sci.* **2021**, *12*, 10252–10258.

[33] C. Hansch, A. Leo, R. W. Taft, *Chem. Rev.* **1991**, *91*, 165–195.

[34] L. Wang, J. Wei, R. Wu, G. Cheng, X. Li, J. Hu, Y. Hu, R. Sheng, *Org. Chem. Front.* **2017**, *4*, 214–223.

[35] C. D. Sessler, M. Rahm, S. Becker, J. M. Goldberg, F. Wang, S. J. Lippard, *J. Am. Chem. Soc.* **2017**, *139*, 9325–9332.
